# Superoxide Dismutase 1 Nanoparticles (Nano-SOD1) as a Potential Drug for the Treatment of Inflammatory Eye Diseases

**DOI:** 10.3390/biomedicines9040396

**Published:** 2021-04-07

**Authors:** Alexander N. Vaneev, Olga A. Kost, Nikolay L. Eremeev, Olga V. Beznos, Anna V. Alova, Peter V. Gorelkin, Alexander S. Erofeev, Natalia B. Chesnokova, Alexander V. Kabanov, Natalia L. Klyachko

**Affiliations:** 1 School of Chemistry, Lomonosov Moscow State University, 119991 Moscow, Russia; vaneev.aleksandr@gmail.com (A.N.V.); kost-o@mail.ru (O.A.K.); eremeev@enzyme.chem.msu.ru (N.L.E.); Erofeev@polly.phys.msu.ru (A.S.E.); kabanov@enzyme.chem.msu.ru (A.V.K.); 2 Research Laboratory of Biophysics, National University of Science and Technology “MISIS”, 119991 Moscow, Russia; peter.gorelkin@gmail.com; 3 Helmholtz National Medical Research Center of Eye Diseases, 105062 Moscow, Russia; olval2011@mail.ru (O.V.B.); nchesnokova2012@yandex.ru (N.B.C.); 4 School of Biology, Lomonosov Moscow State University, 119991 Moscow, Russia; ava1945@mail.ru; 5 Eshelman School of Pharmacy, University of North Carolina at Chapel Hill, Chapel Hill, NC 27599, USA; 6 Research Institute “Nanotechnology and Nanomaterials”, G.R. Derzhavin Tambov State University, 392000 Tambov, Russia

**Keywords:** nanoparticles, Nano-SOD1, inflammation, uveitis, eye diseases, double-layered polyelectrolyte complex, drug delivery

## Abstract

Inflammatory eye diseases remain the most common clinical problem in ophthalmology. The secondary processes associated with inflammation, such as overproduction of reactive oxygen species (ROS) and exhaustion of the endogenous antioxidant system, frequently lead to tissue degeneration, vision blurring, and even blindness. Antioxidant enzymes, such as copper–zinc superoxide dismutase (SOD1), could serve as potent scavengers of ROS. However, their delivery into the eye compartments represents a major challenge due to the limited ocular penetration. This work presents a new therapeutic modality specifically formulated for the eye on the basis of multilayer polyion complex nanoparticles of SOD1 (Nano-SOD1), which is characterized by appropriate storage stability and pronounced therapeutic effect without side reactions such as eye irritation; acute, chronic, and reproductive toxicity; allergenicity; immunogenicity; mutagenicity even at high doses. The ability of Nano-SOD1 to reduce inflammatory processes in the eye was examined in vivo in rabbits with a model immunogenic uveitis—the inflammation of the inner vascular tract of the eye. It was shown during preclinical studies that topical instillations of Nano-SOD1 were much more effective compared to the free enzyme in decreasing uveitis manifestations. In particular, we noted statistically significant differences in such inflammatory signs in the eye as corneal and conjunctival edema, iris hyperemia, and fibrin clots. Moreover, Nano-SOD1 penetrates into interior eye structures more effectively than SOD itself and retains enzyme activity in the eye for a much longer period of time, decreasing inflammation and restoring antioxidant activity in the eye. Thus, the presented Nano-SOD1 can be considered as a potentially useful therapeutic agent for the treatment of ocular inflammatory disorders.

## 1. Introduction

Reactive oxygen species (ROS) are excessively produced in many disease states and contribute to cell death and tissue degeneration due to the damage of multiple components of cells including cell membranes, DNA, proteins (including various enzymes), carbohydrates, and proteinase inhibitors. Under physiological conditions, a balance exists between the level of ROS produced during normal cellular metabolism and the level of endogenous antioxidants that offers protection against damage caused by ROS. Disruption of this balance, through increased ROS production or decreased antioxidant levels, causes a condition referred to as oxidative stress that is associated with an increased risk of cardiovascular disease, atherosclerosis, cancer, and many other diseases. In particular, ROS are important factors in the early tissue damage that develops from inflammation [1,2]. Because of the nonspecific nature of ROS-induced tissue injury, excessive release of these agents can cause substantial damage not only to the tissue in an inflamed state but also to the surrounding normal tissue. This is very important for the eye, as the transparency of the cornea and lens, as well as the functioning of photoreceptor apparatus, relies on their highly ordered structures, and excessive tissue damage will compromise visual function [3].

Antioxidants, superoxide dismutase 1 (SOD1) in particular, are known to play a beneficial role in protecting tissues against oxidative stress in many pathological states, including inflammation [4,5,6,7,8,9,10,11,12]. Thus, SOD1 was reported to accelerate the healing of skin lesions caused by burns, systemic lupus erythematosus, and herpes [13,14]; protect cultured human neurons under oxidative stress [15]; reduce ischemia-reperfusion injury [16]; be effective in the treatment of rat adjuvant arthritis, [17] etc.

Most relevant to this study, antioxidants, including SOD1, are also thought to be beneficial in the treatment of eye diseases. Superoxide dismutase 1 was found in different eye tissues and fluids [18,19,20], where this enzyme plays an important role in confronting oxidative stress and maintaining eye homeostasis. The eye is a rather isolated organ, and the pathological processes within it are preferably treated not via systemic but by local drug intake. The use of topical applications, subconjunctival, parabulbar or intraocular injections of SOD1 could, therefore, provide a supplement for intrinsic antioxidants in eye tissues, which may be depleted at oxidative stress. According to statistics, inflammatory eye diseases, accompanied by oxidative stress, are the most common eye pathologies that lead to partial disability and, sometimes, to the complete loss of vision [21,22,23]. It was shown that both subconjunctival injections and topical applications of SOD1 solutions were effective in preventing corneal perforations under acute inflammation of the eye after alkali burns [24,25]. Other beneficial effects of SOD1 were obtained in the treatment of uveitis inflammation of the uveal tract involving both outer and inner structures of the eye. Thus, SOD1 treatment of animals with phacoanaphylactic endophthalmitis (lens-induced uveitis) resulted in the strong reduction of choroid inflammation, retinal edema and, vasculitis [26]. In S-antigen-induced and bovine serum albumin-induced passive Arthus-type uveitis, SOD1 treatment resulted in the remarkable reduction in aqueous humor cell quantity and infiltration of the inflammatory cells in the anterior retina [25,27]. Topical instillations of SOD1 at immunogenic uveitis induced by foreign animal serum resulted in the enhancement of general antioxidant activity in the eye and in the substantial decrease of leukocyte count in aqueous humor, even in comparison with dexamethasone [28,29].

However, topical application of the drug, although sparing for the patient, is less effective for the treatment of the diseases, which include inner structures of the eye, due to the poor transport of the drug into the eye. In recent years, there has been significant interest in the development of nanosize drug delivery systems to the eye. Such nanosystems can potentially improve the therapeutic efficacy of the drugs by overcoming the diffusion barrier, by increasing their stability in biological tissues and fluids, and enhancing cellular/tissue uptake [30,31,32,33,34,35].

Several years ago, a cross-linked polyion complex of SOD1 with a cationic block copolymer methoxy-poly(ethylene glycol)-block(L-lysine hydrochloride) (PEG-pLL_50_) was developed [36]. This SOD1 nanoformat decreased ischemia/reperfusion-induced tissue injury and improved sensorimotor functions in a rat middle cerebral artery occlusion model after a single intravenous injection, [36] attenuated hypertension established in mice by chronic subcutaneous infusion of angiotensin II after intracerebroventricular injection [37], demonstrated a significant decrease in markers in endothelial/vascular cell activation and/or inflammation in visceral adipose tissue, thoracic aorta and heart of obese mice [38], and attenuated ethanol-induced steatohepatitis [39] after intraperitoneal injections. It is noteworthy that this nanoformulated SOD1 also appeared to be more effective than native SOD1 in the treatment of inflammatory eye disease, uveitis, just at topical instillations [40]. Despite all the advantages of this SOD1 formulation in treatment, it should be noted that during the synthesis of the drug, the loss of enzyme activity exceeds 90%.

Recently, a new SOD1 nanoformulation was introduced, based on a chemically cross-linked multilayer polyion complex in which SOD1 is initially incorporated into a complex with polycation, then coated with a polyanion block copolymer. The loss of the enzyme activity during the synthesis by this technique was significantly lower (60–70%), and this double-coated SOD1 nanoparticles exhibited prolonged circulation of active enzymes in the blood stream, while single intravenous injection of such a nanoformulation improved the recovery of locomotor functions in rats with moderate spinal cord injury [41].

In the current study, we adjusted the synthesis of double-coated SOD1 nanoparticles (Nano-SOD1) specified for local application in ophthalmology and evaluated their effectiveness in reducing inflammation in the eye.

## 2. Materials and Methods

### 2.1. Materials

Recombinant human SOD1 was purchased from Life Science Advanced Technologies (St. Petersburg, Russia). Methoxy-poly(ethylene glycol)_113_-block-poly(L-glutamic acid sodium salt)_50_ (PGlu-PEG) were purchased from Alamanda Polymers Inc. (USA). Protamine from salmon, glutaraldehyde (GA), 0.01 М phosphate-buffered saline (PBS, pH 7.4, 0.137 М NaCl), quercetin, pyrogallol, Amicon Ultra Centrifugal Filter Units (membrane with a molecular weight cut off of 100 kDa), hemoglobin from bovine blood, luminol, 6-hydroxy-2,5,7,8-tetramethylchromane (Trolox), and a sterile syringe filter with a 220 nm pore size were purchased from Merck Milipore (Carrigtwohill, Ireland). Dialysis capsules, Float-A-Lyzer^®^ G2 with a membrane with a molecular weight cut off of 100 kDa, were purchased from Spectrum Laboratories (CA, USA). A protein concentration assay kit and fluorescent dye, Alexa 488 Fluor, were from Thermo Fisher Scientific (CA, USA).

### 2.2. Synthesis of Double-Coated Cross-Linked Nano-SOD1

The Nano-SOD1 was synthesized using a modified procedure of Nukolova et al. [41]. Briefly, stock solutions of SOD1, protamine, and PGlu-PEG (5 mg/mL each) were prepared in PBS or 10 mM 2-[4-(2-hydroxyethyl)piperazin-1-yl]ethanesulfonic acid (HEPES), pH 7.4, containing 0.137 M NaCl. Stock solutions of GA (0.5%, *v/v*) and NaBH_4_ (1 mg/mL) were prepared in water immediately before the experiments. All solutions were added to each other dropwise with stirring. First, protamine solution was added to the SOD1 solution to achieve a final SOD1/polycation charge ratio 1:2 of ionogenic groups, and the resulting solution was incubated at room temperature (r.t.) for 30 min upon stirring. The charge ratio was calculated as a ratio of the concentration of NH_2_ groups of the protamine protonated at pH 7.4 to the concentration of COOH groups of glutamic acid and aspartic acid residues of the enzyme (estimated using Protein Calculator v.3.3 software, Scripps Research Institute, CA, USA). Subsequently, PGlu-PEG solution was added to the SOD1/protamine complex solution at a final charge ratio polycation/polyanion 1:1, and the reaction mixture was incubated for 30 min at 4 °C without stirring. Then, GA solution was added to achieve a final molar ratio of cross-linker to amino groups of protamine 4.4:1. The resulting mixture was then incubated overnight at 4 °C, formed Schiff base bonds were reduced by the treatment with NaBH_4_ solution. To remove the side products and excess of unreacted reagents, the reaction mixture was diluted 4 times by PBS or 10 mM HEPES and centrifuged on 100 kDa Amicon filters at 800× *g*, 4 °C. The procedure was repeated twice. Purified samples of cross-linked Nano-SOD1 were adjusted to specific SOD1 activity 13,500 U by pyrogallol (185,000 U by quercetin) per mL, stored at 4 °C, and used in experiments on animals.

### 2.3. Protein Concentration and Enzyme Activity

The protein concentration was measured using the Micro BCA Protein Assay Kit in accordance with the manufacturer’s instructions (Thermo Fisher Scientific, CA, USA).

The catalytic activity of SOD1 and Nano-SOD1 was determined using pyrogallol or quercetin assay [41]. For the pharmacokinetics studies, the quercetin assay was applied due to its greater sensitivity.

### 2.4. Dynamic Light Scattering (DLS)

Purified Nano-SOD1 was characterized by DLS using Zetasizer Nano ZS (Malvern Instruments Ltd., Malvern, UK). The hydrodynamic diameter and polydispersity index (PDI) were measured in PBS buffer; zeta (ζ)-potential was analyzed in water (the change of solvent was performed by gel filtration on NAP-10 column, GE Healthcare, CA, USA). All measurements were performed in automatic mode at 25 °C at least in triplicate.

### 2.5. Retention of SOD1 in Nano-SOD1

The SOD1 retention in the Nano-SOD1 formulation was determined using 100 kDa dialysis capsules. A capsule was filled with a solution of Nano-SOD1 (1 mL), then the capsule was placed into PBS buffer (30 mL), and the enzyme activity and protein concentration were measured in aliquots (100 µL) taken from the capsule at predetermined intervals using the techniques described above.

### 2.6. Stability of Nano-SOD1

To study the stability of the obtained Nano-SOD1, solutions of SOD1 nanoparticles (13,500 U/mL by pyrogallol) were filtered under sterile conditions through a syringe membrane with 220 nm pores, sealed in glass ampoules, and stored at 4 and 25 °C. Samples were taken at regular intervals (7, 30, 60, and 180 days), and the enzymatic activity and hydrodynamic diameter of Nano-SOD1 were measured.

### 2.7. Animals

A study was conducted using 45 adult male Chinchilla rabbits weighing 2.0–2.5 kg. All experiments with live rabbits were carried out in strict accordance with the Association for Research in Vision and Ophthalmology (ARVO) statement for the Use of Animals in Ophthalmic and Vision Research. The animals were kept in individual cages with free access to food and water.

### 2.8. Dynamics of Labeled SOD1 and Nano-SOD1 in Tear and Intraocular Fluid

For pharmacokinetics study, Nano-SOD1 was synthesized with SOD1 covalently conjugated to Alexa 488 fluorescent dye (in accordance with the manufacturer’s instructions).

Rabbits were randomly divided into 3 groups (*n* = 2 per each group, i.e., 4 eyes): (1) placebo group that received PBS; (2) SOD1 group that received a solution of native SOD1 labeled by Alexa; (3) experimental Nano-SOD1 group that received a solution of SOD1 nanoparticles prepared with labeled SOD1. Specific activities of SOD1 and Nano-SOD1 solutions were equal. Rabbits received 40 μL of drug solutions as eye drops bilaterally twice with an interval of 5 min (total of 80 μL per eye).

Tear fluid was taken 5, 30, and 60 min after the last instillation using round pieces of filter paper with a diameter of 5 mm. Paper pieces were placed into the lower conjunctival sack for 5 min, 6 circles to each eye. Pieces soaked with tear fluid were removed, placed into 300 μL of PBS buffer for 20 min for the elution of tear components, and centrifuged for 10 min at 940× *g*.

The intraocular fluid was collected by paracentesis of the cornea with an insulin syringe under topical anesthesia (Alcain 0.5%, Alcon, Puurs, Belgium) 30 and 60 min after the last instillation.

The intensity of Alexa-labelled SOD1 and NanoSOD1 fluorescence and enzymatic activity was measured in the samples of tear and intraocular fluid.

In addition, we applied the amperometric method to determine the H_2_O_2_ level as an indicator of Nano-SOD1 functioning in tear and intraocular fluid. For this purpose, we used platinum-coated carbon nanoelectrodes in the fluids and a silver chloride reference electrode. Patch-clamp amplifier Model 2400 (AM Systems; WA, USA) allowed registration of the potential difference between platinum nanoelectrode and reference electrode. The USB-6211 ADC/DAC converter (National instruments; USA) and WinWCP software allowed data transfer and recording. The H_2_O_2_ was evaluated at the potential of +800 mV to the silver chloride electrode [42,43,44].

Rabbits were randomly divided into 5 groups (*n* = 5 per group), one group per time point. The Nano-SOD1 solution was instilled into the left eye of each animal, while PBS buffer (control) was instilled into the right eye. The samples of tear and intraocular fluid were taken (as described in previously) before instillation and 10, 30, 60, and 120 min after the instillation, and amperage in the sample (proportional to Н_2_О_2_ level) was measured by platinized electrode. Due to the variability of the background levels of hydrogen peroxide in the tear and intraocular fluid of each animal, the H_2_O_2_ dynamics were calculated as the difference between the left (experimental) and right (control) eyes of each animal.

### 2.9. In Vivo Studies/Treatment of Immunogenic Uveitis

Experiments were performed in three independent series with 15 rabbits each.

Acute uveitis was induced in rabbits by double injection of normal equine serum: initial subcutaneous injection of 5 mL serum for sensitization was followed on the 10th day with intravitreal injection of 70 μL serum after anesthetic Alcain 0.5% (Alcon, Puurs, Belgium) instillation [45].

Animals were randomly divided into 3 groups (*n* = 5 per group, i.e., 10 eyes) and treated as follows: (1) placebo group with uveitis that received PBS buffer (placebo); (2) SOD1 group with uveitis that received native SOD1 solution (1 mg/mL, 13,500 U/mL with pyrogallol assay) in the same buffer; (3) Nano-SOD1 group with uveitis that received Nano-SOD1 solution in the same buffer. During the treatment, solutions were instilled three times a day (30 μL in each eye) for 8 days. The SOD1 dose corresponded to that used in Reference [40]. The dose of Nano-SOD1 solution was calculated based on the specific activity of SOD1 (units per mL) so that the activities of SOD1 and Nano-SOD1 solutions were equal.

Clinical manifestation of uveitis was followed on 1, 2, 3, 4, 7, and 8 days after the intravitreal injection of equine serum. Rabbit eyes were observed under the slit lamp and scored for clinical signs of ocular inflammation. Clinical symptoms of uveitis included eyelid and conjunctival edema and hyperemia, corneal and iris edema, presence of fibrin clots in the anterior chamber, and presence of cataract and posterior synechiae (cohesions between the papillary margin of the iris and anterior part of the lens). We compared the efficacy using the scores for the manifestations of inflammation as a common approach in ophthalmology [46]. Evaluation of the inflammation score was performed using a conventional scale: (0) no symptom, (1) low degree of manifestation, (2) medium, and (3) strong.

### 2.10. Leukocyte Counting and Antioxidant Activity in Intraocular Fluid

Intraocular fluid from all eyes was collected by paracentesis of the cornea with the insulin syringe under local anesthesia (Alcain 0.5%, eye drops) on the 8th day (i.e., 16 h after the last instillation) for leukocyte counting and total protein and antioxidant activity (AOA) measurement. A healthy group of rabbits without uveitis (*n* = 5) was used as a control for a comparative analysis of biochemical parameters during the treatment.

Leukocytes in intraocular fluid were counted in unstained preparation (20 µL) of freshly collected sample using a light microscope. Average amount of leukocytes in the microscopic field was counted.

Antioxidant activity (AOA) was assayed via estimation of chemiluminescence kinetics parameters in the hemoglobin–H_2_O_2_–luminol model system [47] using Trolox as the standard. The AOA value of the sample was expressed as the Trolox equivalent concentration calculated on the basis of a standard curve. Protein concentration in the intraocular fluid was determined by the Lowry method [48].

### 2.11. Toxicology Studies

All toxicity studies were performed according to Reference [49].

Acute toxicity of Nano-SOD1 formulations was determined on two types of animals, rats and rabbits. In series 1, Nano-SOD1 was injected in the lateral vein of rat tails (average rat weight 280 g) in average doses 91,000 U/kg (here and after, units of activity by pyrogallol are given) that exceeded the recommended dose for humans (89 U/kg per day [40] by more than 1000 times. In series 2, Nano-SOD1 was instilled in each eye of the rats in a total dose of 240,000 U/kg that exceeded the recommended dose for humans by 2700 times. In series 3, Nano-SOD1 was instilled in each eye of the rabbits in a dose of 26,000 U/kg (average rabbit body weight of 2.5 kg) that exceeded the recommended dose for humans nearly 300 times.

Chronic toxicity of Nano-SOD1 was investigated on rats and rabbits who received instillations of the formulation with a daily dose exceeding the recommended dose for humans 30 times for rats and 17 times for rabbits during a month.

Assessment of the mutagenic properties of Nano-SOD1 was carried out in tests in vitro for the induction of gene mutations in *Salmonella typhimurium* (Ames test) and in the test in vivo for the induction of chromosomal damage (test accounting micronuclei in polychromatophilic red blood cells of mice). In the latter case, the excess of the dose over the recommended one for humans was 570 times with intraperitoneal administration and 20 times with instillations, accordingly.

To determine the allergenic properties of Nano-SOD1, anaphylactogenic activity assessment, “delayed”-type hypersensitivity reactions, conjunctival tests, and mast cell degranulation were examined in guinea pigs with a 10 fold excess of the dose recommended for humans.

To determine the immunotoxic effect of Nano-SOD1 formulations, an assessment of the humoral immune response, cellular immunity, and phagocytic activity of peritoneal macrophages were examined in mice in excess of the recommended dose for humans up to 20 times.

The influence of Nano-SOD1 on the reproduction was investigated in mice. Formulation was instilled daily—males for 48 days (spermatogenesis period), females for 15 days (3 estrous cycles)—with a 10 fold excess of the dose recommended for humans.

Detailed methods of toxicological studies are presented in the Supplementary Materials.

### 2.12. Data Analysis

All experiments were conducted independently at least in triplicate, the results were expressed as mean value ± standard deviation (SD). Origin 2016 (OriginLab Corporation, MA, USA) and STATISTICA 6 (StatSoft, Inc., OK, USA) were used for statistical analysis. Significance was analyzed using the Mann–Whitney U test and ANOVA test. A *p*-value < 0.05 was considered statistically significant; *p* < 0.01 and *p* < 0.001 were considered highly statistically significant.

## 3. Results and Discussion

### 3.1. Synthesis of SOD1 Nanoparticles and Their Characterization

**Figure 1:**
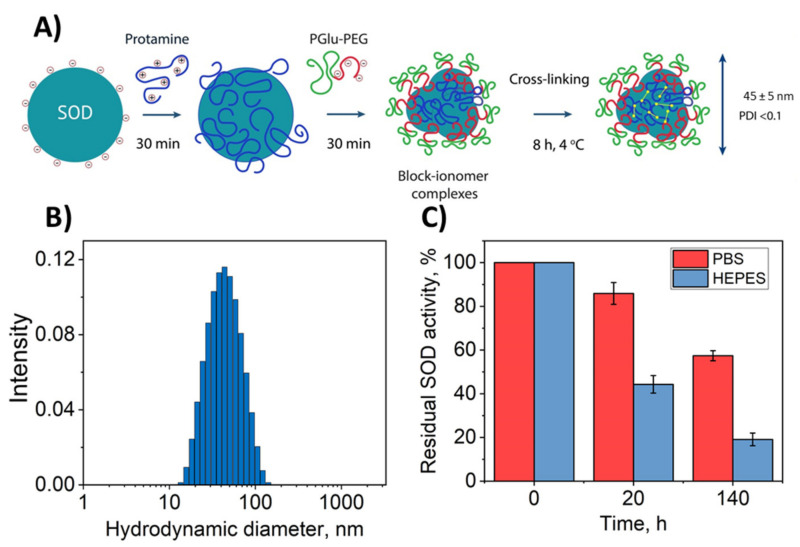
Physicochemical properties of Nano-SOD1 obtained. (**A**) Principal scheme for the synthesis of Nano-SOD1. (**B**) Particle hydrodynamic diameter distribution (SOD1/protamine/PGlu-PEG = 1:2:2 with glutaraldehyde (GA) as a cross-linker). (**C**) SOD1 release profile from a dialysis capsule with Nano-SOD1 obtained in HEPES and PBS buffers; 1 mL of Nano-SOD1 solution with specific activity of 13,500 U/mL was placed inside each dialysis capsule and 30 mL of PBS were placed outside the capsule.

Here, we evaluated a double-coated cross-linked polyion complex partly based on the approach described in Reference [41]. The principal scheme of the synthesis is presented in Figure 1A. Firstly, block–ionomer complexes were formed spontaneously as a result of electrostatic coupling of the enzyme and cationic polymer (protamine) and anionic block copolymer (PolyGlu-PLE). The Block–ionomer complex consists of several SOD1 globules based on the size of the resulting particles (Figure 1B), assuming the size of SOD1 molecule is less than 10 nm. Then, the cross-linker was added to complexes that resulted in covalent stabilization.

In the original method, such synthesis was carried out in HEPES buffer. It should be mentioned that HEPES is inappropriate in ophthalmology because HEPES can negatively affect the structures of the eye [50]. Thus, for comparison we obtained Nano-SOD1 in HEPES and PBS buffers.

It appeared that the replacement of the buffer did not significantly affect neither Nano-SOD1’s effective hydrodynamic diameter (Figure 1B), equal to 44–45 nm, nor ζ-potential equal to −7 mV (Table 1).

However, Nano-SOD1 synthesis in PBS helped to decrease the PDI value from 0.15 to <0.1 that resulted in a significantly narrowed size distribution of the particles. Moreover, the usage of PBS increased protein yield and residual enzyme activity (Table 1), statistical significance *p* < 0.05 being determined by the ANOVA test.

Additional advantage of the Nano-SOD1 synthesis in PBS was further demonstrated by the experiments on SOD1 release from nanoparticles. Despite covalent binding of SOD1 in conjugates, part of the enzyme molecules within nanoparticles was still in the form of complexes with polymers fixed by electrostatic interactions. The weaker the interactions, the stronger the equilibrium is shifted towards the release of SOD1 from nanoparticle. It was shown that the residual SOD1 activity in a dialysis capsule containing nanoparticles was significantly lower for Nano-SOD1 obtained in HEPES buffer compared to PBS, and the release of SOD1 into solution was much slower in the case of PBS (Figure 1C). This indicates that a larger number of SOD1 molecules in Nano-SOD1 are covalently bound to polymers or that electrostatic interactions of SOD1 with polymers are stronger in double-coated nanoparticles synthesized using our proposed technique in PBS. Thus, for further experiments, Nano-SOD1 was synthesized in PBS.

### 3.2. Stability of Nano-SOD1 Formulations

**Figure 2:**
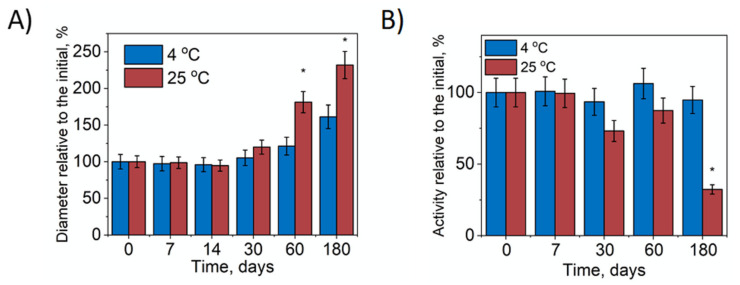
Stability of Nano-SOD1. (**A**) Relative change of Nano-SOD1’s hydrodynamic diameter during storage. (**B**) Relative change of SOD1 activity in Nano-SOD1 during storage. The PBS was used as a buffer solution. Data are mean ± SD (*n* = 3); * *p* ≤ 0.05 (ANOVA). Statistical significance is shown relative to parameters at the initial point.

An important characteristic of Nano-SOD1 formulation is storage stability. During Nano-SOD1 storage for 60 days at 4 °C, the hydrodynamic diameter of the particles remained practically unchanged, but as seen, after 180 days it increased 1.5 times (Figure 2A). However, SOD1 activity in Nano-SOD1 persisted for 180 days and then slightly decreased (Figure 2B). The stability of Nano-SOD1 obtained in PBS at 4 °C was comparable to that obtained in HEPES buffer [41]. When kept at r.t., the hydrodynamic diameter of Nano-SOD1 increased almost 1.75 times after 60 days of storage (Figure 2A). Longer storage resulted in a further increase in diameter with a sharp increase in PDI (up to 0.7). The increase in the size of the particles was accompanied by the loss of some SOD1 activity in the Nano-SOD1 formulation within 30 days of storage and a significant loss of activity after 180 days (Figure 2B).

Thus, the obtained Nano-SOD1 can be stored at 4 °C in a fridge for 180 days without significant changes to its properties.

### 3.3. Toxicity and Irritation Assays

It was found that the Nano-SOD1 formulations obtained in this work did not cause any eye irritation in rabbits at instillations, did not exhibit acute and chronic toxicity even at high doses, did not cause allergic reactions or affect reproductive ability of the animals, and did not possess neither mutagenic nor immunogenic properties at the doses used in this work. The data are presented in the Supplementary Materials (Tables S1–S94).

### 3.4. Dynamics of Nano-SOD1 in Tear and Intraocular Fluid

The major obstacles to the use of native SOD1 in ophthalmology are its rapid removal from mucous membrane of the eye surface and, as a result, the lack of needed enzymatic activity after passing the eye tissue barrier. We compared the behavior of native SOD1 labeled with Alexa 488 fluorescent dye and Nano-SOD1 based on such modified enzyme in biological fluids after their twice-repeated instillation into an eye. SOD1’s life in tears characterizes its ability to withstand the removal from this biological liquid likely due to the interaction with eye’s surface, while SOD1 in intraocular fluid characterizes drug penetration into an eye.

**Figure 3:**
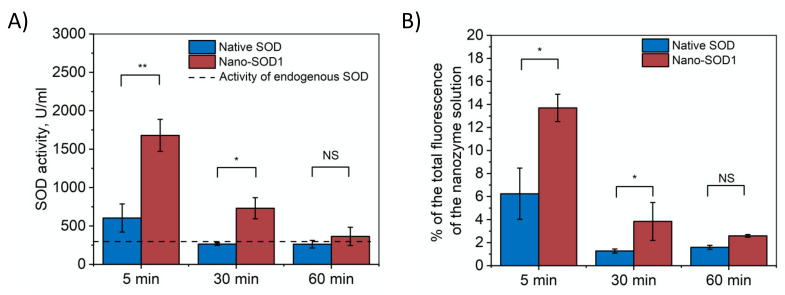
Dynamics of SOD1 and Nano-SOD1 in tears after instillation: (**A**) SOD1 activity; (**B**) SOD1 concentration (percentage of labeled SOD1 fluorescence relative to the initial protein fluorescence). The dashed line corresponds to the activity of endogenous SOD in tears. Symbols indicate the significance levels of differences according to the Mann–Whitney U-test: ** *p* < 0.01; * *p* < 0.05; NS—no difference.

It was shown that exogenous native SOD1 was washed out completely by the thirtieth minute after the last instillation (SOD1 level in a tear corresponded to the level of endogenous SOD1). Nano-SOD1, however, continued to function in the tear much longer (Figure 3A). As seen, its activity in the tear after 30 min was 2.5–3 times higher than the endogenous SOD1 level coming to that level only after an hour.

The dynamics of tear fluorescence that correlates with the amount of labeled SOD1 in tears (Figure 3B) corresponded to the measured SOD1 activity and, thus, confirmed that Nano-SOD1 stayed in the tear much longer than the native one.

The results, indeed, evidenced that Nano-SOD1 retains much better on the surface of eye cornea in comparison to native enzyme, and, thus, Nano-SOD1 could be considered as potential long-acting antioxidant drug.

**Figure 4:**
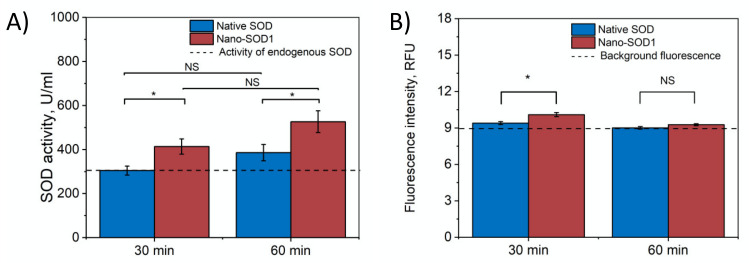
Dynamics of SOD1 and Nano-SOD1 in intraocular fluid after instillation: (**A**) SOD1 activity; (**B**) SOD1 concentration (fluorescence of labeled protein). The dashed lines correspond to the activity of endogenous SOD in tears. The symbols denote the significance levels of differences according to the Mann–Whitney U-test: * *p* < 0.05; NS—no difference.

Further, we evaluated the ability of Nano-SOD1 to penetrate into the anterior chamber of the eye. Figure 4A shows that in both cases, native SOD1 and Nano-SOD1, some enzyme passed through ocular barrier. However, Nano-SOD1 penetrated into the anterior chamber better than the native one. It is noteworthy that SOD1 activity in the intraocular fluid increased above the background level 30 min after the instillation of the Nano-SOD1, remaining increased even after an hour (Figure 4A). Labeled SOD1 fluorescence data also confirmed that Nano-SOD1 was able to penetrate the anterior chamber of the eye (Figure 4B).

It is noteworthy that while Nano-SOD1 activity was high in tears 30 min after instillation, its activity in the intraocular fluid was rather low. However, at 60 min after instillation, Nano-SOD1 activity in the intraocular fluid increased significantly, while activity in tears decreased (Figure 3 and Figure 4). This result indicates that Nano-SOD1 passes from the anterior surface of the eye into the inner region, this process definitely requiring time.

**Figure 5:**
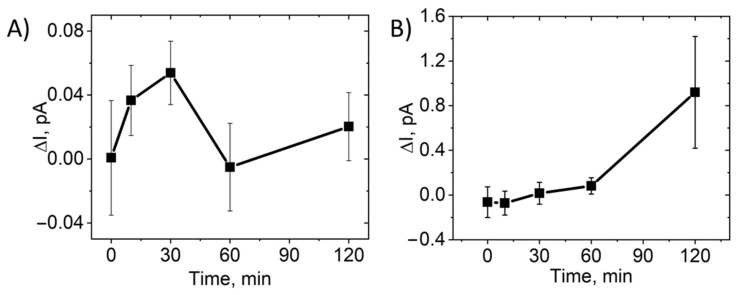
Dynamics of the H_2_O_2_ level in rabbit tears and intraocular fluid after a single instillation of Nano-SOD1. The data are presented as mean ± SEM for 5 rabbits. The current is proportional to hydrogen peroxide concentration. The H_2_O_2_ dynamics were calculated as the difference between the left (experimental) and right (control) eyes of each animal in tear (**A**) and intraocular fluid (**B**).

In addition, we determined the level of H_2_O_2_ in tear and intraocular liquid as an indicator of Nano-SOD1 functioning in these liquids. It should be mentioned that SOD1 catalyzes the dismutation of the superoxide radical into molecular oxygen and hydrogen peroxide. Therefore, after the instillation of Nano-SOD1, the level of hydrogen peroxide in animal’s tears increased, reaching a maximum at 30 min (Figure 5A). Apparently, this was due to a rather effective sorption of the Nano-SOD1 on the eye’s surface (see the dynamics of SOD1 activity and fluorescence, Figure 3), leading to an increase in Н_2_О_2_ concentration in 30 min. By 1 h, however, the Н_2_О_2_ concentration returned to its background level in accordance with the abovementioned observations (Figure 3) that at this point all Nano-SOD1 was washed away by a tear.

Unlike a tear, a noticeable increase in the level of hydrogen peroxide in the intraocular fluid was observed only 30 min after the instillation of Nano-SOD1 (Figure 5B). Obviously, it takes some time for Nano-SOD1 to penetrate into the anterior chamber of the eye. While SOD1 levels remained constant for an hour after the preparation instillation (Figure 4), the level of hydrogen peroxide in the intraocular fluid continuously increased for at least 2 h (Figure 5B). One can assume that this fact indicates continued functioning of Nano-SOD1 in the anterior eye chamber.

Thus, while the maximum SOD1 level in a tear was reached immediately after instillation of Nano-SOD1, it took time for the enzyme to enter the intraocular fluid.

### 3.5. Effects of Topical Instillations of Nano-SOD1 on Clinical Manifestations of Immunogenic Uveitis in Rabbits

At 24 h after intravitreal injection of equine serum, rabbits in all experimental groups (placebo-treated, native SOD1-treated, and Nano-SOD1-treated) showed typical clinical signs of acute uveitis: corneal and conjunctival edema, hyperemia of the iris, and fibrin precipitation on the iris surface. Over 3–4 days, these clinical signs intensified, but the development of uveitis in SOD1-treated and Nano-SOD1-treated groups remarkably differed from that in the placebo group.

Rabbits without any treatment (placebo) exhibited pronounced corneal and conjunctival edema and iris hyperemia. There was a lot of fibrin clots in the anterior part of the eye, which, in several cases, formed massive clouds.

The SOD1-treated rabbits showed less pronounced corneal and conjunctival edema, as well as iris hyperemia. However, there was no difference between the amount of fibrin clots observed in SOD1-treated and placebo-treated rabbits.

**Figure 6:**
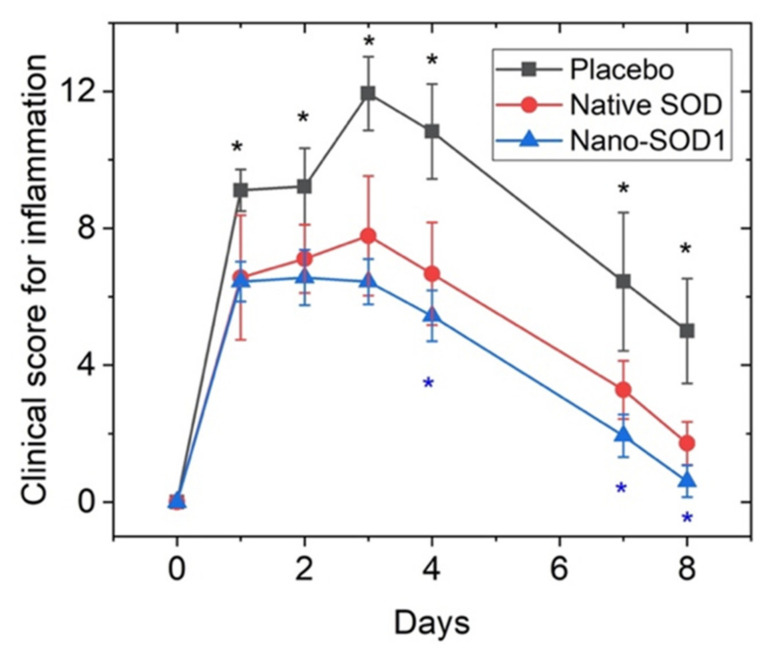
The effect of topical instillations of SOD1 and Nano-SOD1 on the development of inflammatory signs in rabbits with acute uveitis. Clinical scores for all signs of uveitis in each eye were summed up and expressed as mean ± SEM. * *p* < 0.05 (Mann–Whitney U-test).

In the group treated with Nano-SOD1, clinical manifestations of uveitis were even less pronounced than in placebo- and SOD1-treated groups (Figure 6). Most importantly, not only corneal and conjunctival edema and iris hyperemia were less pronounced than in two other groups, but we revealed a statistically different intensity of fibrin clots in the anterior chamber (*p* < 0.05 at 3, 4, and 7 days) between SOD1 and Nano-SOD1-treated groups.

Comparison of the effect of SOD1 and Nano-SOD1 on the total grade for intraocular inflammation (Figure 6) revealed statistically significant difference among the groups at 4, 7, and 8 days of the disease (*p* < 0.05).

Leukocyte counting in the intraocular fluid showed that both SOD1 and Nano-SOD1 had a remarkable influence on the inflammation in the anterior chamber of the eye. The average cell amount in both groups was significantly lower than that in the placebo group (*p* < 0.05). The leukocyte amount in the Nano-SOD1-treated group (5.6 pcs for five visual fields) was also less than in SOD1-treated group (4 pcs for five visual fields), but the difference was not statistically significant due to the high data variation.

**Figure 7:**
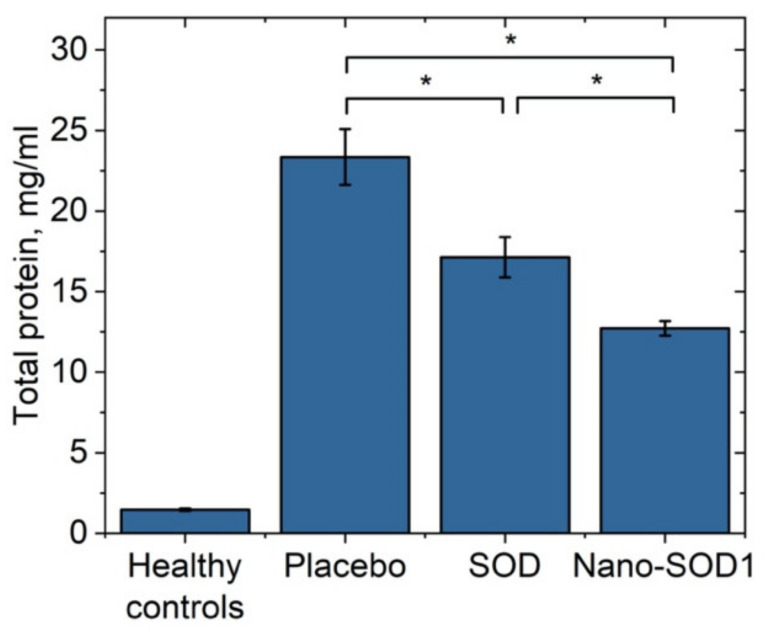
Total protein concentration in the intraocular fluid of rabbits on the 8th day of experimental immunogenic uveitis (mean ± SD) * *p* < 0.05 (Mann–Whitney U-test).

Analysis of intraocular fluid showed that uveitis was accompanied by a dramatic increase in the protein level (*p* < 0.01 for all groups) compared to the normal one of 1.06 ± 0.07 mg/mL, especially for the placebo group for which the protein level increased up to 23.4 ± 1.7 mg/mL. Both treated groups, however, showed significantly lower protein levels: 17.1 ± 1.3 mg/mL for SOD1-treated and 12.7 ± 0.5 mg/mL for Nano-SOD1-treated groups. So, treatment of animals with Nano-SOD1 decreased the protein level significantly, more effectively than the treatment with native SOD1 (*p* < 0.01) (Figure 7).

**Figure 8:**
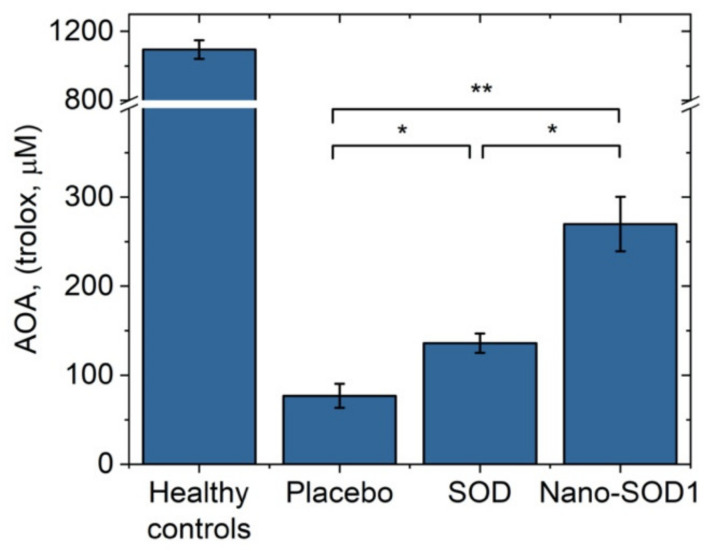
Antioxidant activity in the intraocular fluid of rabbits on the 8th day of experimental immunogenic uveitis (mean ± SD). ** *p* < 0.01; * *p* < 0.05 (Mann–Whitney U-test).

Uveitis was also accompanied by the exhaustion of endogenous antioxidant system of the eye. The AOA in the intraocular fluid on the 8th day of uveitis decreased drastically. In the placebo group, the AOA value was only 76 ± 13 µM Trolox, while the normal level of AOA was 1100 ± 54 µM Trolox. Treatment with SOD1 and Nano-SOD1, however, helped to maintain the AOA level, 136 ± 11 µM Trolox and 270 ± 30 µM Trolox for SOD1-treated and Nano-SOD1-treated groups, respectively. It is worth noting that Nano-SOD1 was significantly more effective than native enzyme (*p* < 0.01) in maintaining protective AOA (Figure 8).

## 4. Conclusions

The main results of the study are as follows: (i) A new formulation based on multilayer polyion SOD1 nanoparticles, Nano-SOD1, was specifically manufactured for local topical use in ophthalmology. Technique optimization allowed for more effective SOD1 interactions with polymers in nanoparticles and increased the residual SOD1 activity yield within Nano-SOD1 formulation; (ii) Nano-SOD1 storage stability studies showed that both the hydrodynamic diameter and SOD1 activity of the Nano-SOD1 formulation remained practically unchanged for at least 60 days at 4 °C; (iii) NanoSOD1’s ability to reduce inflammatory processes in the eye was demonstrated in vivo in rabbits with immunogenic uveitis representing the inner vascular eye tract inflammation. Topical Nano-SOD1 instillations showed high efficiency in decreasing uveitis manifestations, such as fibrin clots in the anterior chamber of the eye, corneal and conjunctival edema, and iris hyperemia. (iv) Nano-SOD1 did not exhibit any toxicity (eye irritation, acute, chronic and reproductive toxicity, allergenicity, immunogenicity, mutagenicity) in a wide concentration range and even at extremely high doses used; (v) compared to the native enzyme, Nano-SOD1 retains on the surface of eye’s cornea much better; it penetrates into interior eye structures more effectively and retains the enzyme activity in the eye for a much longer time; (vi) Nano-SOD1 not only decrease inflammation more effectively than the native enzyme, but also restore antioxidant potential of eye tissues in the model of immunogenic uveitis

Thus, the obtained Nano-SOD1 can be considered as a promising therapeutic agent for the treatment of eye diseases associated with the inflammatory processes.

## Figures and Tables

**Table 1:** Characteristics of Nano-SOD1 obtained in PBS and HEPES buffers.

Parameter	HEPES [41]	PBS
Diameter, nm	44 ± 2	45 ± 5
Polydispersity index (PDI)	0.15	<0.1
ζ-potential, mV	−7 ± 1	−7 ± 2
Protein yield,%	76 ± 8	89 ± 5
Residual enzyme activity yield,%	37 ± 6	42 ± 3

## Data Availability

Not applicable.

## Supplementary Materials

The following are available online at https://www.mdpi.com/article/10.3390/biomedicines9040396/s1.
