# Supplementary material for: Superoxide Dismutase 1 Nanoparticles (Nano-SOD1) as a Potential Drug for the Treatment of Inflammatory Eye Diseases
Source: Biomedicines. 2021 Apr 7;9(4):396. doi: 10.3390/biomedicines9040396 (PMC8067682; doi:10.3390/biomedicines9040396)
Supplement: Supplementary file 1 [file biomedicines-09-00396-s001.pdf]

Supplementary Materials

Superoxide Dismutase 1 Nanoparticles (Nano-SOD1) as Potential Drug for the Treatment of Inflammatory Eye Diseases

Alexander N. Vaneev [1,2], Olga A. Kost [1], Nikolay L. Eremeev [1], Olga V. Beznos [3], Anna V. Alova [1], Peter V. Gorelkin,[2] Alexander S. Erofeev [1,2], Natalia B. Chesnokova [3], Alexander V. Kabanov [1,4] and Natalia L. Klyachko [1,4,5]

[1] Lomonosov Moscow State University, Moscow 119991, Russia; vaneev.aleksandr@gmail.com; kost-o@mail.ru; eremeev@enzyme.chem.msu.ru; ava1945@mail.ru; Erofeev@polly.phys.msu.ru; kabanov@enzyme.chem.msu.ru; klyachko@enzyme.chem.msu.ru

[2] National University of Science and Technology "MISiS", Moscow 119049, Russia; peter.gorelkin@gmail.com

[3] Helmholtz National Medical Research Center of Eye Diseases, Moscow 101000, Russia; olval2011@mail.ru; nchesnokova2012@yandex.ru

[4] University of North Carolina at Chapel Hill, Chapel Hill, NC 27599, United States kabanov@enzyme.chem.msu.ru; klyachko@enzyme.chem.msu.ru

[5] G.R. Derzhavin Tambov State University, Tambov 392000, Russia klyachko@enzyme.chem.msu.ru

* Correspondence: klyachko@enzyme.chem.msu.ru

Toxicity studies

1. Animals 2
2. Acute toxicity 3
3. Chronic toxicity 12
4. Mutagenic properties of Nano-SOD1 63
5. Allergenic properties of Nano-SOD1 71
6. Immunotoxic properties of Nano-SOD1 76
7. Generative toxicity study 80
8. References 94

1. Animals

Toxicity studies were conducted using:

Adult male and female Shinshilla rabbits weighing 2.0-3.0 kg from laboratory animal nursery by the Federal State Unitary Enterprise Experimental production farm (Manihino branch) (acute and chronic toxicity);

Adult male and female rats of outbred line weighing 230-330 g from laboratory animal nursery FSBIS SCBMT FMBA of Russia “Andreevka branch” (acute, chronic and reproductive toxicity);

Adult male mice SVA line weighing 18-30 g from laboratory animal nursery FSBIS SCBMT FMBA of Russia “Andreevka branch” (allergenicity);

Adult male and female mice SVA line weighing 18-30 g from laboratory animal nursery FSBIS SCBMT FMBA of Russia “Andreevka branch” (immunotoxicity):

Adult male and female first generation hybrid mice (CBA × C57BL/6)F_1 line with the weight 25-30 g from laboratory animal nursery FSBIS SCBMT FMBA of Russia “Andreevka branch” (immunotoxicity, mutagenicity):

Adult male and female Himalayan white guinea pigs weighing 250-300 g from laboratory animal nursery FSBIS SCBMT FMBA of Russia “Andreevka branch” (allergenicity).

The selection of animals and the distribution of the groups were carried out by weight and age by random sampling. The body weight of each animal did not deviate from the group mean value by more than 20%.

Rats were kept in polycarbonate cages in groups of 5 heads, rabbits - in individual stainless steel cages. All cages were equipped with food and water supplies. A steel label holder was

attached to each cage, which indicated type, gender, quantity, start date of the study, study code, dose, etc. The animals received food without restriction.

In the nursery, constant environmental conditions were maintained: temperature in rat cages 18-22 ° C, in rabbit cages - 15-18 ° C, 50-65% relative humidity, 12-hour lighting cycle and 8-10-fold change in room air volume in hour. Temperature and humidity were recorded and documented once a day.

All experiments with live animals were carried out in accordance with the Association for Research in Vision and Ophthalmology (ARVO) statement for the Use of Animals in Ophthalmic and Vision Research and in accordance with European Convention for the Protection of Vertebrate Animals Used for Experimental and other Scientific Purposes (ETS 123) Strasbourg, 1986.

2. *Acute toxicity*

Materials and methods

The experiment was simulated in accordance with the requirements for determining the acute toxicity of the compound[1,2]. The experimental scheme for the study of acute toxicity is shown in Table S1.

Rats. When intravenously administered, Nano-SOD1 was injected in the lateral vein of the rats tail using 27G syringes in the maximum possible volume (2 ml), which amounted to an average dose of 7 ml/kg or 91,000 units/kg (average rat weight 280 g) and exceeded the recommended dose for humans (89 units of activity by pyrogallol/day) by more than 1000 times. Upon instillation, Nano-SOD1 was instilled with one drop (0.052 ml) in each eye 24 times with an interval between instillations of 15 minutes (total 2.5 ml), which amounted to an average dose (average rat body weight 270 g) of 240,000 units/kg and exceeded the recommended dose for humans by 2700 times. In each case, similar in number groups of control animals of each sex were formed, by which the PBS buffer (10 mM sodium phosphate buffer, pH 7.4, containing 150 mM NaCl) was injected (or instilled) in the same way and in the same volumes. Animals were removed from the experiment on day 15 by decapitation with preliminary anesthesia. Experimental design of acute toxicity study on rats is shown in Table S2.

Rabbits. Upon instillation, Nano-SOD1 was instilled with two drops (0.104 ml) in each eye 24 times with an interval between instillations of 15 minutes (total 5 ml), which amounted to an average dose (average rabbit body weight of 2.5 kg) of 26,000 units/kg and exceeded the recommended dose for humans nearly 300 times. In each case, similar in number groups of control animals of each sex were formed, by which the solvent PBS (10 mM sodium phosphate

buffer, pH 7.4, containing 150 mM NaCl) was instilled in the same way and in the same volumes.

Since rabbits did not show signs of intoxication during the experiment, and in an acute experiment on rats with autopsy after repeated instillations into the conjunctival sac of the eye, Nano-SOD1 did not reveal a pathological picture of rat internal organs during macroscopic examination and morphometry, rabbits were not euthanized. Experimental design of acute toxicity study on rabbits is shown in Table S3.

Table S1. The scheme of the experiment in the study of acute toxicity.

Indicators	Days of experiment							
	-1	1	2	3	4-7	8	9-14	15
Grouping and animal identification	■							
Weighing	■			■		■		■
Drug administration		■						
Observation	■	■	■	■	■	■	■	■
Animal Euthanasia								■

Table S2. Design of acute toxicity study on rats.

Groups	Route of administration	Group №	Animal sex*	The number of animals in the group	The average dose, ml/kg	Euthanasia day
Control (PBS)	intravenous	1	♂	5	---	15
		2	♀	5		
	instillation	3	♂	5	---	
		4	♀	5		
Nano-SOD1	intravenous	5	♂	5	7	
		6	♀	5		
	instillation	7	♂	5	9	
		8	♀	5		

* ♂ - male, ♀ - female.

Table S3. Design of acute toxicity study on rabbits.

Groups	Route of administration	Group №	Animal sex*	The number of animals in the group	The average dose, ml/kg
Control (PBS)	instillation	1	♂	4	---
		2	♀	4	
Nano-SOD1		3	♂	4	2
		4	♀	4	

* ♂ - male, ♀ - female.

Observations and measurements during the study

On the day of administration of the test substances, the animals were observed every hour, on the following days - 2 times a day (morning and evening). The assessment of the toxic effect of the drug in an acute experiment was carried out according to the following clinical signs:

• the number and timing of death of animals (if any);

• respiratory indicators (shortness of breath, cyanosis, rapid breathing, nasal discharge);

• physical activity (increased/decreased, drowsiness, loss of balance, sensitivity, catalepsy, ataxia, unusual movements, prostration, tremor, fasciculation);

• convulsions (clonic, tonic, tonic-clonic, asphytic);

• reflexes (corneal, balance, myotatic, light, startle reflex);

• cardiovascular events (bradycardia, tachycardia, arrhythmia);

• increased salivation;

• condition of the coat (piloleiomyoma, alopecia);

• analgesia;

• muscle tone (hypotension, hypertension);

• gastrointestinal indicators (soft stools, diarrhea, vomiting, polyuria, rhinorrhea);

• zoosocial behavior;

• eye signs (lacrimation, miosis, mydriasis, exophthalmos, ptosis, opacity, iritis, conjunctivitis, chromodactriorrhea, weakening of the blinking membrane).

Body weight of each animal was determined before the study, on the 3rd, 8th and 15th days of the experiment on an ACOM PC-100W-5 scale (ACOM, South Korea). The accuracy of the weights used was verified prior to the start of the study.

Terminal procedures

To assess the damage effect of Nano-SOD1 on internal organs, all rats on the 15th day of the experiment were euthanized by decapitation with preliminary anesthesia. Pathological examination included autopsy, gross examination and weighing of internal organs. After euthanasia, the animals were carefully examined for external pathological signs: general condition; the condition of the mucous membranes of the eyes, the condition of the natural openings; the presence and nature of the discharge; condition of the hairline. A study of the state of the chest and abdominal cavity, the state of the brain and the place of administration of the drug (eyes), macroscopic examination of internal organs were carried out.

The list of organs subject to macroscopic examination: skin, lymph nodes, aorta, heart, larynx, trachea, lungs, thymus, esophagus, stomach, small intestine, large intestine, liver, pancreas, spleen, kidneys, adrenal glands, bladder, testes, ovaries, uterus, submandibular salivary gland with lymph nodes, thyroid gland, brain, injection site (tail vein, eyes).

List of organs to be weighed: heart, lungs, thymus, liver, spleen, kidneys, testes, ovaries, adrenal glands, brain. The morphometric assessment of the parameters of animal organs was carried out using a Shinko, AJ-1200CE balance (Shinko Denshi Co., Ltd, Japan).

Data processing

Statistical processing of the data obtained was carried out using the Microsoft Office Excel 2010 software. The group arithmetic mean and standard error of the mean (SE) were calculated. The statistical significance of the differences was assessed by the Student's t-test. The difference between these groups was considered significant at $p < 0.05$.

Results of acute toxicity study on rats

In this experiment, rats (male and female) were used. Distribution into groups was carried out by random sampling. Body weight was taken as a criterion. Each group of rats included 5 animals. Two route of administration (intravenous and instillation), two doses were used in the experiment. In addition, groups of control animals of each sex, similar in size, were formed, which were injected in the same way and in the same volumes with a buffer- PBS (10 mM sodium phosphate buffer, pH 7.4, containing 150 mM NaCl).

Observing animals

Survival. During the experiment, no deaths were recorded.

Appearance and behavior. In all groups, the animals looked healthy, willingly ate food, reacted to external stimuli, and showed interest in people. Muscle tone was not characterized by increased excitability. The rats were of average fatness, did not suffer from exhaustion. The coat

is dense, even and shiny, tightly adhered to the surface of the body, no hair loss or brittleness was found. The abdominal area is not enlarged. Breathing is even, of the usual rhythm, not difficult. Salivation was without pathology. The auricles were pink in color without crusts, not inflamed, twitching is not noticed. The teeth were of normal color, no breakages were observed. The frequency of urination, urine color, gastrointestinal parameters, muscle tone, reflexes corresponded to the physiological norm. The zoosocial behavior of the experimental rats did not differ from the control animals.

Eye examination. The rats showed no negative response to eye instillation. According to the external examination:

• the cornea was smooth, shiny, without clouding;

• pink conjunctiva, no hyperemia, non-edematous, no discharge

• the pupil was round, reacts to light.

The results of weighing animals are shown in Tables S4, S5. The body weight of the rats during the experiment had a positive trend and did not differ significantly between the experimental and control groups.

Table S4. Dynamics of body weight of rats after intravenous injection of Nano-SOD1. Data are mean ± SE, g

Days	Groups			
	♂		♀	
	Group 1 (Control)	Group 5 (Nano-SOD)	Group 2 (Control)	Group 6 (Nano-SOD)
0	321.5±15.83	317.6±10.57	238.0±5.43	237.6±5.21
3	352.4±18.94	346.6±13.11	246.2±5.05	246.1±6.08
8	355.6±19.29	351.6±11.90	259.0±7.08	259.0±8.03
15	365.8±20.85	351.9±13.53	266.7±4.70	269.1±8.38

Table S5. Dynamics of body weight of rats during instillation into the conjunctival sac of the eye. Data are mean ± SE, g

<table>
<tr><td rowspan="3">Days</td><td colspan="4">Groups</td></tr>
<tr><td colspan="2">♂</td><td colspan="2">♀</td></tr>
<tr><td>Group 3 (Control)</td><td>Group 7 (Nano-SOD)</td><td>Group 4 (Control)</td><td>Group 8 (Nano-SOD)</td></tr>
<tr><td>0</td><td>320.5±19.25</td><td>304.6±5.68</td><td>232.7±2.66</td><td>238.4±6.17</td></tr>
<tr><td>3</td><td>326.8±18.80</td><td>330.6±7.80</td><td>245.5±8.47</td><td>245.7±8.14</td></tr>
<tr><td>8</td><td>333.6±18.07</td><td>334.5±8.00</td><td>253.1±11.13</td><td>250.3±7.71</td></tr>
<tr><td>15</td><td>347.1±13.45</td><td>345.2±7.14</td><td>270.7±9.32</td><td>261.4±8.46</td></tr>
</table>

Pathomorphological data

Macroscopic examination

The animals were taken out of the experiment on the 15th day. A macroscopic study of a clear effect of Nano-SOD1 on the state of the internal organs of rats was not found, differences between the control and experimental groups were not found, and therefore, the study data are presented in one section.

The location of the internal organs was correct. No free fluid was found in the pleural and abdominal cavities. The lumen of the trachea and bronchi was free, their mucous membrane was clean, moist, shiny.

The intima of the aorta was smooth, shiny, whitish in color. The diameter of the aorta was not changed.

The heart was of the correct shape, not enlarged, the myocardium of an elastic consistency, moist, and shiny, the fiber pattern was well expressed. Large vessels lying in the pericardial region were well expressed. In the cavity of the heart bag there were several drops of a transparent straw-yellow liquid. The valves of the heart were thin, smooth, and shiny.

The larynx was represented by movably connecting cartilages; the inside was lined with a mucous membrane that forms a series of folds and ventricles.

Light, moderately collapsed, had a smooth soft surface.

The spleen was not enlarged, elongated, the surface was smooth, the consistency was dense, the pulp was not scraped off, the follicles and trabeculae looked like gray dots and stripes on the cut.

The liver was of the correct shape, not enlarged, of dense consistency, smooth and shiny. The capsule was thin, transparent.

The kidneys were of the correct bean-shaped form, the fibrous capsule was easily detached. The section clearly showed the border of the cortical and medullary layers.

The adrenal glands were rounded, of moderately dense consistency. The medulla was clearly visible on the cut.

Bladder. The mucous membrane of the bladder was smooth, shiny.

The mucous membrane of the esophagus was shiny, smooth, pale in color.

Stomach without swelling, normal shape and size. Hyperemia, erosions, hemorrhages were not observed.

The pancreas was unchanged, lobular.

Intestines without swelling and adhesions. The mucous membrane was shiny, smooth.

Testes were normal.

The vaginal mucosa was smooth, shiny, collected in distinct longitudinal vaginal folds.

The ovaries had an uneven granular surface and a rounded shape.

The body of the uterus was moderately dense, of normal size.

The thymus was triangular in shape, of moderately dense consistency, without hemorrhages.

Submandibular lymph nodes were oval, soft, with a thin capsule.

The thyroid gland was dense with symmetrical lobes, firmly attached to the larynx.

The salivary glands had a round or oval shape, a smooth surface.

The membranes of the brain were moderately filled with blood, moist, shiny. The medulla had a symmetrical cut pattern, of moderate density. The expansion of the ventricles of the brain was not observed.

Morphometric analysis of internal organs

The relative masses of animal organs are presented in Tables S6, S7 After repeated instillations, Nano-SOD1 did not reveal any abnormalities in the mass of the internal organs of rats.

Table S6. Average group measures of the relative mass of the internal organs of rats after intravenous administration of Nano-SOD1, %.

Organs	Groups			
	♂		♀	
	Group 1 (Control)	Group 5 (Nano-SOD)	Group 2 (Control)	Group 6 (Nano-SOD)
Liver	2.67±0.096	2.94±0.039	3.16±0.120	3.50±0.045
Kidney	0.61±0.013	0.67±0.023	0.57±0.014	0.61±0.016
Spleen	0.26±0.007	0.27±0.021	0.23±0.026	0.32±0.031
Heart	0.29±0.011	0.31±0.010	0.30±0.007	0.31±0.009
Thymus	0.15±0.017	0.14±0.014	0.09±0.002	0.12±0.010
Gonads	0.84±0.028	0.90±0.034	0.040±0.0040	0.043±0.0052
Lungs	0.52±0.048	0.51±0.041	0.52±0.042	0.53±0.021
Adrenal glands	0.016±0.001	0.015±0.002	0.026±0.002	0.023±0.001
Brain	0.54±0.019	0.54±0.029	0.66±0.032	0.69±0.020

Table S7. Average group measures of the relative mass of the internal organs of rats after instillation of Nano-SOD1 into the conjunctival sac of the eye, %.

Organs	Groups			
	♂		♀	
	Group 3 (Control)	Group 7 (Nano-SOD)	Group 4 (Control)	Group 8 (Nano-SOD)
Liver	2.56±0.081	2.87±0.148	3.03±0.233	3.19±0.045
Kidney	0.59±0.017	0.63±0.030	0.58±0.022	0.56±0.010
Spleen	0.28±0.16	0.29±0.026	0.32±0.032	0.24±0.012
Heart	0.28±0.008	0.29±0.013	0.32±0.018	0.40±0.016
Thymus	0.12±0.012	0.13±0.014	0.13±0.009	0.13±0.026
Gonads	0.84±0.036	0.87±0.018	0.048±0.0050	0.041±0.0030
Lungs	0.47±0.040	0.48±0.016	0.56±0.060	0.54±0.035
Adrenal glands	0.019±0.001	0.018±0.002	0.024±0.001	0.023±0.002
Brain	0.49±0.012	0.51±0.023	0.62±0.015	0.65±0.033

Results of acute toxicity study on rabbits

In this experiment, (male and female) were used. Distribution into groups was carried out by random sampling. Body weight was taken as a criterion. Each group of rabbits included 4 animals. Two route of administration (intravenous and instillation), one dose were used in the experiment. In addition, groups of control animals of each sex, similar in size, were formed, which were injected in the same way and in the same volumes with a buffer- PBS (10 mM sodium phosphate buffer, pH 7.4, 150 mM NaCl).

Observing animals

Survival. During the experiment, no deaths were recorded.

Appearance and behavior. In all groups, the animals looked healthy, willingly ate food, reacted to external stimuli, and showed interest in people. The rabbits were of average fatness, did not suffer from exhaustion. The coat was thick, even and shiny, no hair loss was found. The abdominal area was not enlarged. Breathing was even, of the usual rhythm, not difficult. Salivation was without pathology. Auricles without crusts, not inflamed, twitching was not noticed. The teeth were of normal color, no breakages were observed. The frequency of urination, urine color, gastrointestinal parameters, muscle tone, reflexes corresponded to the physiological norm.

Eye examination. Rabbits showed no negative response to eye instillation. According to the external examination:

- the cornea was smooth, shiny, without clouding;
- pink conjunctiva, no hyperemia, non-edematous, no discharge
- the iris was evenly pigmented;
- the pupil was round, reacted to light.

Dynamics of body mass

The results of weighing the animals are shown in Table S8. The body weight of rabbits during the experiment had a positive trend and did not differ significantly between the experimental and control groups.

Since the rabbits during the experiment did not show signs of intoxication, and in an acute experiment on rats during autopsy after repeated instillations of Nano-SOD1 into the conjunctival sac of the eye, no pathological picture of internal organs was revealed during macroscopic examination and morphometry, there was no need to euthanize rabbits.

Table S8. Dynamics of the body weight of rabbits upon instillation of Nano-SOD1 into the conjunctival sac of the eye. Data are mean ± SE, g

Days	Groups			
	♂		♀	
	Group 1	Group 3	Group 2	Group 4
0	2965±93.1	2906±130.62	2090±70.2	2104±48.0
3	3149±98.6	3012±109.9	2202±55.5	2215±35.3
8	3305±87.3	3164±44.9	2339±33.5	2303±49.8
15	3426±87.9	3348±126.9	2516±27.4	2500±34.2

Thus, an experimental study of acute toxicity in sexually mature outbred rats and rabbits of both sexes, observation data on experimental animals, as well as autopsy data indicate the absence of an acute toxic effect of Nano-SOD1 on the animal organism.

3. *Chronic toxicity*

In this experiment, rats and rabbits (male and female) were used. Distribution into groups was carried out by random sampling. Body weight was taken as a criterion. Each group of rats included 15 animals and each group of rabbits included 4 animals. Total of 6 groups of rabbits and 6 group of rats (2 control + 4 experimental). One route of administration (instillation), two doses were used in the experiment. In addition, groups of control animals of each sex, similar in size, were formed, which were injected in the same way and in the same volumes with a buffer-PBS (10 mM sodium phosphate buffer, pH 7.4, 150 mM NaCl).

Rats. Nano-SOD1 was instilled one drop (0.052 ml) in each eye once a day in one group of animals (0.13 ml/kg) and 1 drop in each eye 2 times a day in other groups (0.53 ml/kg). These doses for rats averaged 0.13 ml/kg and 0.53 ml/kg. In terms of doses from rat to person (coefficient 5.9), these doses will be 0.02 ml/kg and 0.09 ml/kg, which exceeds the recommended daily doses for humans (0.003 ml/kg) by 6.7 and 30 times respectively. In each case, similar in number groups of control animals of each sex were formed, by which the PBS buffer (10 mM sodium phosphate buffer, pH 7.4, containing 150 mM NaCl) was instilled in the same way and in the same volumes. Animals were removed from the experiment on day 15 by decapitation with preliminary anesthesia (Table S9).

Rabbits. Nano-SOD1 was instilled one drop in each eye once a day and 2 drops in each eye 2 times a day. In terms of doses from rabbit to person (conversion factor 3.2), these doses will be 0.04 ml/kg and 0.15 ml/kg, which exceeds the recommended daily doses for humans (0.003 ml/kg) by 3.3 and 16.7 times, respectively. In each case, similar in number groups of control animals of each sex were formed, by which the solvent PBS (10 mM sodium phosphate buffer,

pH 7.4, containing 150 mM NaCl) was instilled in the same way and in the same volumes (Table S10).

Nano-SOD1 and PBS buffer (control groups) were administered to the animals for 4 weeks. A day after the last injection, 10 rats from each group and all rabbits were removed from the experiment with the study of integral and physiological parameters, blood tests and pathological examination. The other part of the rats was followed for another 2 weeks. After observation, they were euthanized with examination in the same volume as the animals killed immediately after the end of the administration of the test agent (Table S9-S11). The total observation period for rats is 43 days, for rabbits - 29 days.

Table S9. Design of chronic toxicity study on rats.

Groups	Animals	№ group	Animal sex*	The number of animals in the group	The average dose, ml/kg	Euthanasia day
Control	Rats	1	♂	15	0.53	10 animals on the 29th day of the experiment, the rest on the 43rd day
		2	♀	15		
Nano-SOD		3	♂	15	0.13	
		4	♀	15		
		5	♂	15	0.53	
		6	♀	15		

Table S10. Design of chronic toxicity study on rabbits.

Groups	Animals	№ group	Animal sex*	The number of animals in the group	The average dose, ml/kg	Euthanasia day
Control	Rabbits	1	♂	4	0.15	29th day of the experiment
		2	♀	4		
Nano-SOD		3	♂	4	0.04	
		4	♀	4		
		5	♂	4	0.15	
		6	♀	4		

Table S11. Experimental scheme for the study of chronic toxicity.

Indicators	Days																
	0	1-7	8	9-14	15	16-21	22	23-26	27	28	29	30-34	35	36-40	41	42	43
Weighing													*				*
Drug administration																	
Observation												*	*	*	*	*	*
Metering of feed and water consumption													*		*		
Thermometry															*		
Assessment of behavior in the test "open field" *															*		
Removal of ECG parameters																*	
Breathing rate																*	
Clinical blood test																	*
Indicators of hemostasis																	*
Blood chemistry																	*
Analysis of urine															*		
Intraocular pressure**																	
Biomicroscopic examination **																	
Fundus examination **																	
Euthanasia of animals										№							*

* -only for rats

** - only for rabbits

№-part of rats and all rabbits

Observations and measurements during the study

Registration of symptoms of intoxication

Each animal was monitored daily during the study. Examination included: survival, appearance, condition of the coat, eyes, nose, breathing pattern, behavior (excitability, aggressiveness), reaction to external stimuli, pain reaction, food and water consumption, quantity and consistency of fecal matter, frequency of urination, and urine color.

Body mass

Body weight of each animal was determined by weighing before drug administration, then weekly. The animals were weighed on an ACOM PC-100W-5 scale (ACOM, South Korea). The accuracy of the weights used was verified prior to the start of the study.

Feed and water consumption

Before the study and then, once a week, the consumption of food and water was taken into account. For this, the food was weighed before distribution and after 24 hours, the volume of water was fixed in drinking bottles every other day. To determine the average amount of food and water consumed per 1 rat (kept in groups), the group value was divided by the number of rats in the cage.

Temperature measurement

The temperature was measured rectally using an electronic medical thermometer (B.WELLWT-03 base, Germany).

Indications for cardiovascular activity

Measurement of heart rate and registration of ECG was carried out using the device Poly-Spectrum-8/V for veterinary medicine (LLC "Neurosoft", Russia, information on the verification of AA 3183933/06427 until 27.10.17.).

Respiratory system

The measurement of the frequency of respiratory movements was carried out in animals in a resting state for 1 minute.

Clinical blood test

Blood samples for clinical analysis were taken from the tail vein in rats and from the lateral ear vein in rabbits after 18 hours of fasting. Blood was collected in special tubes "Univet" with EDTA anticoagulant. Analyzes were carried out on a Medonic CA-620 "LOKE" hematological analyzer for veterinary medicine (Boule Medical AB, Sweden). The following indicators were determined:

- red blood cell count (RBC)
- hematocrit (HCT)
- platelet count (PLT)
- the amount of hemoglobin (HGB)
- the number of leukocytes (WBC) with differentiation in 3 populations: lymphocytes (LYM), monocytes (MID), granulocytes (GRAN)

The studies were carried out before the administration of the drug, 1 day after the last administration of the drug, and at the end of the experiment (on the 43rd day in the "set aside" groups of rats).

Indicators of hemostasis

Blood was collected in tubes containing 3.8% sodium citrate solution and centrifuged to obtain plasma. Hemostasis indices were recorded using an APG2-02-P two-channel analyzer

(ZAO NPP Tekhnomedika) using kits for coagulological tests produced by NPO RENAM. The following indicators were determined:

- fibrinogen content
- prothrombin time
- activated partial thromboplastin time (APTT).

Blood chemistry

Blood sampling was performed in rats during decapitation, in rabbits during their lifetime from the marginal ear vein after 18 hours of fasting. Blood was collected in special tubes with granules for serum separation. Biochemical analyzes were performed using standardized methods on a Stat fax 4500+ biochemical photometer (Awareness Technology Inc., USA) using standard reagent kits from Unimed and Olvex Diagnosticum. The following indicators were determined:

- aspartate aminotransferase
- alanine aminotransferase
- alkaline phosphatase
- total protein
- urea
- creatinine
- glucose
- cholesterol
- triglycerides
- total bilirubin

Biochemical analysis of urine

Urine analyzes were performed on a URISCAN optima analyzer (YD Diagnostics, Korea) using Uriscan strips. The following indicators were determined:

- leukocytes
- nitrites
- pH
- protein
- glucose
- urobilinogen
- bilirubin
- ketones
- erythrocytes
- ascorbic acid

- specific gravity

Behavioral responses in the open field test (rats only)

The "open field" experimental setup is a 100x100x60 cm chamber with a square floor. The chamber floor was divided into 16 squares. From above, the chamber was illuminated by a 100-watt electric incandescent lamp located at a height of 1 m from the floor of the chamber. The rats were placed one at a time in the middle of the chamber, and then for 3 min. registered:

- adaptation time (latency period)
- number of crossed horizontal squares (horizontal activity)
- getting up on the hind legs (vertical activity)
- washing (grooming)
- acts of defecation by the number of fecal balls (boluses)
- the number of urinations.

Ophthalmic indicators

External examination

Every day before each instillation and 10 minutes after it, the eyes of the rabbits were examined for abnormalities.

Puppilometry (for rabbits only)

Before the administration of the drug and 1 day after the last administration, the pupil size was recorded in 2 projections: horizontal and vertical.

Biomicroscopy of the eye (only for rabbits)

Biomicroscopy of the eyes was performed using a ShchL-2B slit lamp (TochMedPribor, Russia). Before biomicroscopy, the cornea was stained with a 0.5% fluorescein solution, which makes it possible to reveal the smallest signs of keratopathy in case of its occurrence. Evaluated:

- transparency of optical media
- condition of the cornea
- the state of the anterior chamber

Measurement of intraocular pressure (only for rabbits)

The measurement of intraocular pressure was carried out using metal weights to determine the intraocular pressure according to Maklakov NGm2- "OFT-P" (IP Gurov A.S.)

The measurement was carried out before drug administration and 1 day after the last administration.

Ophthalmoscopy of the eye (for rabbits only)

Eye ophthalmoscopy was performed using a Heine beta 200 hand ophthalmoscope (Heine, Germany). Evaluated:

- optic disc

- vessels of the eye
- retina

The study was carried out before drug administration and 1 day after the last administration.

Terminal procedures

On the 29th (part of the rats and all rabbits) and 43rd day (the "set aside" groups of rats) of the experiment, the animals were euthanized (rats by decapitation with preliminary anesthesia, rabbits - by air embolism with preliminary anesthesia) with further macroscopic, morphometric and histological examination of organs and fabrics.

List of organs subject to macroscopic examination: site of injection, skin, lymph nodes, aorta, heart, larynx, trachea, lungs, thymus, esophagus, stomach, small intestine, large intestine, liver, pancreas, spleen, kidneys, adrenal glands, bladder , testes, ovaries, uterus, submandibular salivary gland with lymph nodes, thyroid gland, brain.

List of organs to be weighed: heart, lungs, thymus, liver, spleen, kidneys, testes, ovaries, adrenal glands, brain. The morphometric assessment of the parameters of animal organs was carried out using a Shinko scale, AJ-1200CE (Shinko Denshi Co., Ltd, Japan).

The list of organs subject to histological examination: aorta, heart, lung, thymus, liver, spleen, stomach, esophagus, intestines, pancreas, kidney, adrenal gland, testis, ovary, brain, injection site (cornea of the eye).

Organs and tissues were fixed in 10–15% neutral formalin and embedded in paraffin. Slices 5–6 μm thick were prepared from paraffin blocks using an RMD-4000 rotary automatic microtome (Mt Point, Russia). Sections were stained with hematoxylin-eosin.

Histological preparations were examined by light microscopy using a Leica DM1000 biological microscope (Leica Microsystems CSC GmbH, Germany).

Results of a chronic toxicity study in rats

Clinical observations

Survival rate. During the study, all the animals remained alive.

Appearance and behavior. In all groups, rats actively ate food, reacted to external stimuli, and showed interest in people. Muscle tone was not characterized by increased excitability. The rats were of average fatness and did not suffer from exhaustion. The coat was smooth and shiny; no hair loss or breakage has been detected. No corneal opacities, lacrimation, or any eye abnormalities were observed. The auricles were pink without crusts, not inflamed, twitching was not noticed. Teeth of normal color, no breakdowns were observed. Respiration in animals was

normal, uncomplicated; salivation without pathology; frequency, quantity and consistency of fecal masses within the physiological norm.

During instillation, the animals did not show any anxiety, vocalization was absent. Examination of the eyes revealed no hyperemia, edema, or discharge.

The results of weighing the rats are shown in Tables S12 and S13. The body weight of rats did not significantly differ between the control and experimental groups throughout the study.

Table S 12. Dynamics of body weight in male rats. Data are mean ± SE, g.

Days	Groups		
	Group 1, Control, PBS	Group 3, Nano-SOD1 0.13 ml/kg	Group 5, Nano-SOD1 0.53 ml/kg
0	343.6±7.62	357.0±9.56	355.0±7.87
8	359.7±7.57	369.9±8.30	367.3±8.39
15	388.0±7.44	389.7±8.93	392.3±8.81
22	395.7±7.20	392.6±7.44	395.9±8.65
29	399.2±7.29	395.4±8.26	398.4±9.35
36	419.1±14.69	417.2±20.27	417.8±14.07
43	409.1±12.19	398.0±22.82	410.6±12.62

Table S13. Dynamics of body weight in female rats. Data are mean ± SE, g.

Days	Groups		
	Group 2, Control, PBS	Group 4, Nano-SOD1 0.13 ml/kg	Group 6, Nano-SOD1 0.53 ml/kg
0	275.0±5.09	275.3±6.49	277.9±6.66
8	279.9±4.85	279.3±6.08	280.7±6.12
15	288.2±4.68	284.4±5.78	286.1±6.07
22	294.9±5.56	289.8±5.94	289.4±5.45
29	297.4±5.43	285.4±5.94	283.6±6.24
36	298.4±10.93	293.1±6.30	297.9±13.72
43	282.7±12.37	283.7±6.33	279.5±12.71

Feed and water consumption

Feed and water consumption were taken into account once a week. The data are shown in Tables S14-S17. As can be seen from the presented results, the amount of food consumed and water consumed did not differ between the groups.

Table S14. Male rat food intake (g).

Days	Groups		
	Group 1. Control. PBS	Group 3. Nano-SOD1 0.13 ml/kg	Group 5. Nano-SOD1 0.53 ml/kg
0	31.4	31.2	30.9
8	31.7	30.6	30.6
15	30.5	31.8	31.3
22	31.5	30.4	31.6
28	31.7	30.3	30.9
36	32.6	31.3	32.0
43	34.2	29.2	33.6

Table S15. Female rat food intake (g).

Days	Groups		
	Group 2. Control. PBS	Group 4. Nano-SOD1 0.13 ml/kg	Group 6. Nano-SOD1 0.53 ml/kg
0	23.0	23.5	23.2
8	26.7	23.7	22.7
15	23.5	24.3	23.7
22	23.9	23.1	22.7
28	23.1	23.6	23.1
36	23.8	24.6	23.6
43	23.4	23.6	22.4

Table S16. Water consumption by male rats (ml).

Days	Groups		
	Group 1. Control. PBS	Group 3. Nano-SOD1 0.13 ml/kg	Group 5. Nano-SOD1 0.53 ml/kg
0	51.7	49.0	49.1
8	51.2	42.7	42.4
15	40.6	38.8	39.7
22	47.1	45.4	44.4
28	49.0	47.0	48.1
36	47.6	48.2	46.4
43	48.4	47.0	49.4

Table S17. Water consumption by female rats (ml).

Days	Groups		
	Group 2. Control. PBS	Group 4. Nano-SOD1 0.13 ml/kg	Group 6. Nano-SOD1 0.53 ml/kg
0	39.3	41.1	40.5
8	36.7	34.0	34.3
15	30.9	31.7	32.1
22	40.5	40.9	40.3
28	41.9	40.8	40.7
36	42.4	41.6	42.2
41	40.6	42.6	41.0

Rectal temperature measurements

Data for measuring the rectal temperature of rats are presented in Tables S18 and S19. The temperature readings of the animals during the study were within the physiological norm and did not differ between the groups.

Table S18. Indicators of rectal temperature in male rats (^{o}C, Mean ± SE).

Group	Name of the drug	Dose ml/kg	Days		
			Background	27	41
1	Control, PBS	–	36.8±0.25	36.8±0.08	37.0±0.12
3	Nano-SOD1	0.13	36.9±0.09	37.0±0.08	37.0±0.10
5	Nano-SOD1	0.53	37.0±0.08	36.9±0.08	37.0±0.12

Table S19. Indicators of rectal temperature in female rats (^{o}C, Mean ± SE).

Group	Name of the drug	Dose ml/kg	Days		
			Background	27	41
2	Control, PBS	–	37.1±0.18	37.1±0.07	37.2±0.08
4	Nano-SOD1	0.13	37.2±0.11	37.2±0.08	37.2±0.09
6	Nano-SOD1	0.53	37.2±0.12	37.2±0.11	37.2±0.11

Behavioral responses in the open field test»

The open field test device is used to assess the behavior of rodents in new (stressful) conditions, as well as symptoms of neurological deficits.

Differences in the behavior of rats treated with solvent and Nano-SOD1 was not detected (Tables S20 and S21).

Table S20. Emotional and motor activity of male rats in the open field test (Mean±SE).

Days	Group 1, Control, PBS	Group 3, Nano-SOD1 0.13 ml/kg	Group 5, Nano-SOD1 0.53 ml/kg
Latency period, s			
background	0.6±0.40	0.2±0.20	0.6±0.40
27	0.4±0.24	0.4±0.24	0.6±0.40
41	0.6±0.40	0.4±0.24	0.8±0.37
Horizontal activity, s			
background	23.2±2.75	23.8±2.99	23.6±3.16
27	22.2±2.52	22.8±2.91	21.4±3.39
41	19.0±2.00	18.2±1.39	20.6±2.09
Vertical activity, s			
background	8.8±0.80	8.6±1.08	9.2±0.86
27	7.6±1.03	9.6±1.50	8.4±0.93
41	7.2±0.86	6.4±0.51	7.2±0.86
Grooming, s			
background	1.2±0.58	1.2±0.37	2.0±0.45
27	1.2±0.37	1.0±0.45	0.4±0.24

Days	Group 1, Control, PBS	Group 3, Nano-SOD1 0.13 ml/kg	Group 5, Nano-SOD1 0.53 ml/kg
41	0.4±0.25	0.6±0.40	10±0.55
Boluses, s			
background	1.6±0.75	1.2±0.37	1.6±0.68
27	1.4±0.75	0.6±0.40	1.2±0.58
41	1.6±0.51	0.8±0.37	1.6±0.51
Urination, s			
background	0.8±0.37	0.4±0.24	0.4±0.25
27	0.6±0.24	0.4±0.24	1.2±0.37
41	0.4±0.24	0.4±0.24	0.4±0.24

Table S21. Emotional and motor activity of female rats in the open field test (Mean±SE).

Days	Group 2, Control, PBS	Group 4, Nano-SOD1 0.13 ml/kg	Group 6, Nano-SOD1 0.53 ml/kg
Latency period, s			
background	0.2±0.20	0.2±0.20	0.6±0.40
27	0.2±0.20	0.2±0.20	0.2±0.20
41	0.4±0.24	0.6±0.40	0.6±0.40
Horizontal activity, s			
background	24.8±3.64	31.2±2.22	27.4±3.52
27	28.4±2.14	22.2±2.48	20.6±1.69
41	24.6±2.58	24.6±3.27	23.0±2.45
Vertical activity, s			
background	11.0±1.22	11.0±1.00	11.0±1.10
27	10.2±1.59	9.0±0.84	9.0±1.30
41	9.2±1.62	8.8±1.24	8.8±1.16
Grooming, s			
background	1.6±0.51	1.4±0.51	1.4±0.51
27	0.4±0.25	1.0±0.32	1.2±0.58
41	1.4±0.51	1.4±0.51	1.0±0.45
Boluses, s			
backg	0.8±0.37	1.4±0.60	1.4±0.75

round			
27	0.6±0.60	0.8±0.49	0.6±0.40
41	0.8±0.49	1.2±0.58	1.2±0.73
Urination, s			
background	0.6±0.40	0.2±0.20	0.8±0.37
27	0.4±0.24	0.6±0.24	0.6±0.24
41	0.4±0.24	0.4±0.24	0.2±0.20

Research of cardiovascular activity

Data on the effect of Nano-SOD1 on heart rate and ECG pattern are presented in Tables S22 and S23.

Table S22. Heart rate and ECG Data of male rats (Mean+SE).

Indicators	Groups		
	Group 1, Control, PBS	Group 3, Nano-SOD1 0.13 ml/kg	Group 5, Nano-SOD1 0.53 ml/kg
Background			
heart rate	501.4±14.27	474.4±38.91	499.8±9.70
R-R, ms	119.8±3.48	130.0±10.17	120.4±2.50
R, ms	11.6±2.56	15.4±2.42	18.4±2.42
P-R (P-Q), ms	28.8±2.40	16.4±2.42*	33.4±1.75*
QRS,	32.6± 4.64	15.4±4.02*	25.2±3.94*
Q-T, ms	84.6±7.16	63.8±4.45*	70.8±5.92*
P, mV	0.05±0.013	0.00±0.000	0.05±0.013
R, mV	0.10±0.024	0.04±0.028	0.11±0.034
T, mV	0.16±0.050	0.07±0.011	0.10±0.008
28 days			
heart rate	512.2±15.24	449.2±38.80	466.6±22.65
R-R, ms	117.6±3.59	137. 8±12.35	129.8±6.69
R, ms	13.0±3.82	16.8±6.73	14.8±1.88
P-R (P-Q), ms	18.6±6.09	18.6±7.59	24.0±5.21
QRS,	19.0±2.76	26.9±2.68	26.0±6.02
Q-T, ms	67.4±5.80	94.4±8.57	80.6±9.37
P, mV	0.01±0.010	0.00±0.000	0.02±0.011
R, mV	0.08±0.051	0.04±0.025	0.03±0.023
T, mV	0.08±0.023	0.06±0.030	0.07±0.042
42 days			
heart rate	488.2± 23.08	461.6±32.09	487.6±16.60
R-R, ms	124.0±6.53	132.6±10.04	124.0±4.59
R, ms	12.2±4.02	12.2±3.99	17.8±4.78
P-R (P-Q), ms	23.0±7.69	20.2±6.47	35.0±1.45
QRS,	16.2±4.96	22.0±4.60	24.6±2.11

Indicators	Groups		
	Group 1, Control, PBS	Group 3, Nano-SOD1 0.13 ml/kg	Group 5, Nano-SOD1 0.53 ml/kg
Q-T, ms	76.5±14.22	84.4± 6.71	71.2±8.29
P, mV	0.02±0.009	0.03±0.021	0.04±0.012
R, mV	0.07±0.045	0.11±0.057	0.14±0.019
T, mV	0.13±0.056	0.10±0.029	0.06±0.024

* - the difference in comparison with the control is significant according to the Student's t criterion (p<0.05)

Table S23. Heart rate and ECG Data of female rats (Mean+SE).

Indicators	Groups		
	Group 2, Control, PBS	Group 4, Nano-SOD1 0.13 ml/kg	Group 6, Nano-SOD1 0.53 ml/kg
Background			
heart rate	432.4±50.64	390.8±51.24	507.2±138.48
R-R, ms	150.2±24.70	170.2±32.49	158.2±41.25
R, ms	18.0±1.97	23.8±4.99	22.2±6.23
P-R (P-Q), ms	31.8±1.69	36.4±2.84	43.6±16.73
QRS,	25.2±3.92	20.8±2.40	34.2±of 9.66
Q-T, ms	77.4±5.52	70.0±5.39	106.0±35.39
P, mV	0.05±0.017	0.07±0.026	0.04±0.020
R, mV	0.06±0.025	0.21±0.063	0.11±0.056
T, mV	0.10±0.028	0.20±0.043	0.11±0.032
28 days			
heart rate	589.4±24.92	520.6±28.39	515.0±39.08
R-R, ms	102.6±4.59	116.6±6.14	119.2±8.68
R, ms	11.0±2.77	10.8±4.51	20.8±4.19
P-R (P-Q), ms	30.4±1.75	17.0±7.80	29.0±6.19
QRS,	22.2±1.66	20.6±4.32	24.4±4.68
Q-T, ms	71.4±5.77	82.2±6.87	71.0±6.75
P, mV	0.05±0.012	0.03±0.018	0.05±0.032
R, mV	0.16±0.039	0.07±0.032	0.09±0.037
T, mV	0.09±0.024	0.11±0.015	0.10±0.039
42 days			
heart rate	499.8±36.64	492.0±10.53	464.4±34.32
R-R, ms	123.2±10.03	122.0±2.85	132.4±11.20
R, ms	18.4±3.93	16.6±1.91	21.4±2.89
P-R (P-Q), ms	32.4±3.44	37.0±1.67	28.6±2.50
QRS,	25.0±2.88	23.8±2.69	22.8±6.04
Q-T, ms	75.6±5.14	73.8±1.83	79.4±5.39
P, mV	0.07±0.023	0.08±0.014	0.05±0.021
R, mV	0.19±0.048	0.20±0.044	0.04±0.022
T, mV	0.17±0.033	0.17±0.029	0.12±0.039

Respiratory system research

Data on the effect of Nano-SOD1 on respiratory rate are presented in Tables S24 and S25. After statistical processing of the data, it was found that the test agent does not affect the frequency of respiratory movements of animals.

Table S24. Data on the respiratory rate of male rats (Mean+SE).

Group	Name of the drug	Dose ml/kg	Day		
			Background	28	42
1	Control, PBS	–	121.8±2.05	123.4±1.73	126.4±1.75
3	Nano-SOD1	0.13	120.3±2.05	124.0±1.58	125.4±1.60
5	Nano-SOD1	0.53	124.9±1.80	127.4±1.72	123.8±1.28

Table S25. Data on the respiratory rate of female rats (Mean+SE).

Group	Name of the drug	Dose ml/kg	Day		
			Background	28	42
2	Control, PBS	–	122.2±1.27	123.2±1.62	126.8±1.20
4	Nano-SOD1	0.13	122.4±1.14	124.1±1.58	124.6±1.72
6	Nano-SOD1	0.53	124.8±0.92	123.8±1.47	127.2±1.02

Clinical blood tests

Tables S26 and S27 show the average group results of a clinical blood test of experimental animals. Male rats slaughtered one day after the last instillation showed a decrease in lymphocytes compared to control rats.

Hemostasis indicators

Repeated use of Nano-SOD1 did not affect the parameters of the hemostatic system (Tables S28 and S29).

Biochemical blood tests

To identify the possible damaging effects of Nano-SOD1 on the liver, heart and bone tissue, the activity of alkaline phosphatase, aspartate aminotransferase, alanine aminotransferase and total bilirubin were determined in the blood serum of animals (Tables S30 and S31). There were no statistically significant changes in the studied indicators in the control and experimental groups. Instillation of Nano-SOD1 for 28 days in an increased dose caused an increase in

glucose content and a decrease in the amount of triglycerides in blood serum in rats (males and females). After 2 weeks of the delayed period, these data did not differ from the control groups of animals (Tables S32 and S33).

The test agent used for 28 days did not have a negative effect on the protein-forming function of the liver and kidney function (Tables S34 and S35).

Table S26. Mean values of clinical blood analysis in male rats (Mean+SE).

Days	Group 1, Control, PBS	Group 3, Nano-SOD1, 0.13 ml/kg	Group 5, Nano-SOD1, 0.53 ml/kg
Red blood cells (RBC), 10^{12}/l			
0	9.0±0.29	9.1±0.16	8.9±0.11
29	9.3±0.12	9.6±0.12	9.5±0.09
43	9.8±0.16	8.9±0.21	9.3±0.37
Platelets (PLT), 10^{9}/l			
0	780.2±31.56	787.2±8.18	782.2±9.35
29	775.6±82.97	790.1±63.5	906.7±58.9
43	731.6±42.34	736.6±17.09	703.4±52.86
Hematocrit (HCT), %			
0	45.8±0.55	45.7±0.91	46.5±0.78
29	43.8±0.62	45.3±0.39	45.1±0.37
43	47.8±1.26	43.1±0.99	43.2±1.40
Hemoglobin (HGB), g/l			
0	167.4±1.40	168.6±1.03	167.0±1.52
29	168.3±2.25	174.6±1.41	173.7±1.02
43	175.0±5.01	164.6±2.73	169.2±4.78
Leukocytes (WBC), 10^{9}/l			
0	12.5±0.43	12.0±0.36	12.7±0.33
29	11.4±0.75	12.1±1.43	13.0±1.02
43	11.7±1.19	12.7±0.70	11.8±1.17
Lymphocytes (LYM), %			
0	68.7±0.80	68.9±0.41	71.4±1.15
29	67.1±0.12	56.7±3.71*	58.0±2.53*
43	58.9±0.76	56.9±1.52	61.0±3.99
Monocytes (MID), %			
0	12.2±0.36	12.0±0.36	12.1±0.22
29	11.0±0.27	12.5±0.87	13.1±1.14
43	12.8±1.75	11.3±0.87	10.9±1.89
Granulocytes (GRAN), %			
0	19.1±1.00	19.1±0.53	16.5±1.28
29	21.0±0.95	29.6±4.10	28.9±2.07
43	28.3±1.06	31.2±1.04	28.0±3.64

* - the difference in comparison with the control is significant according to the Student's t criterion ($p<0.05$)

Table S27. Mean values of clinical blood analysis in female rats (Mean+SE).

Days	Group №2, Control, PBS	Group №4, Nano-SOD1, 0.13 ml/kg	Group № 6, Nano-SOD1, 0.53 ml/kg
Red blood cells (RBC),10^{12}/l			
0	8.6±0.15	8.4±0.09	8.4±0.08
29	8.4±0.09	8.9±0.16	8.3±0.17
43	8.6±0.17	8.9±0.08	8.9±0.18
Platelets (PLT),10^{9}/l			
0	664.2±16.49	657.6±9.80	658.4±8.16
29	628.2±39.67	568.3±38.90	707.1±42.52
43	786.0±66.00	796.0±61.70	794.2±66.38
Hematocrit (HCT), %			
0	44.1±0.89	43.3±1.26	42.7±0.25
29	41.8±0.54	44.3±0.68	40.9±0.78
43	43.1±1.15	43.9±0.47	42.4±0.88
Hemoglobin (HGB), g/l			
0	164.4±2.87	166.0±4.21	160.0±3.22
29	160.6±2.05	169.8±2.12	157.7±2.81
43	163.8±3.61	170.4±1.12	165.4±312
Leukocytes (WBC),10^{9}/l			
0	9.0±0.29	of 9.0±0.22	of 9.1±0.19
29	of 8.7±0.71	of 10.3±0.78	of 10.7±0.71
43	16.8±1.21	16.4±1.90	17.6±1.33
Lymphocytes (LYM), %			
0	57.1±1.20	56.2±1.22	57.6±1.45
29	51.9±3.75	of 60.4±2.18	65.3±3.05
43	61.7±2.83	58.8±1.87	64.2±4.03
Monocytes (MID), %			
0	10.9±0.22	11.0±0.30	10.9±0.20
29	10.0±1.39	11.4±0.56	10.1±0.97
43	12.4±1.38	11.9±0.64	12.7±0.98
Granulocytes (GRAN). %			
0	32.0±1.39	32.8±1.29	31.5±1.32
29	38.1±4.02	28.3±1.78	24.7±2.53
43	26.6±1.56	29.2±2.46	23.1±3.49

Table S28. Mean values of the hemostatic system in male rats (Mean+SE).

Days	Groups		
	Group №1, Control, PBS	Group №3, Nano-SOD1, 0.13 ml/kg	Group № 5, Nano-SOD1, 0.53 ml/kg
Fibrinogen, g/l			
29	2.4±0.12	2.4±0.03	2.3±0.08
43	2.5±0.10	2.5±0.15	2.6±0.18
Prothrombin time, s			
29	20.4±0.76	19.9±1.57	20.9±0.90
43	20.1±0.60	21.2±2.42	21.5±1.42
APTT, s			
29	43.0±2.83	44.5±2.93	47.8±3.26
43	46.9±5.85	44.2±1.97	45.2±3.78

Table S29. Mean values of the female rat hemostatic system (Mean+SE).

Days	Groups		
	Group №2, Control, PBS	Group №4, Nano-SOD1, 0.13 ml/kg	Group № 6, Nano-SOD1, 0.53 ml/kg
Fibrinogen, g/l			
29	2.2±0.04	2.4±0.06	2.1±0.06
43	2.2±0.11	2.3±0.13	2.2±0.02
Prothrombin time,s			
29	20.8±1.31	21.5±0.83	20.5±1.89
43	20.0±0.95	20.1±0.84	17.7±1.37
APTT, s			
29	43.4±3.00	44.4±3.18	45.2±3.09
43	43.6±0.40	45.5±2.63	44.8±2.15

Table S30. Mean values of blood enzyme activity and bilirubin content in male rats (Mean+SE).

Days	Group №1, Control, PBS	Group №3, Nano-SOD1, 0.13 ml/kg	Group № 5, Nano-SOD1 of 0.53 ml/kg
ALT, U/l			
29	45.1±2.04	49.4±2.33	48.2±3.06
43	42.8±5.52	41.0±4.12	47.6±2.80
AST, U/l			
29	275.4±17.20	254.2±22.26	284.4±28.97
43	255.9±19.41	228.5±17.49	258.8±19.10
alkaline phosphatase, U/l			
29	254.8±21.77	282.6±8.64	276.1±14.43
43	250.5±9.43	282.7±of 33.59	324.1±21.85
Total bilirubin, mmol/l			
29	1.3±0.09	1.3±0.09	1.3±0.11
43	1.1±0.35	1.4±0.38	2.1±0.48

Table S31. Mean values of blood enzyme activity and bilirubin content in female rats (Mean+SE).

Days	Group №2, Control. PBS	Group №4, Nano-SOD1, 0.13 ml/kg	Group № 6, Nano-SOD1 0.53 ml/kg
ALT, U/l			
29	47.2±1.78	47.1±1.78	45.4±1.39
43	45.8±5.03	50.6±6.30	43.8±3.30
AST, U/l			
29	254.0±20.38	232.3±5.86	231.3±7.99
43	294.4±26.82	313.4±21.62	213.5±18.19
alkaline phosphatase, U/l			
29	219.1±22.46	222.9±13.83	211.9±11.12
43	254.2± 30.62	271.7±16.59	202.9±912
Total bilirubin, mmol/l			
29	1.2±0.10	1.4±0.10	1.5±0.17
43	1.5±0.25	1.9±0.23	1.8±0.25

Table S32. Mean values of carbohydrate and lipid metabolism in male rats (Mean+SE).

Term. day	Group №1, Control. PBS	Group №3, Nano-SOD1, 0.13 ml/kg	Group № 5, Nano-SOD1, 0.53 ml/kg
Glucose,mmol/l			
29	6.3±0.12	7.7±0.32	8.4±0.31*
43	6.2±0.17	6.5±0.12	6.6±0.09
Cholesterol, mmol/l			
29	1.3±0.11	1.0±0.09	1.3±0.08
43	1.3±0.07	1.4±0.02	1.3±0.05
Triglycerides, mmol/l			
29	0.77±0.047	0.75±0.034	0.60±0.049*
43	0.79±0.0037	0.80±0.028	0.78±0.012

* - the difference in comparison with the control is significant according to the student's t criterion ($p<0.05$)

Table S33. Mean values of carbohydrate and lipid metabolism in female rats (Mean+SE).

Days	Group №2, Control. PBS	Group №4, Nano-SOD1, 0.13 ml/kg	Group № 6, Nano-SOD1, 0.53 ml/kg
Glucose. mmol/l			
29	8.0±0.35	7.9±0.58	9.1±0.15*
43	7.6±0.13	7.7±0.29	7.7±0.12
Cholesterol. mmol/l			
29	1.1±0.08	1.1±0.08	1.1±0.06
43	1.2±0.05	1.3±0.06	1.3±0.04
Triglycerides. mmol/l			
29	0.71±0.041	0.73±0.049	0.56±0.038*
43	0.72±0.007	0.75±0.016	0.73±0.017

* - the difference in comparison with the control is significant according to the student's t criterion ($p<0.05$)

Table S34. Average values of protein metabolism in male rats (Mean+SE).

Term	Group №1, Control. PBS	Group №3, Nano-SOD1, 0.13 ml/kg	Group № 5, Nano-SOD1, 0.53 ml/kg
Total protein,g/l			
29	70.0±1.07	75.5±1.53	77.8±2.15
43	70.0±1.49	70.1±1.70	68.8±1.72
Creatinine,mmol/l			
29	83.7±2.18	76.1±1.40	84.1±2.85
43	73.8±3.92	71.4±4.50	71.7±3.28
Urea, mmol/l			
29	6.5±0.34	6.5±0.32	7.0±0.27
43	8.1±0.76	6.8±0.69	7.0±0.44

Table S35. Average values of protein metabolism in female rats (Mean+SE).

Term	Group №2, Control, PBS	Group №4, Nano-SOD1, 0.13 ml/kg	Group № 6, Nano-SOD1, 0.53 ml/kg
Total protein, g/l			
29	73.0±1.36	72.1±0.91	72.2±3.46
43	69.2±0.79	70.3±0.63	71.4±1.97
Creatinine, mmol/l			
29	72.5±7.25	87.0±1.96	84.6±2.29
43	73.9±2.10	75.6±1.97	72.2±1.55
Urea, mmol/l			
29	7.2±0.43	7.7±0.33	7.2±0.27
43	8.3±0.56	7.9±0.69	8.4±0.78

Biochemical tests of urine

Repeated instillations of Nano-SOD1 into the conjunctival sac of rats did not change the urine parameters (Tables S36, S37). Protein, glucose, bilirubin, urobilinogen, nitrites, ketone bodies, white blood cells, and ascorbic acid were absent in the animals' urine.

Table S36. Results of urine analysis in male rats (Mean+SE).

Days	Groups		
	Group №1, Control. PBS	Group №3, Nano-SOD1, 0.13 ml/kg	Group № 5, Nano-SOD1, 0.53 ml/kg
Specific gravity (SG)			
0	1.017±0.0011	1.016±0.0007	1.015±0.0007
27	1.018±0.0005	1.017±0.0009	1.017±0.0009
41	1.015±0.0011	1.016±0.0014	1.014±0.0008
pH			
0	6.2±0.17	6.3±0.19	6.4±0.27
27	6.1±0.22	6.3±0.21	6.2±0.28
41	6.2±0.30	6.7±0.12	6.1±0.37

Table S37. Results of urine analysis in female rats (Mean+SE).

Days	Groups		
	Group №2, Control, PBS	Group №4, Nano-SOD1, 0.13 ml/kg	Group № 6, Nano-SOD1, 0.53 ml/kg
Specific gravity (SG)			
0	1.015±0.0012	1.016±0.0008	1.016±0.0003
27	1.017±0.0009	1.017±0.0007	1.015±0.0007
41	1.016±0.0017	1.015±0.0010	1.015±0.0004
pH			
0	6.7±0.11	6.2±0.24	6.3±0.29
27	6.3±0.21	6.2±0.30	6.4±0.24
41	6.3±0.30	6.6±0.29	6.4±0.24

Pathomorphological data

Macroscopic examination

Animals were removed from the experiment after 1 day (term 1, day 29) and 14 days (term 2, day 43) after instillations of the test drug into the conjunctival sac of the rat eye.

According to the results of macroscopic examination of organs conducted on days 29 and 43 of the study, no differences were found between the groups, and therefore, the data of autopsy of rats are presented as average for all groups.

At autopsy, the animals had clean skin, the subcutaneous fat layer was moderately developed. The location of the internal organs was correct. No free fluid was found in the pleural and abdominal cavities. The lumen of the trachea and bronchi was free, their mucosa was clean, moist, and shiny.

The intima *of the aorta* was smooth, shiny, whitish in color. The aortic diameter was not changed.

The heart was regular in shape, not enlarged, the myocardium was elastic in consistency, moist, shiny, and the fiber pattern was well defined. Large vessels lying in the pericardial region were well defined. In the cavity of the heart bag there were several drops of clear straw-yellow liquid. The heart valves awere thin, smooth, and shiny.

The larynx was represented by mobile connecting cartilages; the inside was lined with a mucous membrane that formed a series of folds and ventricles.

The lungs The lungs were moderately dormant and had a smooth, soft surface.

The spleen was not enlarged, elongated, the surface was smooth, the consistency was dense, on the incision: the pulp was not scraped off, the follicles and trabeculae had the appearance of gray dots and stripes.

The liver was of regular shape, not enlarged, dense consistency, smooth and shiny. The capsule was thin and transparent.

The kidneys were regular bean-shaped, the fibrous capsule was easily separated. The section clearly showed the border of the cortical and cerebral layers.

The adrenal glands were rounded in shape and of a moderately dense consistency. The medulla was clearly visible on the incision.

The bladder was filled with clear, clear urine. The mucous membrane of the bladder was smooth and shiny.

The esophageal mucosa was shiny, smooth, and pale in color.

Stomach without bloating, normal shape and size. Hyperemia, erosion, and hemorrhage were not observed.

The pancreas was unchanged, lobular.

Intestines without bloating and adhesions. The mucosa was shiny and smooth.

Testicles without special features.

Ovaries were oval, dense, cluster-shaped, pink in color, compact.

The uterus was two-part. The surface was smooth. The color was yellowish-pink. Dense. A cavity with no contents. The horns were thin and long.

The vaginal mucosa was smooth, shiny, collected in distinct longitudinal vaginal folds.

The thymus was triangular in shape, moderately dense in consistency, without hemorrhage.

Submandibular lymph nodes were oval in shape, soft, with a thin capsule.

The thyroid gland was dense with symmetrical lobes, firmly attached to the larynx.

The salivary glands had a round or oval shape, smooth surface.

The membranes of the brain were moderately blood-filled, moist, shiny. The medulla had a symmetrical pattern on the section. Substance of the brain of moderate density. Brain ventricular dilation was not observed.

Morphometric analysis

The relative masses of animal organs are shown in Tables S38-S41. After a course of instillation of the test drug into the conjunctival sac, there was no significant difference in the mass of the internal organs of experimental and control rats.

Table S38. Average group parameters of the relative mass of internal organs of male rats – 1 term. (Mean±SE, %).

Organs	Groups		
	Group № 1, Control, PBS	Group № 3, Nano-SOD1, 0.13 ml/kg	Group № 5, Nano-SOD1, 0.53 ml/kg
Liver	2.49±0.100	2.47±0.058	2.53±0.113
Kidneys	0.63±0.016	0.62±0.016	0.65±0.037
Spleen	0.21±0.009	0.21±0.009	0.20±0.014
Heart	0.31±0.010	0.30±0.011	0.30±0.015
Thymus	0.11±0.005	0.10±0.005	0.10±0.005
Gonads	0.81±0.014	0.87±0.032	0.81±0.036
Lungs	0.49±0.025	0.52±0.039	0.53±0.039

Adrenal glands	0.015±0.0007	0.015±0.0006	0.016±0.0010
Brain	0.45±0.016	0.47±0.016	0.50±0.024

Table S39. Average group parameters of the relative mass of internal organs of female rats -1 term (Mean±SE, %)

Organs	Groups		
	Group № 2, Control, PBS	Group № 4, Nano-SOD1, 0.13 ml/kg	Group № 6, Nano-SOD1, 0.53 ml/kg
Liver	3.09±0.069	2.62±0.044	2.66±0.070
Kidneys	0.59±0.011	0.58±0.019	0.58±0.016
Spleen	0.26±0.011	0.27±0.008	0.27±0.013
Heart	0.30±0.005	0.31±0.009	0.30±0.008
Thymus	0.13±0.006	0.11±0.008	0.11±0.011
Gonads	0.037±0.0025	0.042±0.0023	0.039±0.0038
Lungs	0.58±0.026	0.61±0.062	0.63±0.054
Adrenal glands	0.026±0.0006	0.025±0.0011	0.025±0.0014
Brain	0.61±0.022	0.63±0.015	0.66±0.015

Table S40. Average group parameters of the relative mass of internal organs of male rats - 2 term (Mean±SE, %)

Organs	Groups		
	Group № 1, Control, PBS	Group № 3, Nano-SOD1, 0.13 ml/kg	Group № 5, Nano-SOD1, 0.53 ml/kg
Liver	2.68±0.139	2.15±0.089	2.20±0.07
Kidneys	0.66±0.028	0.61±0.019	0.60±0.019
Spleen	0.22±0.007	0.25±0.015	0.20±0.005

Heart	0.31±0.012	0.30±0.016	0.27±0.007
Thymus	0.10±0.009	0.09±0.012	0.08±0.002
Gonads	0.82±0.025	0.73±0.073	0.79±0.031
Lungs	0.52±0.044	0.59±0.070	0.50±0.042
Adrenal glands	0.016±0.0010	0.016±0.0009	0.015±0.0012
Brain	0.47±0.027	0.49±0.032	0.47±0.024

Table S41. Average group parameters of the relative mass of internal organs of female rats - 2 term (Mean±SE, %)

Organs	Groups		
	Group № 2, Control, PBS	Group № 4, Nano-SOD1, 0.13 ml/kg	Group № 6, Nano-SOD1, 0.53 ml/kg
Liver	2.48±0.087	2.43±0.089	2.45±0.046
Kidneys	0.59±0.022	0.59±0.010	0.55±0.023
Spleen	0.26±0.007	0.25±0.010	0.27±0.015
Heart	0.33±0.010	0.34±0.028	0.32±0.013
Thymus	0.11±0.006	0.13±0.006	0.11±0.006
Gonads	0.041±0.0040	0.040±0.0030	0.037±0.0026
Lungs	0.71±0.041	0.64±0.087	0.76±0.068
Adrenal glands	0.027±0.0025	0.026±0.0015	0.026±0.0017
Brain	0.68±0.025	0.66±0.023	0.65±0.041

Histological examination

As a result of histological studies, it was found that Nano-SOD1 did not cause pathological changes in internal organs, both at a dose of 0.13 mg/kg and at a dose of 0.53 mg/kg. In this regard, the data of microscopic examination are presented in one section.

The brain. Cytoarchitectonics of the cerebral cortex without loss foci. On preparations of the sagittal section of the brain, gray and white matter were differentiated. The soft mater was represented as intertwining collagen and elastic fibers covered with a layer of squamous

epithelium. The vessels of the soft shell in almost all animals were filled with shaped blood elements. The cross-section showed the bodies of large neurons, glial cell nuclei, and capillaries. The nuclei of neurons were light, the nuclear membrane was thin, and the nucleoli were clear. A sufficient amount of Nissl chromatophilic granularity was determined in the cytoplasm: it was pulverized in the cytoplasm of neurons of the 2nd-3rd layers and larger-in the cytoplasm of neurons of the 5th layer of the cerebral cortex. Neurons of different nuclear formations of the middle and medulla oblongata contained large blocks of the tigroid. The nuclei of nerve and glial cells were not changed — the nuclear membrane was thin, the chromatin content was normal, and the nucleoli were clear. In the cerebral cortex, all layers of cells were clearly distinguished. The lumen of small vessels and capillaries was filled with red blood cells. In the vast majority of drugs, the perivascular and pericellular spaces were slightly expanded and optically transparent. In the cerebellar cortex, a molecular layer consisting of a small number of small neurons, pear-shaped Purkinje cells and a granular layer were distinguished. The lumen of small vessels and capillaries was filled with red blood cells.

Heart. The section passed sagittal through the Atria and ventricles, which were filled with shaped blood elements. The epicardium was formed by a layer of single-layer squamous epithelium, and it was preserved in the preparations in all areas. Cardiomyocytes had an elongated shape, light eosinophilic cytoplasm, retain striated striation, contained a sufficient amount of chromatin, and the nuclear envelope was thin. Between cardiomyocytes there was a large number of capillaries, their lumens were filled with red blood cells. There were no foci of violations of tinctorial properties of the cytoplasm. There was no excessive growth of the stroma (cardiofibrosis). Transverse striation of myofibrils in all parts of the heart was distinct.

Endothelial cells of the inner ***aortic membrane*** with distinct nuclei. There awere no destructions of elastic fibers of the middle shell.

Lungs and upper respiratory tract. The epithelium of the larynx, trachea, and large bronchi was not changed, and the nuclei were clear. All animals had a well-defined lobular structure of the lungs. The alveoli of all lobes of the lungs contained air. The nuclei of the alveolar epithelium were clear, the cytoplasm was oxyphilic.

The liver. The lobular structure of the liver was well defined. Hepatocytes with large, well-colored basophilic nuclei, distinct borders, granular cytoplasm, weakly oxyphilic. There were no focal violations of tinctorial properties of the cytoplasm. The cytoplasm of most hepatocytes contained vacuoles of various sizes. The nuclei contained clear nucleoli and a sufficient amount of chromatin. The nuclear membrane was thin. Liver sinusoids were full-blooded. The trabecular structure was preserved. The bile ducts were slit-shaped, lined with a single-layer cubic

epithelium. Sinusoid capillaries were dilated and filled with red blood cells. The lumen of the arteries was mainly filled with red blood cells.

The kidney was surrounded by a well-developed connective tissue capsule. Connective tissue in the kidney parenchyma was poorly developed, located mainly along the course of large vessels. The cortical and brain matter were well differentiated. The renal cortex contained renal corpuscles and convoluted tubules of the nephron. The lumen of the convoluted tubules was free or filled with fine-grained eosinophilic substance. The walls of the Henle loops were formed by a single-layer cubic epithelium, the cytoplasm of which was vacuolated or looks fine-grained, the lumens were filled with a dense eosinophilic substance. Within the medulla, the lumen of the tubules was narrowed, and the nephrocytes look flattened. In the epithelium of the proximal nephrons, the apical surface of the cytoplasm was not visualized. Vessels of the cortical substance were full-blooded.

Vessels of the adrenal cortex and medulla ***of adrenal glands*** were full-blooded. All zones of the adrenal cortex were clearly defined, the cell nuclei contained a sufficient amount of chromatin. The cytoplasm of the cells of the bundle zone was vacuolated due to the content of a large amount of lipids. The cells of the brain substance were large, oval in shape, United in clusters and strands.

The spleen was covered with a dense capsule formed by dense connective tissue covered with mesothelium. In the parenchyma of the spleen, red and white pulp was differentiated. The lymphoid tissue of the spleen, corresponding to the white pulp, was concentrated in rounded follicles, was a cluster of lymphocytes, with an enlightened reactive center. Macrophage-like cells were found on the periphery of the follicles. In the red pulp, the nuclei of reticular stroma cells were distinguishable. The lumen of the vessels was not dilated, they contained shaped blood elements.

Stomach. The mucosa of the fundus and pyloric part of the stomach was examined. The presence of mucosa, submucosal base, muscle and serous membranes was noted. The stomach was lined with a single-layer prismatic epithelium, under which was located its own plate of the mucous membrane with numerous gastric glands. Violations of the integrity of the surface epithelial layer, as well as dystrophic changes and violations of epithelial cell secretion were not detected. The submucosal base was formed by connective tissue, moderately full-blooded, sometimes rare white blood cells were noted in it. The muscular membrane of the stomach was represented by circular and longitudinal layers. The mucous membrane of the iron-free part of the stomach was lined with multilayered squamous epithelium, no cell changes were observed.

There were no defects in the multi-layered flat non-keratinizing epithelium ***of the esophageal mucosa*** .

The wall ***of the small intestine*** consisted of mucosa, submucosa, muscle and serous membranes. The mucous membrane consisted of the epithelium, its own plate and the muscle layer. The inner plate of the small intestinal mucosa was moderately infiltrated by polymorphonuclear cells with a predominance of lymphocytes. The surface of the mucous membrane formed villi, folds and crypts. The villi were covered with a single-layered cylindrical epithelium, in which edged and goblet-shaped cells differentiate. The submucosa of the small intestine was formed by loose fibrous connective tissue, slightly infiltrated by polymorphonuclear cells, and was full-blooded. The muscular membrane of the small intestine was represented by circular and longitudinal layers.

The epithelium ***of the large intestine*** was single-row cylindrical. The apical surface of the crypts was lined with edged epithelial cells. The lateral surface of the crypts contained actively secreting, goblet-shaped cells. Lymphoid follicles were located in the submucosal base of the colon. Accumulations of lymphoid tissue had a rounded or oval shape, spread up to the epithelial layer, and consisted mainly of evenly distributed lymphocytes. The blood vessels of the large intestine had wide lumens, contained shaped blood elements. The muscle layers (circular and longitudinal) were thin, and the vegetative ganglia were evenly distributed between them.

The lobular structure ***of the pancreas*** was preserved. The gland was covered with a thin layer of connective tissue. Most of the parenchyma was represented by acinuses formed by a single layer of glandulocytes lying on the basement membrane. Broad basal sections of glandulocytes were intensively stained with hematoxylin and contained rounded nuclei. In the apical part, the cytoplasm was more acidophilic, and granules of secretions were often seen. In part of the glandulocytes, the apical part of the cells contained optically transparent vacuoles. Acinus lumen was small, often almost not visualized. The mucosa of large ducts was formed by a single-layer prismatic epithelium. In the thickness of the gland, Langerhans islets were located in the form of compact groups of light polygonal cells, the size of the islets varied.

Testes. From the surface, the testes were limited by a dense connective tissue membrane, from which thin partitions extended. The connective tissue septa contained clusters of large Leydig cells. The spermatic tubule lumen was unevenly dilated. On the basement membrane there was a layer of spermatogonia alternating with Sertoli support cells. In the direction of the lumen of the tubules, successive stages of maturation of spermatocytes were differentiated, ending with the appearance of spermatozoa.

The ovaries. The organ was topographically divided into cortical and medullary matter. In the root section of the control animals, numerous follicles could be traced, which were at different stages of maturation.

The thymus retained a pronounced lobular structure. The lobes of the thymus were covered with a connective tissue capsule, which had processes towards the parenchyma, which form septa. In the thymus, the cortical and brain matter differentiated, and the boundary between them was indistinct. The cortical substance was represented by evenly distributed lymphocytes, lymphoblasts and dendritic epithelial cells. The epithelial cells were large and pale with distinct basophilic nuclei. The capillaries of the cortical substance were filled with red blood cells. Individual gassal corpuscles of immature type were visible in the medulla.

Conclusion on the study of chronic toxicity in rats

No animal deaths or intoxication symptoms were reported during the chronic toxicity of Nano-SOD1.

After applying the test agent, the physiological parameters of the animals did not change.

Repeated use of Nano-SOD1 did not lead to changes in the mass and structure of the internal organs of rats.

Histological evaluation did not reveal a pathological effect of the studied drug on the internal organs of animals.

Results of a chronic toxicity study in rabbits

Clinical observations

Survival rate. During the study, all the animals remained alive.

Appearance and behavior. In all groups, rabbits actively ate food, reacted to external stimuli, and showed interest in people. Muscle tone was not characterized by increased excitability. The rabbits were of average fatness and did not suffer from exhaustion. The coat was smooth and shiny; no hair loss or breakage has been detected. No corneal opacities, lacrimation, or any eye abnormalities were observed. The auricles were pink without crusts, not inflamed, twitching was not noticed. Teeth of normal color, no breakdowns were observed. Respiration in animals was normal, uncomplicated; salivation without pathology; frequency, quantity and consistency of fecal masses within the physiological norm.

During instillation, the animals did not show any anxiety, vocalization was absent. Examination of the eyes revealed no hyperemia, edema, or discharge.

Dynamics of body weight

The results of weighing rabbits are shown in Tables S42 and S43. The body weight of rabbits during the experiment had a positive trend and did not significantly differ between the groups.

Table S42. Dynamics of body weight in male rabbits (Mean±SE, g)

Days	Groups		
	Group № 1, Control, PBS	Nano-SOD1	
		Group № 3, 0.04 ml/kg	Group № 5, 0.15 ml/kg
0	2026.8±23.16	2090.3±29.18	2063.0± 19.27
7	2214.0±6.35	2253.5±19.47	2232.3±7.66
14	2318.3± 10.68	2347.0±15.87	2338.8±12.96
21	2508.8±t 40.23	2510.3±49.31	2498.3±41.34
29	2640.5±39.91	2666.0±55.06	2667.0± 37.16

Table S43. Dynamics of body weight of female rabbits (Mean±SE, g).

Days	Groups		
	Group № 2, Control, PBS	Nano-SOD1	
		Group № 4, 0.04 ml/kg	Group № 6, 0.15 ml/kg
0	2529.2±21.83	2457.3±43.09	2424.5±138.55
7	2757.0±31.20	2641.5±76.25	2557.8±148.11
14	2918.3±57.96	2735.3±68.93	2766.5±166.26
21	3230.0±61.64	2849.3±88.56	2979.5±202.50
29	3307.3±76.72	2930.5±116.08	3049.5±227.41

Rectal temperature measurements

Data for measuring the rectal temperature of rabbits are presented in Tables S44 and S45. Temperature readings of the animals during the study were within the physiological norm.

Table S44. Indicators of rectal temperature of male rabbits (°C, Mean±SE).

Groups	Dose, ml/kg	The observation time, day	
		0	27
№ 1 – control, PBS	---	39.2±0.04	39.2±0.03
№ 3 – Nano-SOD1	0.04	39.1±0.07	39.2±0.09
№ 5 – Nano-SOD1	0.15	39.2±0.09	39.2±0.04

Table S45. Indicators of rectal temperature of female rabbits (°C, Mean±SE).

Groups	Dose, ml/kg	The observation time, day	
		0	27
№ 2 – control, PBS	---	39.2±0.07	39.2±0.09
№ 4 – Nano-SOD1	0.04	39.3±0.06	39.2±0.07
№ 6 – Nano-SOD1	0.15	39.2±0.06	39.2±0.08

Feed and water consumption

Feed and water consumption were taken into account once a week. The data are shown in Tables S46-S49. As can be seen from the presented results, the amount of food eaten and water drunk by animals did not differ between the groups.

Table S46. Feed consumption by male rabbits (Mean±SE, g).

Groups	Dose, ml/kg	The observation time, day				
		0	8	15	22	27
№ 1 – control, PBS	---	188.8±6.05	200.5± 5.69	200.5±3.97	203.5±2.50	204.5±3.38
№ 3 – Nano-SOD1	0,04	190.8±5.02	197.3±5.28	205.8±4.37	206.8±3.64	209.8±5.27
№ 5 – Nano-SOD1	0,15	186.5±5.04	201.5±5.45	203.8±4.61	209.5±5.45	207.5±4.80

Table S47. Feed consumption by female rabbits (Mean±SE, g).

Groups	Dose, ml/kg	The observation time, day				
		0	8	15	22	27
№ 2 – control, PBS	---	173.0± 7.94	188.5±7.42	189.0±9.35	186.5±6.40	196.0±4.60
№ 4 – Nano-SOD1	0.04	169.0±6.07	176.8±5.17	176.8±6.05	183.3±4.39	185.3±3.59
№ 6 – Nano-SOD1	0.15	173.8±9.98	188.0±7.97	182.8±7.30	193.0±7.08	198.3± 4.66

Table S48. Water consumption by male rabbits (Mean±SE, ml).

Groups	Dose, ml/kg	The observation time, day				
		0	8	15	22	27
№ 1 – control, PBS	---	390.0±9.66	376.3±3.57	382.5±6.55	395.0±3.81	398.5±5.04
№ 3 – Nano-	0.04	395.5±10.6	373.0±15.17	394.3±3.17	406.3±5.27	401.5±6.09

SOD1		5				
№ 5 – Nano-SOD1	0.15	391.0±5.58	373.0±17.09	394.3±8.40	406.0±7.15	406.5±4.44

Table S49. Water consumption by female rabbits (Mean±SE, ml).

Groups	Dose, ml/kg	The observation time, day				
		0	8	15	22	27
№ 2 – control, PBS	---	350.8±9.59	362.3±8.63	363.8±6.98	369.0±3.58	374.5±3.50
№ 4 – Nano-SOD1	0,04	343.0±10.90	357.8±8.72	363.3±7.02	372.8±6.29	374.3±2.90
№ 6 – Nano-SOD1	0,15	363.3±4.57	364.3±10.04	364.3±4.13	379.8±6.25	285.3±7.35

Clinical blood tests

Tables S50 and S51 show the average group results of a clinical rabbit blood test. Multiple instillations of Nano-SOD1 did not affect hematopoiesis in animals.

Table S50. Average values of clinical blood analysis in male rabbits (Mean±SE).

Days	Group		
	Group № 1, Control, PBS	Nano-SOD1	
		Group № 3, 0.04 ml/kg	Group № 5, 0.15 ml/kg
Red blood cells (RBC),10^{12}/l			
Background	6.5±0.16	6.5±0.06	6.6±0.10
29	6.6±0.05	6.5±0.41	6.7±0.43
Platelets (PLT),10^{9}/l			
Background	287.0±23.59	275.3±8.96	276.8±11.76
29	288.3±25.73	247.5±18.46	269.8±9.76
Hematocrit (HCT), %			
Background	42.1±1.22	44.1±0.66	44.1±0.67
29	40.5±0.42	39.8±2.03	40.2±0.60
Hemoglobin (HGB), g/l			

Background	147.0±1.29	148.8±1.11	146.0±0.71
29	148.8±1.65	150.5±5.20	146.8±3.66
Leukocytes (WBC), 10^9/l			
Von	4.9±0.18	5.1±0.23	4.7±0.11
29	5.0±0.14	Of 5.2±0.18	6.7±0.39
Lymphocytes (LYM), %			
Background	63.2±0.30	62.4±0.55	62.7±0.70
29	of 62.6±1.94	54.2±of 6.61	58.3±4.31
Monocytes (MID), %			
Background	11.9±0.12	11.5±0.50	11.5±0.25
29	11.5±0.25	11.4±0.13	12.4±0.58
Granulocytes (GRAN), %			
Background	23.9±1.03	22.8±1.11	24.6±0.90
29	23.7±1.97	22.9±1.47	24.8±1.64

Table S51. Average values of clinical blood analysis in female rabbits (Mean±SE).

Days	Group		
	Group № 2, Control, PBS	Nano-SOD1	
		Group № 4, 0.04 ml/kg	Group № 6, 0.15 ml/kg
Red blood cells (RBC),10^{12}/l			
Background	6.5±0.16	6.5±0.06	6.6±0.10
29	6.6±0.05	6.5±0.41	6.7±0.43
Platelets (PLT),10^9/l			
Background	287.0±23.59	275.3±8.96	276.8±11.76
29	288.3±25.73	247.5±18.46	269.8±9.76
Hematocrit (HCT), %			
Background	42.1±1.22	44.1±0.66	44.1±0.67
29	40.5±0.42	39.8±2.03	40.2±0.60
Hemoglobin (HGB), g/l			
Background	147.0±1.29	148.8±1.11	146.0±0.71
29	148.8±1.65	150.5±5.20	146.8±3.66
Leukocytes (WBC), 10^9/l			
Background	4.9±0.18	5.1±0.23	4.7±0.11

29	5.0±0.14	Of 5.2±0.18	6.7±0.39
Lymphocytes (LYM), %			
Background	63.2±0.30	62.4±0.55	62.7±0.70
29	62.6±1.94	54.2± 6.61	58.3±4.31
Monocytes (MID), %			
Background	11.2±0.50	11.6±0.51	11.1±0.25
29	11.0±0.53	12.3±0.85	11.9±0.99
Granulocytes (GRAN), %			
Background	25.6±0.63	26.0±0.58	26.2±0.62
29	26.4±2.14	32.8±5.96	29.9±3.34

Hemostasis indicators

Repeated use of Nano-SOD1 did not affect the parameters of the hemostatic system (Tables S52 and S53).

Table S52. Average parameters of the hemostatic system in male rabbits (Mean±SE).

Groups	Dose, ml/kg	Groups		
		Fibrinogen, g/l	PT, s	APTT, s
№ 1 – control, PBS	---	2.2±0.04	9.8±0.09	42.3±0.59
№ 3 – Nano-SOD1	0.04	2.3±0.07	9.9±0.11	41.6±1.04
№ 5 – Nano-SOD1	0.15	2.2±0.09	9.8±0.03	41.7±1.12

Table S53. Average parameters of the hemostatic system in female rabbits (Mean±SE).

Groups	Dose, ml/kg	Groups		
		Fibrinogen, g/l	PT, s	APTT, s
№ 2 – control, PBS	---	2.2±0.09	9.9±0.09	41.5±0.56
№ 4 – Nano-SOD1	0.04	2.1±0.11	9.7±0.44	41.7±1.03
№ 6 – Nano-SOD1	0.15	2.2±0.09	10.1±0.31	41.9±0.58

Biochemical blood tests

At the end of instillations, the activity of enzymes, indicators of carbohydrate, lipid and protein metabolism were determined in the blood serum of animals (Tables S54 and S55). There was no statistically significant difference between the control and experimental groups in the studied indicators.

Table S54. Average values of biochemical blood tests of male rabbits (M±m).

Parametrs	Group		
	Group № 1, Control, PBS	Nano-SOD1	
		Group № 3, 0.04 ml/kg	Group № 5, 0.15 ml/kg
ALT, U/l	37.5±2.24	37.8±3.20	39.2±3.28
AST U/l	38.4±2.81	40.3±1.53	41.0±1.02
alkaline phosphatase, U/l	540.5±9.29	523.5±19.52	556.5± 8.42
Total bilirubin, mmol/l	1.3±0.07	1.2±0.03	1.2±0.03
Glucose, mmol/l	5.7±0.19	5.8±0.14	5.7±0.14
Cholesterol, mmol/l	1.2±0.02	1.1±0.03	1.2±0.02
Triglycerides, mmol/l	0.80±0.034	0.76±0.028	0.78±0.027
Total protein, g/l	62.9±0.68	63.9±1.57	63.8±1.08
Creatinine, mmol/l	104.4±4.43	100.4±1.41	101.9±1.31
Urea, mmol/l	8.2±0.62	8.4±0.43	8.2±0.33

Table S55. Average values of biochemical blood tests of female rabbits (M±m).

Term, day	Group		
	Group № 2, Control, PBS	Nano-SOD1	
		Group № 4, 0.04 ml/kg	Group № 6, 0.15 ml/kg
ALT, U/l	31.9±9.16	44.9±5.76	42.2±2.58
AST U/l	39.0±6.37	40.5±6.59	34.5±2.27
alkaline phosphatase, U/l	556.5±51.50	477.9±26.9	480.0±55.84
Total bilirubin, mmol/l	1.3±0.09	1.4±0.16	1.7±0.26
Glucose, mmol/l	5.9±0.13	5.8±0.21	5.8±0.22
Cholesterol, mmol/l	1.2±0.04	1.1±0.04	1.1±0.04
Triglycerides, mmol/l	0.78±0.028	0.77±0.026	0.80±0.010

Total protein, g/l	62.9±0.98	62.6±0.71	62.7±0.69
Creatinine, mmol/l	104.4±4.69	102.7±2.79	103.0±2.83
Urea, mmol/l	7.6±0.23	8.0±0.11	7.9±0.16

Biochemical tests of urine

Instillation of Nano-SOD1 into the conjunctival sac of rabbits ' eyes did not affect urine parameters (Tables S46 and S47). Protein, glucose, bilirubin, urobilinogen, nitrites, ketone bodies, white blood cells, and ascorbic acid were absent in the animals ' urine.

Table S56. Results of the urine analysis of male rabbits (M±m).

Days	Group		
	Group № 1, Control, PBS	Nano-SOD1	
		Group № 3, 0.04 ml/kg	Group № 5, 0.15 ml/kg
Specific gravity (SG)			
0	1.015±0.0013	1.016±0.0010	1.017±0.0005
14	1.016±0.0007	1.016±0.0010	1.017±0.0011
pH			
0	8.3±0.14	8.1±0.13	8.4±0.13
14	8.0±0.00	8.3±0.14	of 8.3±0.14

Table S57. Results of urine analysis of female rabbits (M±m).

Days	Group		
	Group № 2, Control, PBS	Nano-SOD1	
		Group № 4, 0.04 ml/kg	Group № 6, 0.15 ml/kg
Specific gravity (SG)			
0	1.017±0.0003	1.016±0.0012	1.018±0.0003
14	1.018±0.0003	1.018±0	1.018±0.0003
pH			
0	8.3±0.14	8.4±0.13	8.1±0.13
14	8.4±0.13	8.3±0.14	8.4±0.13

Research of cardiovascular and respiratory activity

The heart rate and ECG data of rabbits after applications of the Test drug are presented in Tables S58 and S59. After using the test drug, the animals ' heart rate and ECG pattern did not significantly change.

Table S58. Heart rate and ECG Data of male rabbits (M±m).

Days	Group		
	Group № 1, Control, PBS	Nano-SOD1	
		Group № 3, 0.04 ml/kg	Group № 5, 0.15 ml/kg
Background			
heart rate	138.0±31.15	202.5±30.00	185.5±17.44
BH	73.8±6.58	75.0±10.21	72.5± 8.78
P, ms	51.8±4.87	44.5± 8.39	41.3± 4.59
P-R (P-Q), ms	96.8±16.11	82.5± 7.88	80.8±9.28
QRS, ms	48.5±8.19	43.5±10.37	45.0±11.74
Q-T, ms	171.0±15.92	187.0±16.75	175.0±35.26
P, mV	0.19±0.031	0.15±0.044	0.16±0.020
R, mV	0.12±0.032	0.13±0.013	0.15±0.031
T mV	0.37±0.202	0.33±0.183	0.36±0.185
28 days			
heart rate	408.3± 90.39	375.3±50.64	353.8±65.92
BH	92.5±5.95	92.5±3.23	87.5± 6.61
P, ms	25.5±5.39	21.0±9.44	25.0± 9.71
P-R (P-Q), ms	29.3± 8.37	28.8±9.93	39.5±9.10
QRS, ms	24.8± 11.09	25.8±2.69	28.5±6.98
Q-T, ms	88.8±25.89	83.0±15.14	98.0±20.90
P, mV	0.10±0.009	0.11±0.042	0.11±0.021
R, mV	0.07±0.076	0.11±0.060	0.10±0.043
T mV	0.09±0.080	0.08± 0.069	0.08±0.064

Table S59. Heart rate and ECG Data of female rabbits (M±m).

Days	Group		
	Group № 2, Control, PBS	Nano-SOD1	
		Group № 4, 0.04 ml/kg	Group № 6, 0.15 ml/kg
Background			
heart rate	236.0±12. 21	223.8± 4.09	248.3± 10.68
BH	85.0±10.99	78.8±5.54	82.5± 6.61
P, ms	46.5± 4.99	46.3± 4.09	45.3± 5.74
P-R (P-Q), ms	93.8±13.79	89.0±15.40	92.0± 25.01
QRS, ms	46.0±8.01	49.3±17.82	47.3±11.99
Q-T, ms	167.8±15.82	187.0±16.75	175.0±35.26
P, mV	0.13±0.047	0.13±0.044	0.14±0.021
R, mV	0.13±0.022	0.14±0.017	0.14±0.032
T mV	0.33±0.116	0.36±0.190	0.34±0.186
28 days			
heart rate	331.8±67.28	375.3±50.63	353.8±65.92
BH	80.0± 7.36	77.5±10.51	77.5± 8.78
P, ms	33.5±6.12	36.3±6.25	37.0±5.40
P-R (P-Q), ms	30.5±9.47	30.0±8.09	33.0±13.61
QRS, ms	24.8±8.53	22.8±3.15	27.3±7.60
Q-T, ms	83.5± 22.03	85.3±14.89	92.5±22.25
P, mV	0.14±0.032	0.14±0.020	0.15±0.025
R, mV	0.08±0.081	0.09±0.054	0.09±0.040
T mV	0.14±0.093	0.15±0.093	0.16±0.082

Ophthalmic indicators

External eye examination revealed no lacrimation, hyperemia, edema, or discharge.

Biomicroscopy

The condition of the anterior ocular structures in all animals, both before the study and after 28-day instillations into the conjunctival sac of the eye with Nano-SOD1, corresponded to the physiological norm:

- cornea – smooth, shiny, mirror-like, without opacities;
- the iris was evenly pigmented, the pupil was round, and reacted to light (the pupil narrows when the illumination increases, and expands when it decreases);
- conjunctiva – single vessels were visible, there was no hyperemia; the conjunctiva of the eyelids was pale pink, transparent, non-edematous; there was no discharge;
- fluorescein test – no damage to the integrity of the corneal epithelium was detected: after instillation of a 0.5% solution of fluorescein, a uniform greenish glow of the entire corneal surface was determined (the fluorescein test is negative).

Ophthalmoscopy

Ophthalmoscopy performed both before instillation of Nano-SOD1, and after their use for 28 days, did not reveal any pathological changes in the fundus. The disc of the optic nerve was pale pink with clear borders, the vessels of the fundus were moderately blood-filled, without nodes, the course of the vessels was not changed, foci and hemorrhages were not detected. The retina lied flat on the entire surface.

Pupillometry

Tables S60-S61 show the results of measuring the pupil size of rabbits. As can be seen from the Tables, these parameters in the animals of the experimental groups are identical to the parameters in the animals of the control groups.

Intraocular pressure

Data on IOP measured before the start of the experiment and after the end of using Nano-SOD1 are shown in Tables S62 and S63. Instillation of the tested product did not lead to a change in IOP.

Table S60. Pupilometry Data of male rabbits after instillation of Nano-SOD1 (M±m).

Days	Group					
	Group № 1, Control, PBS		Group № 3, Nano-SOD1 0.04 ml/kg		Group № 5, Nano-SOD1 0.15 ml/kg	
	OS	OD	OS	OD	OS	OD
Horizontal Meridian, cm						
0	0.70±0.040	0.67±0.025	0.75±0.029	0.75±0.029	0.70±0.040	0.73±0.025
28	0.70±0.041	0.70±0.041	0.73±0.025	0.75±0.025	0.73±0.025	0.75±0.025
Vertical Meridian, cm						
0	0.80±0.040	0.75±0.029	0.85±0.029	0.85±0.029	0.80±0.041	0.83±0.025
28	0.80±0.041	0.80±0.041	0.83±0.025	0.83±0.025	0.83±0.025	0.83±0.025

Table S61. Pupilometry Data of female rabbits after instillation of Nano-SOD1 (M±m).

Days	Group					
	Group № 2, Control, PBS		Group № 4, Nano-SOD1 0.04 ml/kg		Group № 6, Nano-SOD1 0.15 ml/kg	
	OS	OD	OS	OD	OS	OD
Horizontal Meridian, cm						
0	0.65±0.029	0.70±0.041	0.67±0.025	0.70±0.041	0.70±0.041	0.68±0.025
28	0.70±0.041	0.73±0.025	0.75±0.029	0.75±0.029	0.75±0.025	0.75±0.029
Vertical Meridian, cm						
0	0.78±0.025	0.80±0.083	0.80±0.041	0.78±0.063	0.80±0.041	0.78±0.025
28	0.80±0.041	0.83±0.025	0.85±0.029	0.85±0.029	0.85±0.029	0.85±0.029

Table S62. Intraocular pressure of male rabbits, mmHg (M±m).

Eye	Group		
	Group № 1, Control, PBS	Nano-SOD1	
		Group № 3, 0.04 ml/kg	Group № 5, 0.15 ml/kg
0			
OS	17.3±0.25	17.3±0.25	17.3±0.25
OD	17.3±0.25	17.3±0.25	17.3±0.25
28 days			
OS	17.3±0.75	17.3±0.25	17.5±0.29
OD	17.3±0.75	17.3±0.25	17.5±0.29

Table S63. IOP Data for female rabbits, mmHg (M±m).

Eye	Group		
	Group № 2, Control, PBS	Nano-SOD1	
		Group № 4, 0.04 ml/kg	Group № 6, 0.15 ml/kg
0			
OS	16.8±0.25	17.5±0.29	17.0±0.00
OD	16.8±0.25	17.5±0.29	17.0±0.00
28 days			
OS	17.3±0.75	17.5±0.29	17.3±0.25
OD	17.3±0.75	17.5±0.29	17.3±0.25

Pathomorphological data

Macroscopic examination

Animals were removed from the experiment one day after the last instillation of Nano-SOD1 into the conjunctival sac of the eye.

According to the results of macroscopic examination of organs, no differences between the groups were established, and therefore, the data of rabbit autopsies are presented as average for all groups.

At autopsy, the animals had clean skin, the subcutaneous fat layer was moderately developed. The location of the internal organs was correct. No free fluid was found in the pleural

and abdominal cavities. The lumen of the trachea and bronchi was free, their mucosa was clean, moist, and shiny.

The intima ***of the aorta*** was smooth, shiny, whitish in color. The aortic diameter was not changed.

The heart was regular in shape, not enlarged, the myocardium was elastic in consistency, moist, shiny, and the fiber pattern was well defined. Large vessels lying in the pericardial region were well defined. In the cavity of the heart bag there were several drops of clear straw-yellow liquid. The heart valves were thin, smooth, and shiny.

The larynx was represented by mobile connecting cartilages; the inside was lined with a mucous membrane that formed a series of folds and ventricles.

The lungs were moderately dormant and have a smooth, soft surface.

The spleen was not enlarged, elongated, the surface was smooth, the consistency was dense, on the incision: the pulp was not scraped off, the follicles and trabeculae had the appearance of gray dots and stripes.

The liver was of regular shape, not enlarged, dense consistency, smooth and shiny. The capsule was thin and transparent.

The kidneys were regular bean-shaped, the fibrous capsule was easily separated. The section clearly shows the border of the cortical and cerebral layers.

The adrenal glands are rounded in shape and of a moderately dense consistency. The medulla was clearly visible on the incision.

The bladder was filled with clear, clear urine. The mucous membrane of the bladder was smooth and shiny.

The esophageal mucosa was shiny, smooth, and pale in color.

Stomach without bloating, normal shape and size. Hyperemia, erosion, and hemorrhage were not observed.

The pancreas was unchanged, lobular.

Intestines without bloating and adhesions. The mucosa was shiny and smooth.

Testicles without special features.

Ovaries were oval, dense, pink in color, compact.

The uterus was two-part. The surface was smooth. The color was yellowish-pink. Dense.

A cavity with no contents. The horns were thin and long.

The vaginal mucosa was smooth, shiny, collected in distinct longitudinal vaginal folds.

The thymus was triangular in shape, moderately dense in consistency, without hemorrhage.

Submandibular lymph nodes were oval in shape, soft, with a thin capsule.

The thyroid gland was dense with symmetrical lobes, firmly attached to the larynx.

The salivary glands had a round or oval shape, smooth surface.

The membranes of the brain were moderately blood-filled, moist, shiny. The medulla had a symmetrical pattern on the section. Substance of the brain of moderate density. Brain ventricular dilation was not observed.

Morphometric analysis

The relative masses of animal organs are shown in Tables S64 and S65. The use of Nano-SOD1 for 28 days did not cause significant changes in the mass of internal organs.

Table S64. Average group parameters of the relative mass of internal organs of male rabbits (% , M±m).

Organs	Group		
	Group № 1. Control. PBS	Nano-SOD1	
		Group № 3. 0.04 ml/kg	Group № 5. 0.15 ml/kg
The liver	2.58±0.161	2.35±0.198	2.53±0.181
Kidneys	0.55±0.047	0.56±0.022	0.54±0.026
The spleen	0.07±0.006	0.06±0.004	0.06±0.001
The heart	0.25±0.006	0.24±0.004	0.24±0.005
Thymus	0.20±0.017	0.19±0.007	0.19±0.005
Gonads	0.102±0.0045	0.102±0.0036	0.099±0.0019
Lungs	0.33±0.026	0.33±0.022	0.35±0.004
Adrenal-glands	0.009±0.0004	0.009±0.0003	0.009±0.0003
The brain	0.36±0.012	0.37±0.007	0.37±0.006

Table S 65. Average group parameters of the relative mass of internal organs of female rabbits (% , M±m).

Organs	Group		
	Group № 2, Control. PBS	Nano-SOD1	
		Group № 4, 0.04 ml/kg	Group № 6, 0.15 ml/kg
The liver	2.83±0.237	2.33±0.071	2.14±0.241
Kidneys	0.52±0.053	0.54±0.020	0.53±0.015
The spleen	0.05±0.010	0.06±0.005	0.05±0.005
The heart	0.23±0.002	0.22±0.007	0.21±0.012
Thymus	0.19±0.014	0.10±0.019	0.13±0.027
Gonads	0.008±0.0004	0.009±0.0015	0.006±0.0009
Lungs	0.25±0.036	0.32±0.014	0.32±0.014
Adrenal-glands	0.006±0.0006	0.007±0.0006	0.008±0.0007
The brain	0.28±0.008	0.31±0.017	0.31±0.020

Histological examination

As a result of histological studies, it was found that Nano-SOD1 did not cause pathological changes in internal organs. In this regard, the data of microscopic examination are presented in one section.

The brain. Cytoarchitectonics of the cerebral cortex without loss foci. On preparations of the sagittal section of the brain, gray and white matter were differentiated. The soft mater was represented as intertwining collagen and elastic fibers covered with a layer of squamous epithelium. The vessels of the soft shell in almost all animals were filled with shaped blood elements. The cross-section showed the bodies of large neurons, glial cell nuclei, and capillaries. The nuclei of neurons were light, the nuclear membrane was thin, and the nucleoli were clear. A sufficient amount of Nissl chromatophilic granularity was determined in the cytoplasm: it was pulverized in the cytoplasm of neurons of the 2nd-3rd layers and larger-in the cytoplasm of

neurons of the 5th layer of the cerebral cortex. Neurons of different nuclear formations of the middle and medulla oblongata contained large blocks of the tigroid. The nuclei of nerve and glial cells were not changed — the nuclear membrane was thin, the chromatin content was normal, and the nucleoli were clear. In the cerebral cortex, all layers of cells were clearly distinguished. The lumen of small vessels and capillaries was filled with red blood cells. In the vast majority of drugs, the perivascular and pericellular spaces were slightly expanded and optically transparent. In the cerebellar cortex, a molecular layer consisting of a small number of small neurons, pear-shaped Purkinje cells and a granular layer were distinguished. The lumen of small vessels and capillaries was filled with red blood cells.

Heart. The section passed sagittal through the Atria and ventricles, which were filled with shaped blood elements. The epicardium was formed by a layer of single-layer squamous epithelium, and it was preserved in the preparations in all areas. Cardiomyocytes had an elongated shape, light eosinophilic cytoplasm, retained striated striation, contained a sufficient amount of chromatin, and the nuclear envelope was thin. Between cardiomyocytes there was a large number of capillaries, their lumens were filled with red blood cells. There were no foci of violations of tinctorial properties of the cytoplasm. There was no excessive growth of the stroma (cardiofibrosis). Transverse striation of myofibrils in all parts of the heart was distinct.

Endothelial cells of the inner ***aortic membrane*** with distinct nuclei. There were no destructions of elastic fibers of the middle shell.

Lungs and upper respiratory tract. The epithelium of the larynx, trachea, and large bronchi was not changed, and the nuclei were clear. All animals had a well-defined lobular structure of the lungs. The alveoli of all lobes of the lungs contained air. The nuclei of the alveolar epithelium were clear, the cytoplasm was oxyphilic.

The liver. The lobular structure of the liver was well defined. Hepatocytes with large, well-colored basophilic nuclei, distinct borders, granular cytoplasm, weakly oxyphilic. There were no focal violations of tinctorial properties of the cytoplasm. The cytoplasm of most hepatocytes contained vacuoles of various sizes. The nuclei contained clear nucleoli and a sufficient amount of chromatin. The nuclear membrane was thin. Liver sinusoids were full-blooded. The trabecular structure was preserved. The bile ducts were slit-shaped, lined with a single-layer cubic epithelium. Sinusoid capillaries were dilated and filled with red blood cells. The lumen of the arteries was mainly filled with red blood cells.

The kidney was surrounded by a well-developed connective tissue capsule. Connective tissue in the kidney parenchyma was poorly developed, located mainly along the course of large vessels. The cortical and brain matter were well differentiated. The renal cortex contained renal corpuscles and convoluted tubules of the nephron. The lumen of the convoluted tubules was free

or filled with fine-grained eosinophilic substance. The walls of the Henle loops were formed by a single-layer cubic epithelium, the cytoplasm of which was vacuolated or looks fine-grained, the lumens were filled with a dense eosinophilic substance. Within the medulla, the lumen of the tubules was narrowed, and the nephrocytes looked flattened. In the epithelium of the proximal nephrons, the apical surface of the cytoplasm was not visualized. Vessels of the cortical substance were full-blooded.

Vessels of the adrenal cortex and medulla ***adrenal glands*** were full-blooded. All zones of the adrenal cortex were clearly defined, the cell nuclei contained a sufficient amount of chromatin. The cytoplasm of the cells of the bundle zone was vacuolated due to the content of a large amount of lipids. The cells of the brain substance were large, oval in shape, united in clusters and strands.

The spleen was covered with a dense capsule formed by dense connective tissue covered with mesothelium. In the parenchyma of the spleen, red and white pulp was differentiated. The lymphoid tissue of the spleen, corresponding to the white pulp, was concentrated in rounded follicles, was a cluster of lymphocytes, with an enlightened reactive center. Macrophage-like cells were found on the periphery of the follicles. In the red pulp, the nuclei of reticular stroma cells were distinguishable. The lumen of the vessels was not dilated, they contained shaped blood elements

Stomach. The mucosa of the fundus and pyloric part of the stomach was examined. The presence of mucosa, submucosal base, muscle and serous membranes was noted. The stomach was lined with a single-layer prismatic epithelium, under which was located its own plate of the mucous membrane with numerous gastric glands. Violations of the integrity of the surface epithelial layer, as well as dystrophic changes and violations of epithelial cell secretion were not detected. The submucosal base was formed by connective tissue, moderately full-blooded, sometimes rare white blood cells were noted in it. The muscular membrane of the stomach was represented by circular and longitudinal layers. The mucous membrane of the iron-free part of the stomach was lined with multilayered squamous epithelium, no cell changes were observed.

There were no defects in the multi-layered flat non-keratinizing epithelium ***of the esophageal mucosa***.

The wall ***of the small intestine*** consists of mucosa, submucosa, muscle and serous membranes. The mucous membrane consists of the epithelium, its own plate and the muscle layer. The inner plate of the small intestinal mucosa was moderately infiltrated by polymorphonuclear cells with a predominance of lymphocytes. The surface of the mucous membrane formed villi, folds and crypts. The villi were covered with a single-layered cylindrical epithelium, in which edged and goblet-shaped cells differentiated. The submucosa of the small

intestine was formed by loose fibrous connective tissue, slightly infiltrated by polymorphonuclear cells, and was full-blooded. The muscular membrane of the small intestine was represented by circular and longitudinal layers.

The epithelium ***of the large intestine*** was single-row cylindrical. The apical surface of the crypts was lined with edged epithelial cells. The lateral surface of the crypts contained actively secreting, goblet-shaped cells. Lymphoid follicles were located in the submucosal base of the colon. Accumulations of lymphoid tissue had a rounded or oval shape, spread up to the epithelial layer, and consisted mainly of evenly distributed lymphocytes. The blood vessels of the large intestine had wide lumens, contain shaped blood elements. The muscle layers (circular and longitudinal) were thin, and the vegetative ganglia were evenly distributed between them.

The lobular structure ***of the pancreas*** was preserved. The gland was covered with a thin layer of connective tissue. Most of the parenchyma was represented by acinuses formed by a single layer of glandulocytes lying on the basement membrane. Broad basal sections of glandulocytes were intensively stained with hematoxylin and contained rounded nuclei. In the apical part, the cytoplasm was more acidophilic, and granules of secretions were often seen. In part of the glandulocytes, the apical part of the cells contained optically transparent vacuoles. Acinus lumen was small, often almost not visualized. The mucosa of large ducts was formed by a single-layer prismatic epithelium. In the thickness of the gland, Langerhans islets were located in the form of compact groups of light polygonal cells, the size of the islets varied.

Testes. From the surface, the testes were limited by a dense connective tissue membrane, from which thin partitions extended. The connective tissue septa contained clusters of large Leydig cells. The spermatic tubule lumen was unevenly dilated. On the basement membrane there was a layer of spermatogonia alternating with Sertoli support cells. In the direction of the lumen of the tubules, successive stages of maturation of spermatocytes were differentiated, ending with the appearance of spermatozoa.

The ovaries. The organ was topographically divided into cortical and medullary matter. In the root section of the experimental animals, numerous follicles could be traced, which were at different stages of maturation. Почему контрольных, а не экспериментальных?

The thymus retained a pronounced lobular structure. The lobes of the thymus were covered with a connective tissue capsule, which had processes towards the parenchyma, which formed septa. In the thymus, the cortical and brain matter differentiated, and the boundary between them was indistinct. The cortical substance was represented by evenly distributed lymphocytes, lymphoblasts and dendritic epithelial cells. The epithelial cells were large and pale with distinct basophilic nuclei. The capillaries of the cortical substance were filled with red blood cells. Individual gassal corpuscles of immature type were visible in the medulla.

Conclusion on the study of chronic toxicity in rabbits

No animal deaths were recorded during the experiment. The use of Nano-SOD1 for 28 days did not lead to changes in integral indicators (behavior, appearance, body weight, food and water consumption), data from clinical and biochemical analysis of blood, urine, and ECG indicators.

During macroscopic examination and after statistical processing of the mass of internal organs of animals, no deviations were found relative to the control groups of rabbits.

Ophthalmological parameters (biomicroscopy, fundus condition, pupillometry, intraocular pressure) of the experimental groups did not differ from the control ones.

Histological studies did not show any pathological abnormalities in the structure of the internal organs of rabbits.

Topical irritant treatment

Evaluation of the local irritant effect of Nano-SOD1 was performed by macroscopic (rats and rabbits) and histological analysis (rabbits) of the eyes after 28-day instillations into the conjunctival sac of the animals ' eyes.

Macroscopic examination of the eyes

The irritant effect was assessed by the state of the cornea (presence/absence: opacity, lesion), iris (presence/absence: reaction to light, swelling, vascular injection,), conjunctiva (presence/absence: vascular injection, edema, discharge).

No pathology was detected in any animal.

Histological studies

At the end of the experiment, the eyes of rabbits were enucleated, fixed in 10-15% neutral formalin, and embedded in paraffin. Sections were stained with hematoxylin-eosin.

Histological specimens were examined by light microscopy using a Leica DM1000 biological microscope (Leica Microsystems CSC GmbH, Germany).

The cornea, stroma, and endothelium were unchanged. The cells had a regular hexagonal shape and a rounded core, and they fit snugly together. The front camera angle was open. The vascular pattern of the iris and its processes was not changed; there was no edema of the stroma. The ciliary body was without features. The lens structure was normal. Clearly defined nuclear layers of the retina, no dystrophic and or inflammatory phenomena were found. Choroid without special features.

In control and experimental animals treated with 0.04 ml/kg of the test substance, the histological picture was similar. The outer and inner layers of the cornea were clearly visible. The cornea's own substance was formed by a dense decorated fibrous connective tissue. Collagen fibrils were located parallel to the surface of the cornea. There were different types of fibroblasts (flat, process), nerve fibers, and amorphous matter.

In the experimental groups that received a dose of 0.15 mg/kg of the test substance. All layers of the cornea were preserved. Its own substance was also a wide fibrous layer consisting of convoluted plates formed by collagen fibers. It was noted that the inter-fiber space was increased in comparison with the control group and the group receiving 0.04 ml/kg.

Conclusion

Nano-SOD1 did not irritate the eye with repeated instillations into the conjunctival sac of the eyes of animals.

4. Mutagenic properties of Nano-SOD1

Bacteria. A set of indicator strains *of Salmonella typhimurium*, which allows registering mutations such as shifting the reading frame of the genetic code (TA 98 and TA 97) and replacing base pairs (TA 100).

Mice. Achieving this goal is possible only with the use of experimental animals. The number of animals involved in the study was sufficient for complete recording of the studied effects and statistical processing of the obtained data. The females were not pregnant or in labor.

Test Ames

The mutation test *for Salmonella typhimurium* is a bacterial test system for accounting for mutations to prototrophy by histidine under the action of chemical compounds and/or their metabolites that induce mutations such as base replacement or a shift in the reading frame in the genome of this organism.

This method is designed to detect the ability of pharmacological substances or their metabolites to induce gene mutations in indicator strains *of Salmonella typhimurium*.

The bacteria were treated with the test NanoSOD1. After incubation for a certain period of time, the number of revertant colonies in groups of test strains was calculated. The average values, according to the indicator "number of revertant colonies", for groups, treated with the drug, were compared simultaneously with the corresponding groups of negative and positive controls.

If the drug and/or its metabolites have mutagenic activity, they will induce reverse mutations from auxotrophy to prototrophy for histidine in histidine-dependent strains *of Salmonella typhimurium*.

Metabolic activation

For metabolic activation, the S9 fraction of the liver of Wistar rats was used.

Male rats were given sovol (commercial mixture of polychlorinated biphenyls) intraperitoneally at a dose of 300 mg/kg 5 days before slaughter. A day before the release of the homogenate, the rats were kept on a starvation diet, they received only water. Euthanasia was performed by decapitation.

In an animal under aseptic conditions, the liver was removed, weighed, and washed with a sterile chilled solution of 0.15 M potassium chloride. The liver was crushed in a chemical beaker with scissors, and then in a glass homogenizer with a ground Teflon pestle in a three-fold volume of 0.15 M potassium chloride. All manipulations were performed on ice. The resulting

homogenate was centrifuged in a centrifuge with cooling (0-4 ^{0}S) at 9000 g for 15 minutes. The supernatant (S9 fraction) was bottled and stored in the freezer at -80 °C until use.

When preparing the microsomal activating mixture, the S9 fraction was thawed at room temperature and placed on ice. 1 ml of the mixture should contain 0.1-0.3 ml of the S9 fraction, normalized by protein content (4 mg per dish), 4 mM NADP, 5 mM glucose-6-phosphate, 33 mM KCl, 8 mM $MgCl_2$. This composition was adjusted to 1 ml with 0.1 M phosphate buffer (pH 7.4).

Studied concentrations

The dosage form Nano-SOD1 was tested in five concentrations by adding 0.1 ml of the initial solution to a Petri dish and four consecutive 10-fold dilutions in sterile distilled water.

Controls

Substances that induce mutations in the corresponding indicator strains were used as a positive control. For variants without a metabolic activation system: for strain TA 100-sodium azide, 10 mkg per dish; for strain TA 98-2-nitrofluaren (2NF), 5 mkg per dish; for strain TA 97-9-aminoacridine (9AA), 50 mkg per dish. To control the activity of the metabolic activation, ethidium bromide, 10 micrograms per dish, was used on the TA 98 strain at CM+.

Distilled water (0.1 ml per dish) was used as a negative control.

Experiment

The mutagenic activity of Nano-SOD1 was studied in a modification with preincubation. In centrifuge tubes containing 0.1 ml of bacterial culture in 0.5 ml of buffer or microsomal activating mixture (in the CM+ variant), 0.1 ml of the corresponding dilutions of the drug were added. The mixture was incubated for 2 hours at a temperature of 37^{0}C and intensive shaking.

Selective semi-enriched agar 0.7% (2 ml each) in test tubes was placed in a thermostatically controlled water bath at 46^{0}C.

At the end of preincubation, the contents of the centrifuge tubes were pipetted into tubes with 0.7% semi-liquid agar, quickly mixed and applied to a layer of lower 1.5% agar on Petri dishes.

The dishes were kept at room temperature for 30-40 minutes and after the agar had completely solidified, they were transferred to the thermostat (37 ^{0}C). The results were recorded after 48 hours of incubation.

The experiment included variants without the metabolic activation system (CM-) and in its presence (CM+). In the CM-variants, the action of direct mutagens is recorded, that is, substances that exhibit a mutagenic effect due to the activity of the initial structure of the substance. The effect of indirect mutagens (promutagens) - compounds whose effect is associated with the formation of mutagenic metabolites-can be taken into account when

comparing the results obtained in the CM – and CM+ variants.

In each control and experimental variants of the experiment, 2 dishes were used.

Test for accounting for chromosomal aberrations.

Method of administration, choice of doses and preparation of the drug for administration.

Calculation of the therapeutic dose for a person

In clinical practice, Nano-SOD1 is supposed to be instilled 40 microliters in each eye 3 times a day. The volume of the drug administered will be 240 microliters (0.24 ml). For a person with an average body weight of 70 kg, the dose will be 0.003 ml/kg (0.24 ml/70 kg=0.003 ml/kg).

Selection of doses for intraperitoneal administration

2 doses of Nano-SOD1 were used:

- 0.03535 ml/kg-therapeutic dose TD based on dose conversion from human to mouse (0.003 ml/kg*11.8 (conversion factor for mouse) = 0.035 ml/kg);
- 22.0 ml/ kg (maximum volume that can be injected intraperitoneally into the mouse) - subtoxic dose of TDx570. This dose was used because, due to the low toxicity of the drug, it was impossible to calculate LD_{50}.

Control animals were injected with PBS (10 mM sodium-phosphate buffer, pH 7.4, 150 mm NaCl) in a volume of 20 ml/kg.

Selection of doses for instillation into the conjunctival sac of the eye

When Nano-SOD1was instilled in mice,a subtoxic dose (maximum possible volume) was instilled into the conjunctival SAC of the eye) – 1 drop (10 µl) in each eye. The dose averaged 0.8 ml/kg and exceeded the therapeutic dose (350.035 mg/kg for a mouse) by more than 20 times.

Research design

The method is based on the registration of micronuclei in mammalian polychromatophilic erythrocytes. Bone marrow cells are characterized by a high level of mitotic activity. If mutagenic activity is detected, the frequency of cells with micronuclei in the groups treated with Nano-SOD1will be statistically significantly higher than in the control groups of animals. On average, the frequency of cells with micronuclei in healthy intact mice is 0.2%.

Nano-SOD1 was administered as follows:

– with four times intraperitoneal administration in the highest therapeutic dose with an interval of 24 hours to males and females, preparation of drugs 24 hours after the last administration;

– with a single intraperitoneal injection in the highest therapeutic dose to males, preparation of drugs 24 hours after administration;
– once to males by intraperitoneal administration in a subtoxic dose corresponding to 570 higher therapeutic doses, preparation of drugs 24 hours after administration;
– for four-time instillations into the conjunctival SAC of the eye in males and females in a subtoxic dose corresponding to more than 20 higher therapeutic doses, preparation of drugs 24 hours after the last administration.

Groups of animals, controls, doses and method of treatment of animals are indicated in Table S66.A 10 mM sodium-phosphate buffer, pH 7.4, containing 150 mm NaCl (PBS) was used as negative controls.

As a positive control, a cyclophosphane solution was used. Cyclophosphamide prepared ex tempore was administered to males intraperitoneally once at a dose of 10 mg/kg 24 hours before euthanasia.

Table S66. Study design of the chromosomal aberration accounting test.

Groups	Characteristics of mice	Number of animals	Study drug	Introduction mode
1	F1 mice (CBAxC57Bl/6), males	6	PBS (negative control)	Four times with an interval of 24 hours intraperitoneally, receiving drugs 24 hours after the last administration
2	F1 mice (CBAxC57Bl/6), females	6	PBS (negative control)	Four times with an interval of 24 hours intraperitoneally, receiving drugs 24 hours after the last injection
3	F1 mice (CBAxC57Bl/6), males	6	Cyclophosphamide (positive control)	Intraperitoneally, once, receiving drugs 24 hours after administration
4	F1 mice (CBAxC57Bl/6), males	6	Nano-SOD1	Recommended therapeutic dose, single intraperitoneal injection, receiving drugs 24 hours after administration
5	F1 mice (CBAxC57Bl/6), males	6	Nano-SOD1	Subtoxic dose once intraperitoneally, receiving drugs 24 hours after administration
6	F1 mice (CBAxC57Bl/6), males	6	Nano-SOD1	Recommended therapeutic dose, four times with an interval of 24 hours intraperitoneally, receiving drugs 24 hours after administration
7	F1 mice (CBAxC57Bl/6), females	6	Nano-SOD1	Recommended therapeutic dose, four times with an interval of 24 hours intraperitoneally, receiving drugs 24 hours after administration
8	F1 mice (CBAxC57Bl/6), males	6	Nano-SOD1	Subtoxic dose of four-fold instillation into the conjunctival SAC of the eye, receiving drugs 24 hours after administration

Groups	Characteristics of mice	Number of animals	Study drug	Introduction mode
9	F1 mice (CBAxC57Bl/6), females	6	Nano-SOD1	Subtoxic dose of four-fold instillation into the conjunctival SAC of the eye, receiving drugs 24 hours after administration

Results of the Ames test

The results of the experiment using indicator strains *of Salmonella typhimurium* (TA 100, TA 98 and TA 97) are presented in Tables S67-69. In all Tables $X1_1$ и X_2 and x2 - the number of revertant colonies per 1 and 2 dishes; X_{AV} - the average number of revertant colonies per dish; $X_{AV,exp}/X_{AV,cont}$ - the ratio of the average number of revertant colonies per dish in the experiment to that in the control.

The number of revertant colonies in the control with solvent in the CM - and CM+ variants was within the range of spontaneous level fluctuations for these strains. The response of the strains to standard mutagens was within normal levels.

In accordance with generally accepted approaches, the mutagenic effect was considered significant if the average number of revertant colonies per Cup in the experimental version exceeded that in the control version by 2 or more times.

The Nano-SOD1 in all tested concentrations did not show a mutagenic effect on strains TA 100, TA 98 and TA 97 in variants without and in the presence of a metabolic activation system.

Table S67. Results of evaluation of the mutagenic activity of Nano-SOD1 in the test Ames on the TA 100 strain.

Metabolic system activation	Drug concentration, 0.1 ml/dish	number of revertant colonies per dish			$X_{AV,exp}/X_{AV,cont}$
		X_1	X_2	X_{CP}	
CM-	Source	78	104	91.0	0.74
	1:10	115	136	125.5	1.02
	1:100	103	123	113.0	0.92
	1:1000	122	131	126.5	1.03
	1:10000	131	111	121.0	0.98
	H_2O	120	126	123.0	
	Sodium azide	>1000	>1000	>1000.0	>8.13*
CM+	Source	70	77	73.5	0.62
	1:10	117	124	120.5	1.01
	1:100	102	113	107.5	0.90
	1:1000	128	112	120.0	1.00
	1:10000	107	120	113.5	0.95
	H_2O.	116	123	119.5	

* - significant mutagenic effect (sodium azide,2NF, 9AA and ethidium bromide-standard mutagens (positive control)

Table S68. Results of evaluation of the mutagenic activity of Nano-SOD1 in the test Ames on the TA 98 strain.

Metabolic system activation	Drug concentration, 0.1 ml/h	number of revertant colonies per dish			$X_{AV,exp}/X_{AV,cont}$
		X_1	X_2	X_{CP}	
CM-	Source	11	9	10.0	0.59
	1:10	23	16	19.5	1.15
	1:100	19	25	22.0	1.29
	1:1000	18	20	19.0	1.12
	1:10000	15	18	16.5	0.97
	H_2O.	19	15	17.0	
	DMSO	21	20	20.5	
	2NF	152	139	145.5	7.10*
CM+	Source	9	12	10.5	0.54
	1:10	23	20	21.5	1.10
	1:100	21	18	19.5	1.00
	1:1000	18	22	20.0	1.03
	1:10000	13	15	14.0	0.72
	H_2O	17	22	19.5	
	Ethidium bromide	436	312	374.0	19.18*

* - significant mutagenic effect (sodium azide,2NF, 9AA and ethidium bromide-standard mutagens (positive control)

Table S69. Results of evaluation of the mutagenic activity of Nano-SOD1 in the test Ames on the TA 97 strain.

Metabolic system activation	Drug concentration, 0.1 ml/h	number of revertant colonies per dish			$X_{AV,exp}/X_{AV,cont}$
		X_1	X_2	X_{CP}	
CM-	Source	112	115	113.5	0.89
	1:10	131	120	125.5	0.99
	1:100	154	137	145.5	1.15
	1:1000	121	122	121.5	0.96
	1:10000	133	140	136.5	1.07
	H2O	126	128	127.0	
	DMSO	134	125	129.5	
	9AA	714	638	676.0	5.22*
CM+	Source	109	118	113.5	0.88
	1:10	145	163	154.0	1.20
	1:100	154	137	145.5	1.13
	1:1000	129	123	126.0	0.98
	1:10000	112	143	127.5	0.99
	H2O	133	124	128.5	

* - significant mutagenic effect (sodium azide,2NF, 9AA and ethidium bromide-standard mutagens (positive control)

The Nano-SOD1 in five concentrations (the initial solution and its dilutions by 10, 100, 1000 and 10000 times) did not induce gene mutations *in the test strains of Salmonella typhimurium* TA 100, TA 98, and TA 97 without and in the presence of a metabolic activation system.

Results of the test accounting for chromosomal aberrations

The samples were analyzed by light microscopy using a Leica DM1000 microscope (Leica Microsystems CSC GmbH, Germany).

2000 polychromatophilic erythrocytes (PCR) from each animal were analyzed to account for PCR with micronuclei. The proportion of PHE from the sum of PHE and normochromic red blood cells was determined with an additional count of 400 red blood cells.

The preparations were decoded at the end of microscopic analysis of the material obtained for each animal and for each group of animals.

Statistical processing of the results for accounting for micronuclei was performed separately for males and females by comparing the experimental groups with the control groups using the criterion χ^2 at a significance level for each of the comparisons of 0.05.

The criterion for a positive result is a statistically significant increase in the number of PCRs with micronuclei in at least one of the experimental groups compared to the control group. The obtained positive result indicates that the substance induces chromosome breaks and/or

disorders of the mitotic apparatus of cells in experimental animals, which are detected as micronuclei in interphase.

Comparison of the proportion of PCR from the sum of all red blood cells in the control and experimental groups was carried out using the Mann-Whitney test.

The results of the analysis of the frequency of polychromatophilic erythrocytes (PE) with micronuclei (MN) and the frequency of PE from the sum of all red blood cells (poly - and normochromic) in the bone marrow after the use of Nano-SOD1for each group of animals are presented in Table S70.

The frequency of PCR with micronuclei in control animals after intraperitoneal administration of 10 mM sodium-phosphate buffer, pH 7.4, containing 150 mm NaCl (PBS) was 1.2% for males and 1,1% for females, which corresponds to the accumulated laboratory control and the level noted in the literature - up to 2%.

Statistically significant differences in the frequency of PCR with micronuclei was not revealed when comparing groups of male mice treated with Nano-SOD1 (groups 4, 5, 6 and 8) with a control group of males (group 1), and also groups of females treated with Nano-SOD! (groups 7 and 9) with a control group of females (group 2).

Cyclophosphamide (positive control, group 3) at a dose of 10 mg/kg induced 7.2% PCR with micronuclei, which is statistically significantly higher than in the negative control group and in other experimental groups (P< 0.05).

The Nano-SOD1 in the studied doses and modes of exposure did not affect the frequency of polychromatophilic red blood cells in the bone marrow of mice.

Table S70. Results of recording micronuclei in polychromatophilic erythrocytes of mouse bone marrow after exposure to Nano-SOD1, as well as in positive and negative controls.

Group	Number of cells (examined)	Number of PCRs with MNs per 1000 PCRs	Share PCR from the sum of all red blood cells
1	12000	1.23±0.28	0.53±0.01
2	12000	1.32±0.30	0.54±0.02
3	12000	7.18±0.63*	0.55±0.02
4	12000	1.17±0.18	0.52±0.01
5	12000	1.33±0.33	0.54±0.02

6	12000	1.25±0.21	0.54±0.01
7	12000	1.27±0.16	0.53±0.02
8	12000	0.65±0.23	0.54±0.02
9	12000	0.68±0.25	0.54±0.01

The Nano-SOD1 did not show cytogenetic activity in the test for accounting for micronuclei in polychromatophilic erythrocytes of mouse bone marrow when administered intraperitoneally at the highest therapeutic dose for mice (0.035 ml/kg) and at a dose exceeding the therapeutic one by 570 times (20 ml/kg). Mutagenic activity was also not detected under the conditions of four-fold instillations into the conjunctival sac of the eyes of male and female mice at a dose exceeding the therapeutic dose by more than в 22.0 times.

Thus, the test of accounting for micronuclei in polychromatophilic erythrocytes of mouse bone marrow showed the absence of mutagenic activity of Nano-SOD1 both in the range of recommended doses and under the action of sub-toxic doses.

5. *Allergenic properties of Nano-SOD1*

Guinea pigs. The therapeutic dose of Nano-SOD1 for Guinea pigs, considering the conversion of doses from person to pig, will be 0.014 ml/kg (0.003 ml/kg*4.7 (conversion factor for pigs) = 0.014 ml/kg).

Animals were instilled in the conjunctival sac of the eye with a therapeutic dose an order of magnitude higher (TDx10).

Evaluating of allergenic properties

Evaluation of anaphylactogenic activity in the study of the general anaphylaxis response

The anaphylactogenic activity of Nano-SOD1 was studied in albino Guinea pigs in comparison with negative and positive controls. A solvent of 10 mM sodium phosphate buffer, pH 7.4, containing 150 mm NaCl (PBS) was used as a negative control, and 1% chicken egg protein was used as a positive control.

The animals were divided into groups by random sampling. The body weight was taken as a criterion.

Group 1-negative control, PBS;

Group 2-Nano-SOD1, TD;

Group 3-Nano-SOD1, TDx10;

Group 4 – positive control, 1% chicken egg protein.

The drug and control substances were administered according to the following scheme: the first injection was subcutaneously; the next two were administered intramuscularly. Permissive injection with Nano-SOD1 was performed endocardially on day 21. The resolution dose was equal to the total sensitizing dose. A permissive dose was also administered to control animals receiving the solvent. Pigs from the positive control group were given intracardiac chicken egg protein at a dose of 1 mg per 300 g of body weight. Accounting for the intensity of anaphylactic shock (anaphylactic index) was evaluated in Weigle indices:

$$\frac{(Nx4) + (N1x3) + (N2x2) + (N3x1) + (N4x0)}{N + N1 + N2 + N3 + N4}$$

where N is the number of Guinea pigs that have died;

N1 — number of Guinea pigs that developed severe shock;

N2 — number of Guinea pigs that have developed moderate shock;

N3 — number of Guinea pigs that developed mild shock;

N4-Guinea pigs that did not go into shock.

If all animals in the group die, the Weigle index will be 4 (++++). In severe shock-3 (+++), in moderate shock — 2 (++), in mild shock — 1 (+), in the absence of anaphylactoid reactions in Guinea pigs — the index is 0.

Conjunctival test

Conjunctival testing was performed on albino Guinea pigs in comparison with negative and positive controls. A solvent of 10 mM sodium – phosphate buffer, pH 7.4, containing 150 mm NaCl (PBS) was used as a negative control, and 1% chicken egg protein was used as a positive control.

The animals were divided into groups by random sampling. The body weight was taken as a criterion. 10 animals were selected for each group:

Group 1-negative control, PBS;

Group 2-Nano-SOD1, TD;

Group3-Nano-SOD1, TDx10;

Group 4 – positive control, 1% chicken egg protein.

Nano-SOD1 and control substances were instilled into the conjunctival sac of the eyes daily for 14 days. After the last instillation, a provocative test was performed a day later. Experimental guinea pigs and pigs treated with PBS, were instilled with the test drug under the upper eyelid of the right eye, and water was injected into the left eye (control). Guinea pigs from the positive control group were instilled with 1% chicken egg protein in their right eye. The

reaction was observed after 1-5 minutes (immediate reaction), and 24–24-48 hours (delayed hypersensitivity) and evaluated according to the following scale in points:

1-slight redness of the tear duct;

2 - redness of the tear duct and sclera in the direction to the cornea;

3 – redness of the entire conjunctiva and sclera; the reaction is accompanied by itching and purulent ophthalmitis.

Mast cell degranulation reaction

Evaluation of the mast cell degranulation reaction was performed on the blood serum of sensitized albino Guinea pigs after the conjunctival test reaction.

To obtain mast cells, an intact rat was clogged with bloodletting. Intraperitoneally injected heated to 37^0C solution Tirode. After a light massage, the abdominal wall was incised along the midline and exudate was collected in a centrifuge tube.

Preparations were prepared on slides stained with 0.3% alcohol solution of neutral red. 0.03 ml of the experimental animal's serum and 0.03 ml of the test preparation Nano-SOD1 were added to 0.03 ml of the mast cell suspension Nano-SOD1. After 15 minutes of incubation of hermetically sealed preparations, they were microscopized under X40 magnification. Normal and degranulated mast cells were counted (a total of 100). The reaction was considered positive if the degree of degranulation exceeded 20%.

Results of the study of allergizing effect

Assessment of the General anaphylaxis response

The results of the study of the anaphylactogenic activity of Nano-SOD1 in experiments on Guinea pigs are presented in Table S71. The data of the study show that intracardiac administration of Nano-SOD1 to animals sensitized by a ten-fold therapeutic dose resulted in moderate shock in the 1st Guinea pig (and the reaction index was 0.2), which can be considered as a manifestation of individual sensitivity. No reaction to the drug was detected during intracardiac administration of Nano-SOD1 to unsensitized pigs and pigs that received a therapeutic dose of the drug during sensitization (reaction index - 0). In pigs sensitized with chicken egg protein, the reaction index was 1.2.

Table S71. Indicators of anaphylactic reaction of Guinea pigs.

Drug, dose	Number of animals in group	the number of animals with reaction					
		++++	+++	++	+	-	Anafilactic index
Negative control	10					10	0
Nano-SOD1, TD	10					10	0
Nano-SOD1, TDx10	10			1		9	0.2
Positive control	10		1	2	5	2	1.2

Conjunctival test

The results of the conjunctival test are shown in Table S72. A provocative test after conjunctival instillations of Nano-SOD1 and PBS for 14 days did not reveal either immediate or delayed hypersensitivity in Guinea pigs. The use of 1% chicken egg protein caused mild irritation.

Table S72. Indicators of the conjunctival test, points.

Duration of the study	Groups			
	Negative control	Nano-SOD1		Positive control
		TD	TDx10	
15 minutes	0.0±0.00	0.0±0.00	0.0±0.00	0.7±0.21*
24 hours	0.0±0.00	0.0±0.00	0.0±0.00	0.6±0.27*
48 hours	0.0±0.00	0.0±0.00	0.0±0.00	0.5±0.22*

* - the difference in comparison with the negative control is significant according to the t-student's t criterion (p<0.05)

Mast cell degranulation reaction

Table S73 shows the results of mast cell counts. The percentage of degranulating mast cells mixed with the blood serum of Guinea pigs treated with the drug Nano-SOD1, both at the therapeutic dose and 10 times незначительноhigher, increases slightly, but does not exceed the 20% level, which allows us to consider the reaction negative.

Table S73. Indicators of mast cell degranulation (%).

Indicator	Groups			
	Negative Control	Nano-SOD1		Positive control
		TD	TDx10	
Normal cells	97.5±0.50	96.8±0.70	96.8±0.66	93.2±0.90*
Degranulated cells	2.5±0.50	3.2±0.70	3.2±0.66	6.8±0.90*
% degranulations	2.5	3.2	3.2	6.8

* - the difference in comparison with the negative control is significant according to the t-student's criterion (p<0.05)

6. *Immunotoxic properties of Nano-SOD1*

Mice. The therapeutic dose of Nano-SOD1 for a mouse will be 0.035 ml/kg (0.003 ml/kg*11.8 (conversion factor for mice) = 0.035 ml/kg).

For the study of immunotoxicity in animals, the use of 2 doses is recommended: ten-fold and a hundred-fold therapeutic. Because it was impossible to instillate a hundred-fold dose of Nano-SOD1 into the conjunctival sac of the eye, an increase in the dose was achieved by multiple instillations.

Animals were instilled in the conjunctival sac of the eye with a double therapeutic dose of 0.07 ml/kg (TDx2) and an order of magnitude higher 0.7 ml/kg (TDx20) daily for 14 days.

Immunotoxicity was evaluated in male mice. Distribution into groups was carried out by random sampling. In addition, similar groups of control animals were formed, which were injected with a solvent – PBS (10 mM sodium-phosphate buffer, pH 7.4, containing 150 mm NaCl) in the same way in a volume of 0.8 ml/kg.

Group 1- control;

Group 2 -Nano-SOD1, TDx2;

Group 3 -Nano-SOD1, TDx20

Immunotoxic effect assessment program

To study the immunotoxic effect of Nano-SOD1, standard research methods were used:

- Humoral immune response

Evaluation of the humoral immune response, i.e. the ability of the immune system to produce antibodies in response to a non-infectious antigen, was performed in the hemagglutination reaction. Sheep erythrocytes were used as the antigen.

Nano-SOD1 was instilled into the conjunctival sac of both eyes for 14 days in the animals of the experimental groups and PBS was instilled in the control animals. At the end of administration, suspension of sheep red blood cells (ES) washed three times in sterile isotonic sodium chloride solution at a suboptimal dose of 5 x10^7 ES/mice were injected intraperitoneally. 7 days after immunization, the animals were euthanized by decapitation, and blood was collected. Serum from intact animals was used to control the formation of antibodies to the introduced antigen. To determine the hemagglutinin titer, a saline solution (100 μl) was applied to the wells of microplates. The same amount of serum was added to the first well and a special dispenser was used to transfer the diluted sample from one well to another after mixing with saline solution. Then a micropipet was added to each well ES in a concentration of 2x10^8 cells. 100 μl of saline solution was added to the control well. The plates were gently shaken and placed

in a thermostat for 2 hours at 37 °C. Antibody Titer was log_2T, where T is the number of the last dilution of blood serum at which hemagglutination of red blood cells occurs.

– Cellular immune response

Cellular immunity was assessed using a delayed-type hypersensitivity reaction (HRT) when hapten – trinitrobenzosulfonic acid (TNBS) was administered. The mechanism of induction of delayed hypersensitivity with the introduction of this hapten is like that with the development of contact allergies to many chemical and medicinal substances that form complexes with body proteins. An increase in the severity of this reaction under the influence of the tested pharmacological agent can also characterize the risk of changing the allergostatus and the possibility of increasing the body's sensitivity to traditional allergens.

Tests were performed on hybrid mice (CBA x C57BL/6) F_1. Animals of the experimental groups were instilled Nano-SOD1 into the conjunctival sac of both eyes for 14 days, and control animals were instilled with PBS. After the last administration, mice were immunized with a 10 mM solution of trinitrobenzosulfonic acid (TNBS) in a volume of 0.2 ml at the base of the tail. The second injection of TNBS (50 μl) was performed on day 6 in the right hind paw pad. 50 μl of sterile isotonic sodium chloride solution was injected into the left paw. After 24 hours, the mass of both paws was determined and the reaction index (IR) was calculated for each animal according to the formula: $IR = (M_o – M_K)/M_K*100\%$, where M_o and M_K are the masses of the experimental and control paws. Statistical significance of differences between the control and the experiment was assessed by the Student's t-test.

- Study of phagocytic activity of peritoneal macrophages

The reaction was performed in hybrid mice (CBAxC57BL/6)F_1. Nano-SOD1 was instilled into the conjunctival sac of both eyes for 14 days in the animals of the experimental groups and PBS was instilled in the control animals. At the end of administration, 24 hours later, mice were slaughtered using cervical dislocation. Mice were aseptically injected intraperitoneally with 5 ml of medium 199 containing 20% FBS (fetal bovine serum). After massage of the abdominal band, the medium was taken with a syringe. Portions of the cell suspension obtained from the entire group of animals were combined into a common pool. The concentration of live cells was increased to $1.5*10^3$ cells/ml. Cell suspension was sterile poured 2 ml each into plastic Petri dishes with a diameter of 40 mm. The dishes were incubated for 2 hours at 37^0C. The supernatant containing non-adherent cells was drained, and the monolayer fixed on the plastic was washed twice with medium 199. A solution containing neutral red was poured into slightly dried dishes and kept at 37^0C for 1 hour. The solution was drained. The cells were washed with medium 199. 3 ml of the lyzing solution was added to each dishes. The solution was drained into

test tubes and the optical density was measured on a spectrophotometer at a wavelength of 540 nm. The results were obtained in conventional units expressing the optical density of the lysate.

Results of the immunotoxicity study

The results of the study are shown in Table S74. As follows from the data given in the Table, a single intraperitoneal injection of ES induced the formation of antibodies to the introduced antigen in them. Administration of Nano-SOD1 into the conjunctival sac of the eyes for 14 days, both at twice the therapeutic dose and в 20 times its excess, did not affect antibody genesis. The serum hemagglutinin titers of mice treated with different doses of the test drug did not differ significantly from those in the control group.

Table S74. Antibody titer in ES-immunized mice (% , M±m).

Animal group	Antibody titer
Intact	0.0±0.00
Control	8.0±0.21
Nano-SOD1, TDx2	8.1±0.23
Nano-SOD1, TDx20	8.2±0.20

This reaction is the main indicator of the formation of a humoral immune response, and in accordance with this, the results obtained allow us to conclude that Nano-SOD1did not affect the humoral immune response.

Delayed hypersensitivity reaction to TBSK

The results of evaluating the effect of Nano-SOD1 on the development of a cellular immune response to hapten (TNBS) are presented in Table S75.

Table S75. Results of the effect of Nano-SOD1 on the parameters of the cellular immune response in mice (M±m).

Feet	Weight of legs, g		
	Control	Nano-SOD1, TDx2	Nano-SOD1, TDx20
L	0.138±0.0034	0.144±0.0024	0.144±0.0022
R	0.139±0.0032	0.145±0.0023	0.145±0.0019
IR	0.983±0.3139	0.919±0.2809	0.996±0.3260

L-control foot

R-experimental foot

IR-reaction index, L/R

The use of the test drug in the form of instillations into the conjunctival sac of the eyes of mice, both at twice the therapeutic dose and 20 times its maximum, did not induce a delayed hypersensitivity reaction to hapten (trinitrobenzo-sulfonic acid).

Phagocytic activity of peritoneal macrophages

The results of the effect of Nano-SOD1 on the phagocytic activity of macrophages are presented in Table S76.

Table S76. Optical density of lysate of peritoneal exudate cells (M±m).

Animal group	Optical density, conl .units.
Control	0.655±0.0108
Nano-SOD1, TDx2	0.657±0.0069
Nano-SOD1, TDx20	0.898±0.0367*

* - the difference in comparison with the negative control is significant according to the t-student's criterion ($p<0.05$)

Repeated administration of Nano-SOD1 to mice in a 20-fold therapeutic dose moderately increased the phagocytic activity of macrophages.

7. *Generative toxicity study*

Research design. Nano-SOD1 was instilled daily into the conjunctival sac of the eye: males for 48 days (spermatogenesis period), females for 15 days (3 estral cycles). The drug was used in two doses: TD and TDx10. Intact animals were used as controls.

Then the experimental animals were paired with the control animals, forming groups:

Group 1-control males (14 animals) + control females(14 animals)

Group 2-control males (14 animals) + females nano-SOD1 TD (14 animals)

Group 3-control females (14 animals) + males nano-SOD1 TD (14 animals)

Group 4-control males (14 animals) + females nano-SOD1 TDx10 (14 animals)

Group 5-control females (14 animals) + males nano-SOD1 TDx10 (14 animals)

Males were joined by females in a 1: 2 ratio for 10 days (2 estrous cycles). Fertilization was recorded using vaginal smears. The presence of spermatozoa in smears was an indicator of female fertilization.

Half of the pregnant females were euthanized by cervical dislocation on the 20th day of pregnancy. After euthanasia, the number of live and dead fetuses was counted, weighed, cranio-caudal size was measured; the number of yellow bodies in the ovaries was counted, and the number of implantation and resorption sites in the uterus was calculated. Based on the data, we determined the indicators of pre-and post-implantation death, calculated the fertility index and pregnancy index. The state of reproductive function of males was judged by the results of a study of females paired with males receiving the drug. A part of fetus (about 1/3) was fixed in liquid Boun, 2/3 of the fetuses were fixed in 96% ethanol to study the condition of the skeleton according to the Dawson method.

The other half of the females were left to give birth and the offspring were monitored for a month, recording behavior, body weight dynamics, survival, and physical development.

Part of the animals after the end of instillation Nano-SOD1 was removed from the experiment to study the morphological study of the testes.

Animal observations

Each animal was monitored daily. The examination included: survival rate, appearance, condition of the coat, eyes, nose, breathing pattern, behavior (agitation, aggressiveness), reaction to external stimuli, pain response, food and water consumption, amount and consistency of fecal matter, frequency of urination and color of urine, sexual behavior after cupping. The results of examinations were recorded in laboratory maps.

Recorded indicators of adult animals:

- dynamics of body weight (weighing 1 time per week);

- number and timing of deaths(if any);
- number of pregnant females;
- number of yellow bodies in the ovaries of each euthanized female;
- number of implantation sites for each euthanized female;
- number of resorptions in each euthanized female;
- litter size of each female;
- number of live fetuses in each female;
- number of dead fetuses in each female;
- the presence of spermatogonia, 1st and 2nd order spermatocytes, spermatids and spermatozoa in the testes (counted in 100 tubules);
- average number of normal spermatogonia in each tubule (20 tubules were counted);
- relative number of tubules with the 12th stage of meiosis (counted in 100 tubules).

Recorded indicators of offspring:

- number of fetuses with external developmental abnormalities;
- number of fetuses with anomalies of internal organs (according to the Wilson method);
- number of fetuses with skeletal disorders (according to the Dawson method);
- litter size;
- number of live and dead newborns;
- death of newborns;
- body mass index(4, 7, 14, 21, 28 , 35 day);
- cranio-caudal size;
- sticking of the auricle (from 2 days);
- the appearance of primary hair (from 4 days);
- incisor eruption (from day 6);
- eye opening (from day 12);
- lowering of testicles (from day 25);
- opening of the vagina (from day 30).

Calculated metrics:

- Preimplantation mortality (%) = (number of yellow bodies – number of implantation sites)/number of yellow bodies * 100
- Post-implantation mortality = number of implantation sites – number of live fetuses/number of implantation sites * 100
- Fertility index (%) = number of pregnant females /number of transplanted * 100

- Pregnancy index (%) = number of pregnant females/number of fertilized females * 100
- Offspring survival index (%) = number of surviving pups at the end of the study/number of pups born * 100.
- Index of spermatogenesis = ΣA/100, where A is the number of stages in each tubule, 100 is the number of counted tubules. The spermatogenesis index was calculated using a 4-point system, detectingc the presence of spermatogonia, 1st-and 2nd-order spermatocytes, spermatids, and spermatozoa in the tubule.

Terminal procedures

Euthanasia

Euthanasia of males, females and offspring after the study was performed by decapitation with pre-operative anesthesia. Pregnant females were euthanized by cervical dislocation.

Technique of autopsy of pregnant females

After euthanasia, the abdominal cavity was carefully opened, a palpatory examination of the uterine horns was performed, counting the number of fetuses and resorption sites. The right and left ovaries were isolated, thoroughly cleaned of adipose tissue, and placed on moistened filter paper in a Petri dish. The uterine horns were carefully opened along the outer edge and the fetuses were released from the amniotic membranes, separated from the placenta, while squeezing the umbilical cord for 2-3 seconds with tweezers. The fetus of each litter were carefully examined for external malformations, edema, or hemorrhage. The embryos were then weighed and their cranio-caudal size measured.

The fetuses of each litter were divided into 2 groups. One group (1/3) was fixed in liquid Buena to study the internal organs according to the Wilson method, the other (2/3) - in 96 % ethanol to study the condition of the skeleton according to the Dawson method.

The number of yellow bodies in the ovaries, the number of implantation sites in the uterus, and the number of live, dead, and resorbed fetuses were counted. Based on these data, the level of pre - and post-implantation death of embryos was determined.

Investigation of fetal internal organs by the Wilson method

Fetuses were fixed for 10 days in liquid Buena, consisting of a supersaturated solution of picric acid, 40% formalin and glacial acetic acid in a ratio of 15:5:1, respectively.

For the study, the embryos were cut into 9 sagittal sections, which were carefully examined with a magnifying glass. Incisions were made:

1 parallel to the lower jaw to separate the head from the trunk;

2 perpendicular to the lower jaw just behind the vibrissae. The condition of the lower jaw, anterior hard palate, and nasal cavity was studied;

3 through the middle of the eyeballs. The condition of the eyes and olfactory bulbs was studied;

4 between the 3rd and 5th slices. We studied the state of the brain: the cerebral cortex, lateral and third ventricles;

5 just behind the ears. The condition of the fourth ventricle of the brain, the presence of dropsy of the brain were studied;

6 in front of the forelimbs. The condition of the esophagus, trachea, spinal cord and blood vessels was studied;

7 behind the forelimbs. We studied the organs of the chest cavity: the state of the heart, lungs, bronchi, esophagus, and spinal cord;

8 between the 7th incision and the umbilical ring through the liver. After the examination, the liver was carefully removed and the diaphragm was examined;

9 slightly below the umbilical ring. After careful removal of the intestines and liver, the condition of the kidneys, ureters, bladder, rectum, and genitals was studied.

Detection of fetal skeletal abnormalities by Dawson's method

Fetuses were fixed in 96% alcohol for 15 days. Then, the liver and intestines were removed from each fetus, after which they were filled with 1% KOH solution for 2 days until the tissues became clear and the visible skeleton appeared. After that, the fetuses were washed 3-4 times with tap water and filled for 5 days with alizarin dye, which was prepared according to the prescription of A. P.Dyban: KOH-10 g, glycerin-150 ml, distilled water-800 ml (solution "B") and added a few drops of 1% aqueous solution of red alizarin (solution" A") until a lilac hue was formed. After staining the skeleton in lilac color, the fetuses were washed in solution " B " until the solution and fetus tissues were completely clarified with daily wiring according to the Table S77.

Table S77.

	I	II	III	IV	V
Glycerin	20%	20%	40%	50%	100%
Alcohol	10%	20%	40%	50%	0
Dist. water	70%	60%	20%	0	0

Results of the generative toxicity study

Clinical observations of animals during drug administration

Survival rate

During the study, all the animals remained alive.

Appearance and behavior.

The rats looked healthy, readily ate food, reacted to external stimuli, and showed interest in people. Muscle tone was not characterized by increased excitability. The rats were of average fatness and did not suffer from exhaustion. The coat was thick, smooth and shiny, tightly fitting to the surface of the body, hair loss or brittleness was not detected. The abdominal area was not enlarged in volume. Breathing was smooth, regular rhythm, uncomplicated. Salivation without pathology. The auricles were pink without crusts, not inflamed, twitching was not noticed. Teeth of normal color, no breakdowns were observed. The frequency of urination, urine color, gastrointestinal parameters, muscle tone, reflexes corresponded to the physiological norm. No corneal opacities, lacrimation, or any eye abnormalities were observed. Zoosocial behavior did not differ from that of the control animals.

Dynamics of body weight

Tables S78 and S79 show data on the body weight of rats during the use of Nano-SOD1. At this time, the body weight of the rats showed a positive trend and did not significantly differ between the control and experimental groups.

Table S79. Dynamics of body weight in male rats (g, M±m).

Day	Groups		
	Control	Nano-SOD1 TD	Nano-SOD1 TDx10
0	259.8±3.08	256.5±2.19	260.6±2.93
7	302.0±6.75	303.6±7.63	309.6±6.03
14	337.3±8.77	326.7±6.07	338.2±5.74
21	361.6±8.71	351.7±3.82	362.4±6.18
28	362.9±10.63	360.0±5.21	366.4±6.45
35	366.3±8.49	368.0±7.45	377.2±7.17
42	403.1±12.86	393.9±6.94	393.1±5.93
48	417.9±6.02	408.7±6.87	414.1±2.62
Total increase	+158.1	+152.2	+153.5

Table S79. Dynamics of female body weight (g, M±m).

Day	Groups		
	Control	Nano-SOD1 TD	Nano-SOD1 TDx10
0	247.3±4.29	241.6±8.34	245.6±7.78
7	252.9±3.86	254.0±7.34	257.8±6.77
15	265.2±3.25	266.5±2.60	262.8±2.33
Total increase	+17.9	+24.9	+17.2

Study of generative function of animals

Sexual behavior research

When observing animals immediately after grazing, it was found that the latent period and duration of sexual activity in males treated with Nano-SOD1 corresponded to the control males.

Table S80 shows the number of females who were dissected in each group, the number of females who were found to have sperm in vaginal smears, and the number of females who became pregnant. Based on these data, the fertility and pregnancy indices were calculated. In all experimental groups, the number of fertilized and pregnant females did not differ from the control group.

Table S80. Effect of Nano-SOD1 on rat fertility.

group number	Number of grazed females	Number of fertilized females	Number of pregnant females	Fertility index, %	Pregnancy index, %
1	14	13	13	93	100
2	14	14	14	100	100
3	14	14	13	93	93
4	14	13	12	86	92
5	14	14	13	93	93

Monitoring of pregnant females

During pregnancy, the behavior of the females of the experimental groups did not differ from the control ones. All the animals were healthy and did not show any anxiety or aggression. Before giving birth, each female built a nest.

Dynamics of body weight in rats during gestationгестации

During gestation, females were weighed once a week (Table S81). As can be seen from Table S82, females gained the same body weight during pregnancy. There were no significant differences between the control and experimental groups.

Autopsy results of pregnant females

Half of the pregnant females were euthanized on the 20th day of pregnancy. At the autopsy, the number of yellow bodies in the ovaries, implantation sites in the uterus, the number of live and dead fetuses, and the number of resorptions were counted. The results of the studies are presented in Tables S83 and S84.

Table S81. Dynamics of body weight in pregnant females (g, M±m).

Groups	Day			
	0	7	14	20-21
1	278,5±7,24	293,7±8,36	322,8±8,57	356,2±of 12.17
2	269,2±7,75	295,8±11,71	316,6±7,60	354,7±8,77
3	269,6±5,47	287,1±5,54	316,5±6,21	357,2±8,28
4	277,1±5,11	298,0±8,36	327,8±8,36	372,3±11,43
5	272,5±4,90	291,4±6,36	323,3±7,04	353,7±of 9.03

Table S82. Body weight gain of pregnant females (g, M±m).

Groups	Time interval (in days)			
	0 - 7	7 - 14	14 – (21-22)	Total growth
1	+15.2	+29.1	+33.4	+77.7
2	+26.6	+20.8	+38.1	+85.5
3	+17.5	+29.4	+40.7	+87.6
4	+20.9	+29.8	+79.3	+95.2
5	+18.9	+31.9	+30.4	+81.2

Table S83. Indicators of embryogenesis in female rats after instillation of Nano-SOD1 to males (M±m).

Indicators	Group Indicators		
	Control	Nano-SOD1 TD	Nano-SOD1 TDx10
	1	3	5
Autopsy results (per female)			
Number of yellow bodies	10.9±0.67	11.1±0.99	11.4±1.17
Number of implantation	10.4±0.81	10.4±0.75	10.9±1.06
Number of resorptions	0.3±0.18	0.4±0.30	0.6±0.20
Number of live fetuses	10.1±0.77	10.0±0.62	10.3±0.97
Number of dead fetuses	0	0	0
Preimplantation death %	4.4±2.90	5.4±2.64	4.6±13.72
Post-implantation death %	2.5±1.61	3.5±2.36	5.1±1.92
Fetus parameters (per female)			
Body weight, g	4.0±0.10	3.8±0.05	3.9±0.06
Craniocaudal size, cm	5.4±0.07	5.5±0.04	5.6±0.03

Table S84. Demonstration of embryogenesis in rats after instillation of Nano-SOD1 to females (M±m).

Indicators	Group		
	Control	Nano-SOD1 TD	Nano-SOD1 Tdx10
	1	2	4
Autopsy results (per female)			
Number of yellow bodies	10.9±0.67	12.3±0.92	11.0±0.37
Number of implantation	10.4±0.81	10.4±0.37	10.5±0.43
Number of resorptions	0.3±0.18	0.3±0.18	0.5±0.34
Number of live Fetuses	10.1±0.77	10.1±0.40	10.0±0.52
Number of dead Fetuses	0	0	0
Preimplantation death, %	4.4±2.90	12.4±6.38	4.2±4.17
Post-implantation death, %	2.5±1.61	2.7±1.76	4.7±3.15

Indicators	Group		
	Control	Nano-SOD1 TD	Nano-SOD1 Tdx10
	1	2	4
Fetus parameters (per female)			
Body weight, g	4.0±0.10	3.8±0.07	3.8±0.09
Craniocaudal size, cm	5.4±0.07	5.5±0.05	5.5±0.04

From the data of Tables S83, S84, it can be seen that instillations of Nano-SOD1 in the conjunctival sac of the eye before mating animals, both in the therapeutic dose and 10 times its excess, did not cause a negative effect on the formation and development of embryos in the antenatal period of development.

Study of fetal developmental disorders

To identify possible developmental disorders of embryos after the use of the test drug, external abnormalities of fetal development, as well as internal organs according to the Wilson method and the state of the skeleton according to the Dawson method were studied.

During external examination, no developmental abnormalities were detected in the embryos (Tables S85 and S86).

When examining fetuses using the Wilson method, no internal organ abnormalities were detected in any embryo (Tables S87 and S88).

The offspring of rats of all groups did not have an atypical structure and location of skeletal bones. In the axial skeleton, the process of calcification was noticeable in almost all parts of the spine, with the exception of the caudal region, ossification of most of the vertebrae of which was not carried out during intrauterine development. No abnormalities in the development of vertebrae, sternum, or pelvic bones were found. There was no difference in the number of ossification centers in the metatarsal and metacarpal bones (Tables S89 and S90).

Table S85. Results of evaluation of fetal condition during external examination after instillation of Nano-SOD1 in male rats.

Indicators	Group Indicators		
	Control	Nano-SOD1 TD	Nano-SOD1 TDx10
	1	3	5
Number of fetuses examined, abs.	71	70	72
With developmental abnormalities, abs/%	0/0	0/0	0/0
With subcutaneous hemorrhages, abs/%	0/0	0/0	2/2.8

Table S86. Results of evaluation of fetal condition during external examination after instillation of Nano-SOD1 to female rats.

Indicators	Group Indicators		
	Control	Nano-SOD1 TD	Nano-SOD1 TDx10
	1	2	4
Number of fetuses examined, abs.	71	71	60
With developmental abnormalities, abs/%	0/0	0/0	0/0
With subcutaneous hemorrhages, abs/%	0/0	1/1.4	0/0

Table S87. Results of evaluation of fetal internal organs after instillation of Nano-SOD1 in male rats.

Indicators	Group Indicators		
	Control	Nano-SOD1 TD	Nano-SOD1 TDx10
	1	3	5
Number of fetuses examined, abs.	24	24	24
With developmental abnormalities, abs/%	0/0	0/0	0/0

Table S88. Results of evaluation of fetal internal organs after instillation of Nano-SOD1 for female rats.

Indicators	Group Indicators		
	Control	Nano-SOD1 TD	Nano-SOD1 TDx10
	1	2	4
Number of fetuses examined, abs.	24	24	20
With developmental abnormalities, abs/%	0/0	0/0	0/0

Table S89. Results of evaluation of fetal skeleton condition after instillation of Nano-SOD1 in male rats (M±m).

Indicators	Group Indicators		
	Control	Nano-SOD1 TD	Nano-SOD1 TDx10
	1	3	5
Number of examined fetuses	47	46	48
Ossified metatarsal bones	3.8±0.06	3.7±0.08	3.8±0.05
Ossified distal phalanges of the forelimbs	3.3±0.10	3.5±0.11	3.3±0.11
Ossified proximal phalanges of the forelimbs	0.2±0.08	0.4±0.13	0.3±0.09
Ossified metacarpal bones	3.6±0.09	3.7±0.09	3.6±0.08
Ossified distal phalanges of the hind limbs	2.9±0.12	2.9±0.14	2.9±0.12
Ossified proximal phalanges of the hind limbs	0.2±0.07	0.2±0.09	0.2±0.07

Table S90. Results of evaluation of fetal skeleton condition after instillation of Nano-SOD1 in female rats (M±m).

Indicators	Group Indicators		
	Control	Nano-SOD1 TD	Nano-SOD1 Tdx10
	1	2	4
Number of examined fetuses	47	47	40
Ossified metatarsal bones	3.8±0.06	3.8±0.06	3.9±0.06

Indicators	Group Indicators		
	Control	Nano-SOD1 TD	Nano-SOD1 Tdx10
	1	2	4
Ossified distal phalanges of the forelimbs	3.3±0.10	3.4±0.09	3.4±0.11
Ossified proximal phalanges of the forelimbs	0.2±0.08	0.3±0.09	0.3±0.10
Ossified metacarpal bones	3.6±0.09	3.7±0.08	3.6±0.09
Ossified distal phalanges of the hind limbs	2.9±0.12	3.0±0.13	2.9±0.14
Ossified proximal phalanges of the hind limbs	0.2±0.07	0.2±0.08	0.3±0.09

Studying the physical development of offspring

Half of the pregnant females were left to give birth, in order to monitor the survival and development of the offspring. Tables S91 and S92 show data on the effect of Nano-SOD1 on the duration of pregnancy and the condition of fetuses.

The duration of pregnancy and the number of pups born per pregnant female were not statistically different between the groups.

One day after delivery, 8 pups were left in large litters for optimization.

After birth, the pups were monitored for a month. Data on the physical development of the population are presented in Tables S93 and S94. There was no statistical difference between the experimental and control groups, both in the dynamics of body weight and in the development of pups.

Table S91. Average values of offspring born from females mated with males treated with Nano-SOD1 (M±m).

Indicators	Group		
	Control	Nano-SOD1 TD	Nano-SOD1 TDx10
Group	1	3	5
Duration of pregnancy, days	21.8±0.31	21.9±0.26	22.0±0.26
Number of pups born in the litter	9.2±0.79	8.7±0.89	9.2±0.31
Number of live rat pups in the litter	9.0±0.78	8.1±0.89	9.0±0.45
Number of stillborn baby rats in the litter	0.2±0.17	0.6±0.43	0.2±0.17

Indicators	Group		
	Control	Nano-SOD1 TD	Nano-SOD1 TDx10
Body weight at birth, g	6.1±0.26	6.5±0.40	6.7±0.24
Craniocaudal size at birth, cm	6.4±0.13	6.8±0.09	6.8±0.15

Table S92. Average parameters of offspring born from females treated with Nano-SOD1 (M±m).

Indicators	Group		
	Control	Nano-SOD1 TD	Nano-SOD1 TDx10
Group	1	2	4
Duration of pregnancy, days	21.8±0.31	21.5±0.22	21.5±0.22
Number of pups born in the litter	9.2±0.79	8.8±0.95	9.7±0.42
Number of live rat pups in the litter	9.0±0.78	8.7±0.88	9.3±0.49
Number of stillborn baby rats in the litter	0.2±0.17	0.2±0.17	0.3±033
Body weight at birth, g	6.1±0.26	6.9±0.40	6.2±0.32
Craniocaudal size at birth, cm	6.4±0.13	6.9±0.24	6.6±0.11

Table S93. Average indicators of physical development of offspring born to females who were mated with males treated with Nano-SOD1 (M±m).

Parameters		Dosage		
		Control	Nano-SOD1 TD	Nano-SOD1 TDx10
Group		1	3	5
Offspring survival index, %		98.2	92.2	98.2
The mass of rats, g	1st day	6.1±0.26	6.5±0.40	6,7±0,24
	4th day	9.8±0.33	9.8±0.91	10,6±0,40
	7 days	16.3±2.14	15.1±0.49	14,3±0,71
	14 days	29.4± 3.04	29.1±1.83	29,0±1.43
	21 day	44.2±3.60	48.3±3.12	49,5± 2.00
	28-day	74.3±4.94	87.5± 5.92	88,1±2.70
	35 days	115.4±5.69	131.2± 8.22	126,3±3,96
	Total increase	109.3	124.7	112.2
Detachment of the auricle, day		3,8±0,08	3.8±0.31	3.2±0.31
Appearance of primary coat, day		8.0±0.07	7.6±0.18	87.7±0.33
Teething, day		6.0±0.07	6.0±0.00	6.0±0.02
Eye opening, day		15.2±0.19	15.5±0.18	15.7±0.0.20
Lowering of testicles, day		24,9±0,22	25.2±0.08	25.0±0.00
Opening of the vagina, day		33,5±0,22	32.6±0.40	33.3±0.40

Table S94. Average indicators of physical development of offspring born to females treated with Nano-SOD1 (M±m).

Parameters		Dosage		
		Control	Nano-SOD1 TD	Nano-SOD1 TDx10
Group		1	2	4
Offspring survival index, %		98.2	98.4	96.6
The mass of rats, g	1st day	6.1±0.26	6.9±0.40	6,2±0,32
	4th day	9.8±0.33	11.0±0.66	10,0±0,69
	7 days	16.3±2.14	15.8± 1.00	16,3± 1.03
	14 days	29.4± 3.04	30.1± 1.79	31,4±1,33
	21 day	44.2±3.60	49.1±2.94	50,4±1.49
	28 day	74.3±4.94	87.4±4.40	89,9±2.31
	35 days	115.4± 5.69	121.6±8.59	120,5±4,46
	Total increase	109.3	114.7	114.3
Detachment of the auricle, day		3,8±0,08	3.4±0.28	3.6±0.17
Appearance of primary coat, day		8.0±0.07	7.5±0.19	7.9±0.25
Teething, day		6.0±0.07	6.1±0.04	6.0±0.02
Eye opening, day		15.2±0.19	15.7±0.45	15.6±0.19
Lowering of testicles, day		24,9±0,22	25.2±0.10	25.0±0.10
Opening of the vagina, day		33,5±0,22	33.8±0.34	33.5±0.17

Thus, we can conclude that Nano-SOD1 formulations obtained in this work did not cause any eye irritation in rabbits at instillations, did not exhibit acute and chronic toxicity even at high doses, did not cause allergic reactions or affect reproductive ability of the animals and did not possess neither mutagenic nor immunogenic properties at the doses used in the work.

8. References

1. Gad, S.C. Rodents model for toxicity testing and biomarkers. In Biomarkers in Toxicology; Elsevier, 2014; pp. 7–69 ISBN 9780124046306.
2. Faqi, A.S. A comprehensive guide to toxicology in nonclinical drug development; Faqi, A.S., Ed.; Academic Press, 2017; ISBN 9780128036204.
